# Supplementary material for: Metabolic beneficial effects of targeting a long non-coding RNA, lnc-megacluster, in obesity
Source: Mol Ther Nucleic Acids. 2025 Dec 8;37(1):102792. doi: 10.1016/j.omtn.2025.102792 (PMC12794071; doi:10.1016/j.omtn.2025.102792)
Supplement: Document S1. Figures S1–S17 and Tables S1–S4 [file mmc1.pdf]

OMTN, Volume *37*

Supplemental information

Metabolic beneficial effects of targeting a long non-coding RNA, lnc-megacluster, in obesity

Maryam Abdollahi, Vajir Malek, Vinay Singh Tanwar, Mitsuo Kato, Linda Lanting, Alireza Rezaei, Lingxiao Zhang, Lixin Yang, Raju K. Pillai, Leah Kebrom, Jathan Nandi, Wendong Huang, Ke Ma, and Rama Natarajan

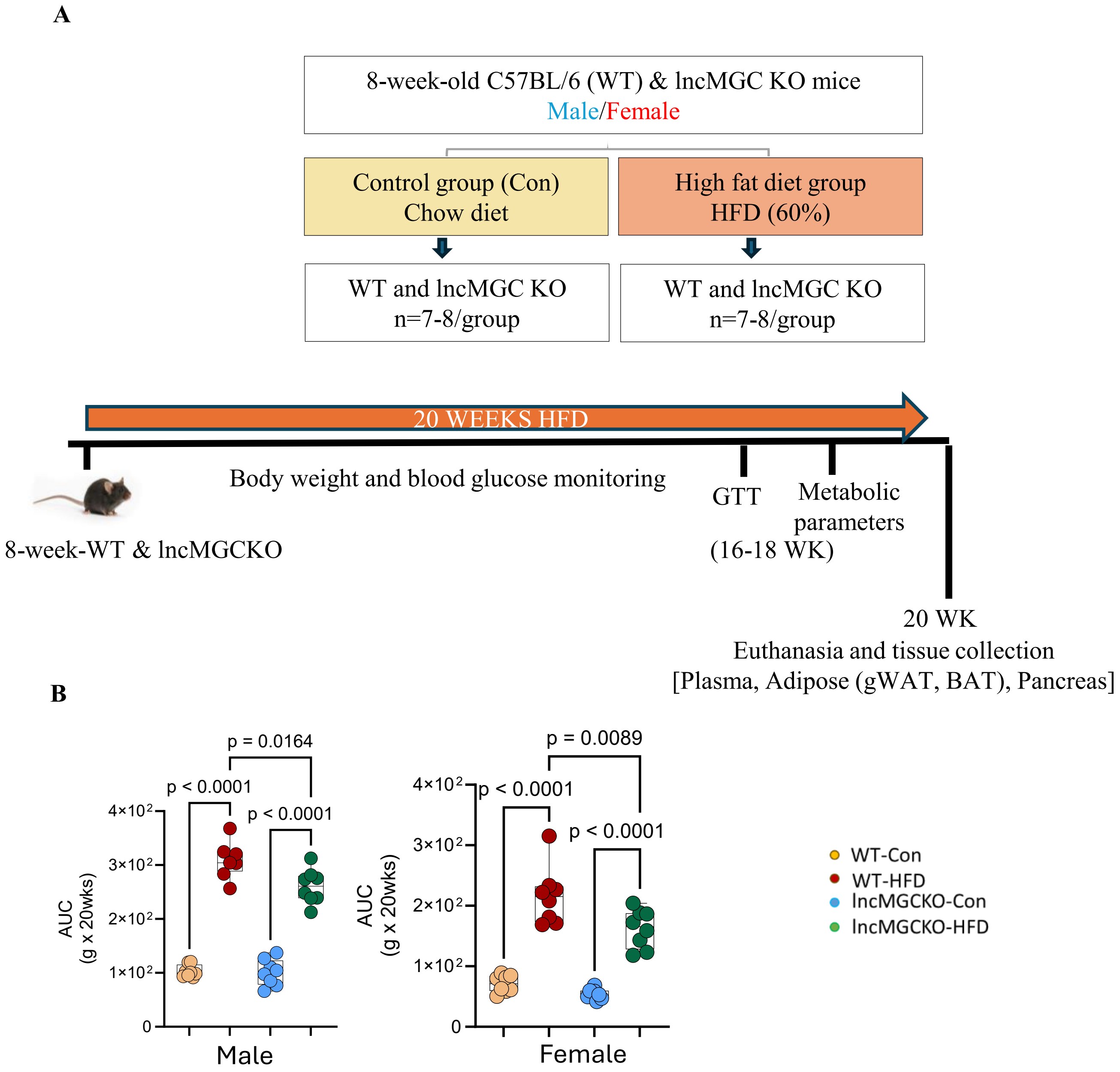

Figure S1. Experimental scheme for tracking high-fat diet (HFD) fed induced- obesity in WT and lncMGC KO mice. (A) Eight-week-old female and male wild type (WT) C57BL/6J mice and lncMGC (KO) mice were randomly divided into groups and fed with laboratory control chow-diet (Con) or high-fat diet (HFD) (60% kcal from fat) for 20 weeks. Body weights and blood glucose levels were monitored biweekly. Glucose tolerance tests (GTT) were performed, and metabolic parameters, including body composition, food and water intake, movement, oxygen consumption, and energy expenditure, were measured. Total HFD period was 20 weeks, but metabolic parameters and GTT were measured at 16-18 weeks of HFD. After euthanasia, plasma, perigonadal white adipose tissue (gWAT), interscapular brown adipose tissue (BAT), and pancreata were harvested. (B) Body weight gain was calculated as the increase from initial body weight using the formula:[(Final body weight – Initial body weight) / Initial body weight], and the area under the curve (AUC) was shown in the graphs. Statistical analyses were performed by Two-way ANOVA with post-hoc Tukey test for multiple comparisons. The bar and whisker plot displays the distribution of the data. The whiskers extend from the minimum to the maximum values. Individual data points are overlaid as dots. Statistically significant p-values are indicated in the bar graphs.

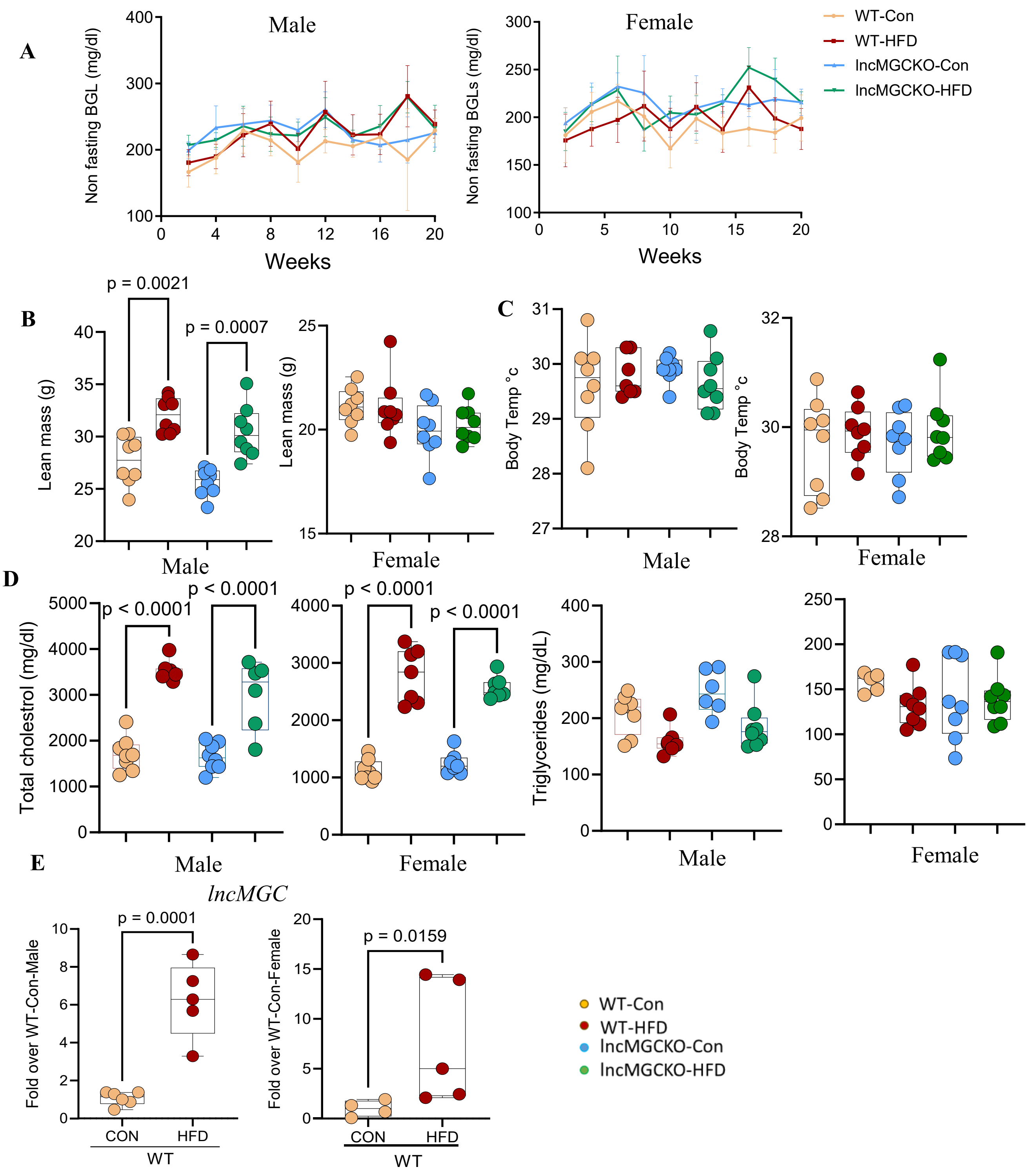

Figure S2. Monitoring blood glucose and other parameters. (A) Average non-fasting blood glucose levels (BGLs) of male and female mice over 20 weeks of diets. (B) Lean mass in male and female mice at 18 weeks of diets. (C) peripheral body temperature. (D) Plasma total cholesterol and triglyceride. n=7-8/group. (E) Gene expression of lncMGC in isolated stromal vascular fraction (SVF) from gWAT in WT and HFD male and female mice (n=4-6/group). Statistical analyses were performed by Two-way ANOVA with post-hoc Tukey test for multiple comparisons. XY graphs show the mean (SD). The bar and whisker plot displays the distribution of the data. The whiskers extend from the minimum to the maximum values. Individual data points are overlaid as dots. Statistically significant p-values are indicated in the bar graphs.

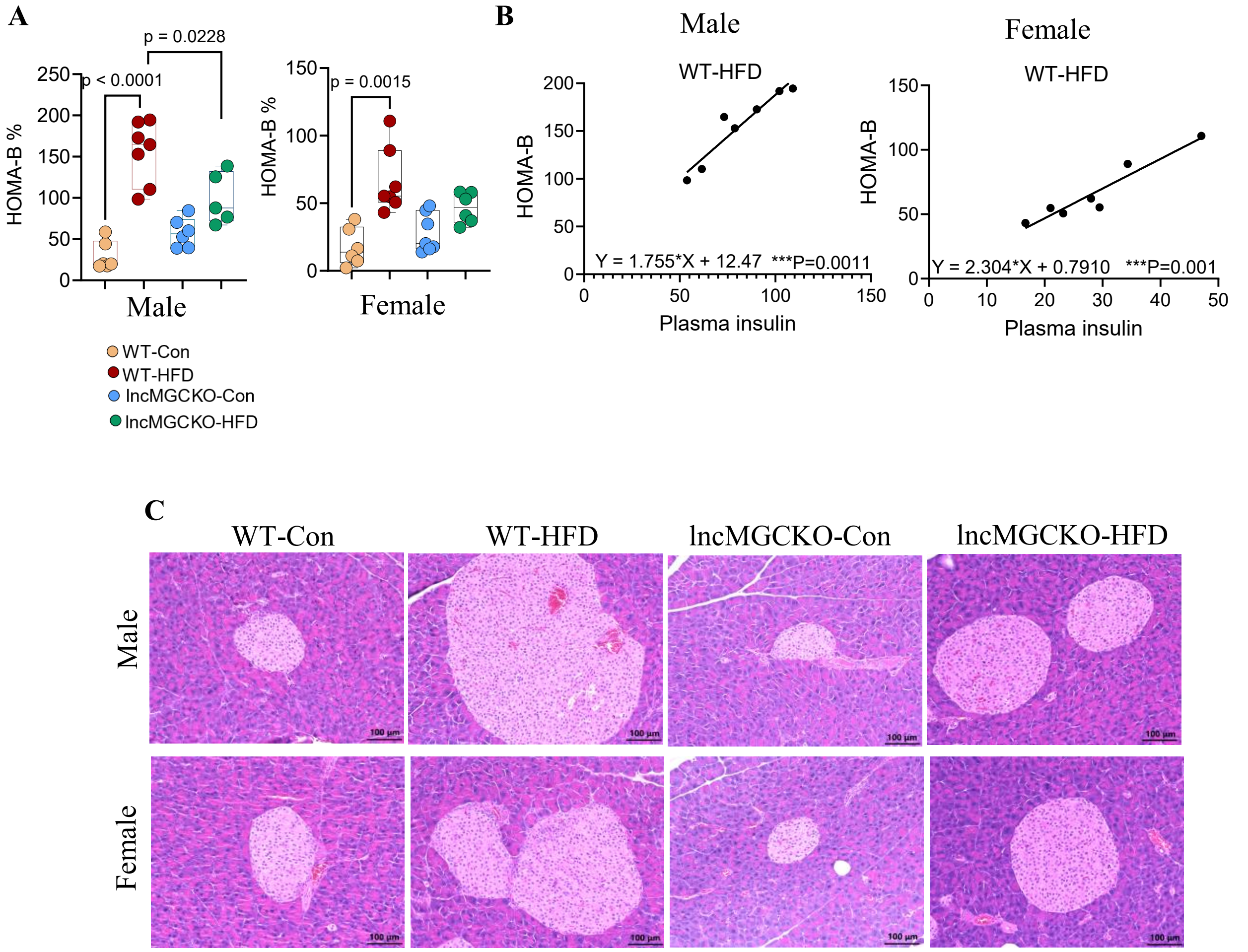

Figure S3. HFD-induced beta cell dysfunction and islet hypertrophy were improved in lncMGC KO mice. (A) Percentage of Homeostasis Model Assessment (HOMA) analysis for β-cell function (HOMA-B) in WT and lncMGC KO Con and HFD mice; (B) Correlation between beta cell function (HOMA B%) and plasma insulin levels in wild type (WT) HFD male and female mice. (C) Representative H &E staining images show islet hyperplasia and hypertrophy in WT HFD mice, which is improved in lncMGC KO HFD mice (male and female). Scale bars, 50 µm. n=5-8/group. Statistical analyses were performed by Two-way ANOVA with post-hoc Tukey test for multiple comparisons. The bar and whisker plot displays the distribution of the data. The whiskers extend from the minimum to the maximum values. Individual data points are overlaid as dots. Statistically significant p-values are indicated in the bar graphs.

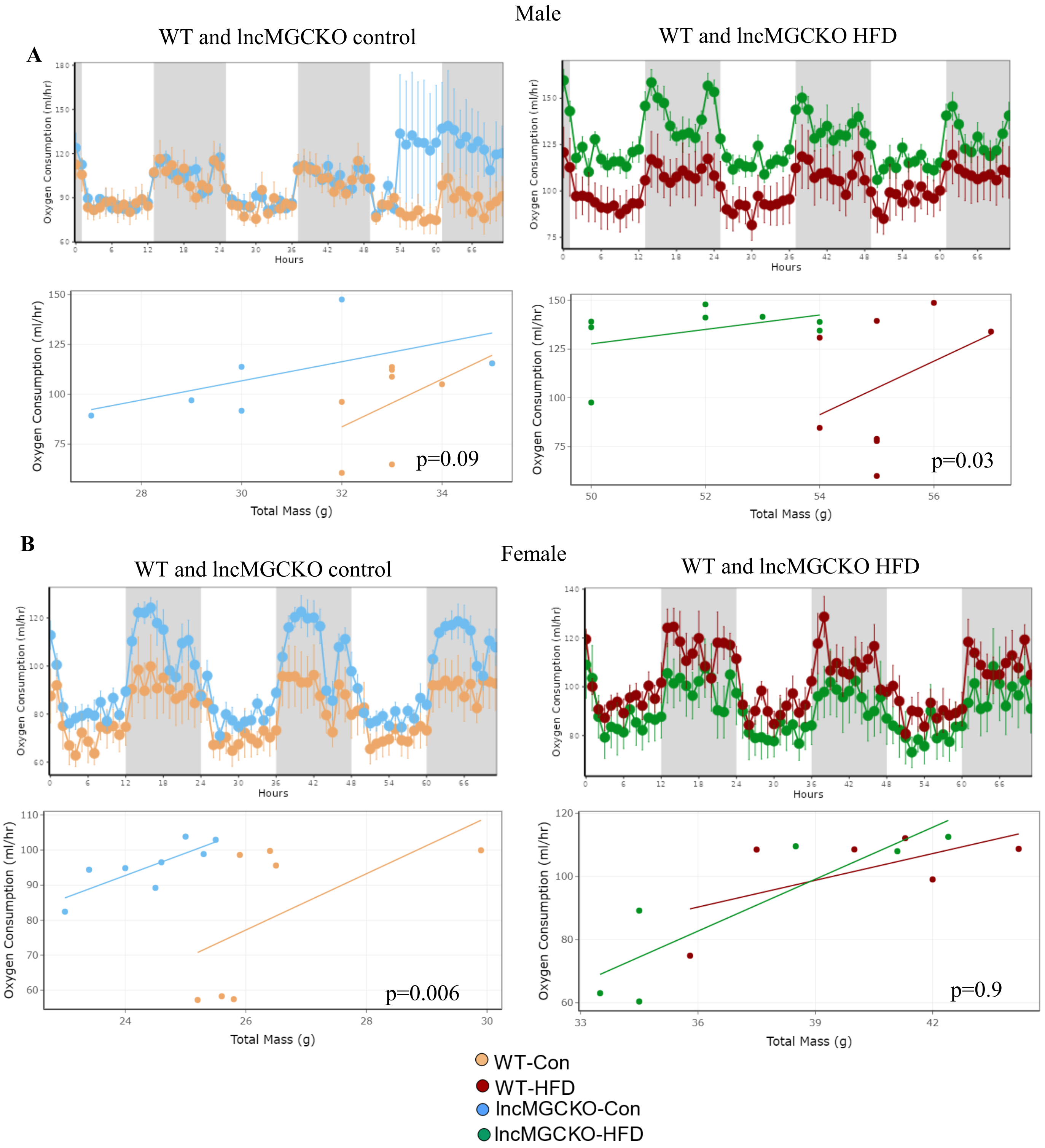

Figure S4. lncMGC KO mice exhibit increased oxygen consumption. Oxygen consumption (VO2) and regression analysis of VO2 and total body mass in WT and lncMGC KO Con, and WT and lncMGC KO HFD (A) male and (B) female mice. n=6-8. Data were analyzed for VO2 and total mass regression using ANCOVA with VO2 as a dependent variable, genotype as fixed variable, and body mass as a covariate. Night-time (gray-shades). P-values are indicated in the figures.

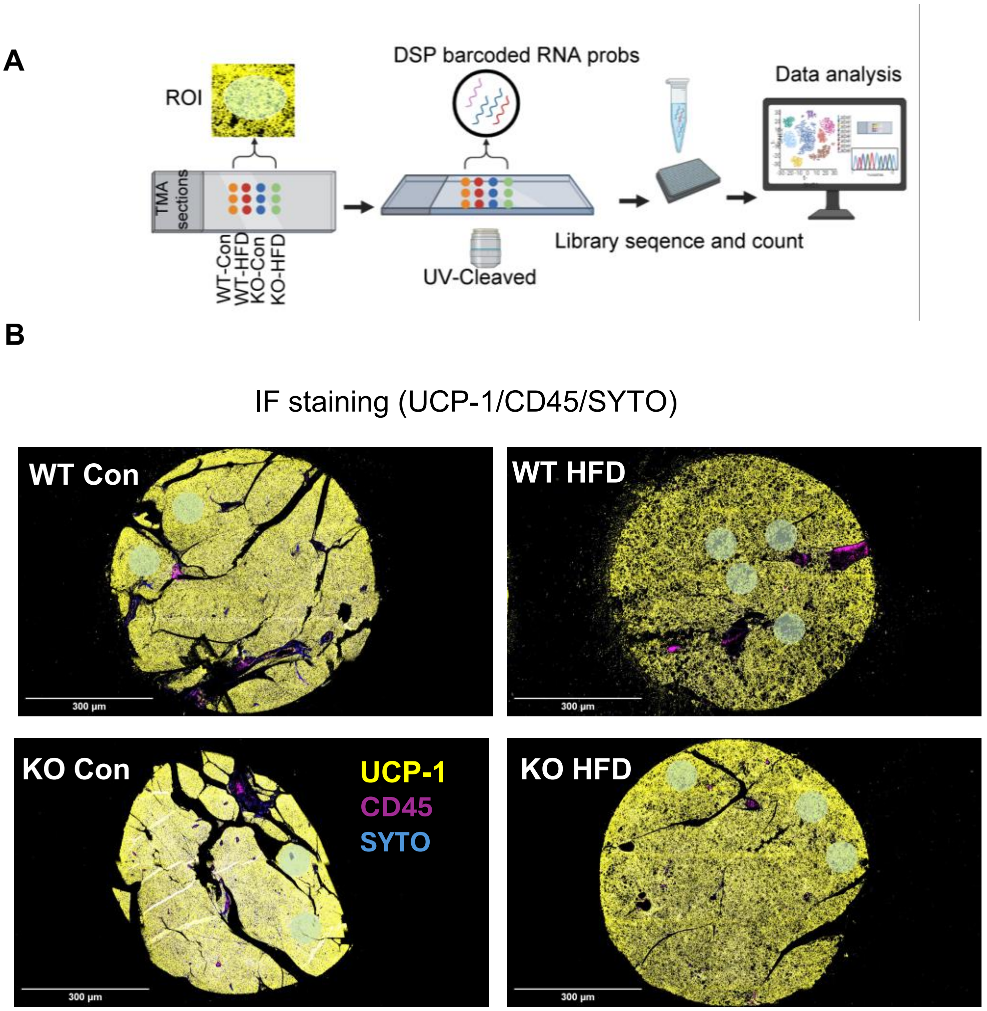

Figure S5. Nanostring Digital gene expression profiling of BAT tissues. Schematic illustration of the workflow of the Nanostring GeoMx Digital Spatial Profiler (DSP) Whole Transcriptome Atlas (WTA), which was used for the interrogation of multiple RNA analytes from a single paraffin-embedded brown adipose tissue (BAT) section from the mice groups. (B) Representative IF staining images of Tissue Microarray (TMA) sections from BAT isolated from WT and lncMGC KO Con and HFD female mice. 20 weeks HFD. The sections were stained with UCP-1(mitochondrial marker, yellow signals), CD45 (leukocytes marker, purple signals), and SYTO (nucleic acid stain, blue signal) for the selection of regions of interest (ROIs, bubble plots). Analytes in the TMA sections are conjugated with oligonucleotide (oligo) tags using a photocleavable linker. Spatially mapped UV illumination enables the release of oligo tags from the analyte into the plates. Sequencing was performed to obtain quantitative counts per ROI. Scale bar: 300 µm. N=3 mice/group. The number of selected ROIs is as follows: n=4/WT-Con, n=6\WT-HFD, n=5/lncMGC KO Con, and n=7/lncMGC KO HFD.

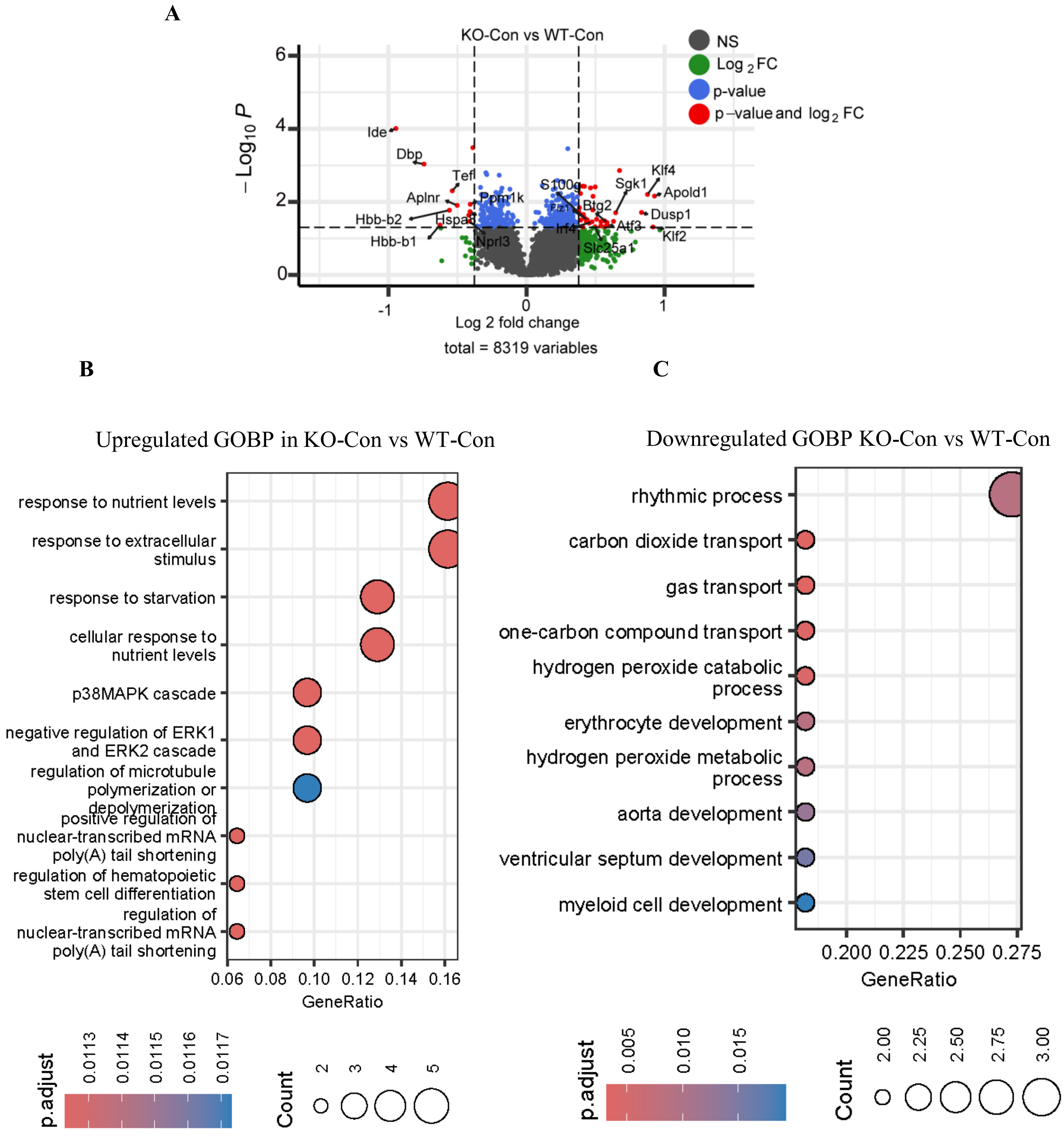

Figure S6. Gene ontology biological process in brown adipose tissue from control chow diet female mice. (A) Differential gene expression in lncMGC KO Con compared to WT Con female mice. (B)Top enriched up-regulated gene ontology biological process (GOBP) in lncMGC KO Con compared to WT Con and (C) Top enriched down-regulated GOBP in lncMGC KO Con compared to WT Con female mice. p-value<0.05.

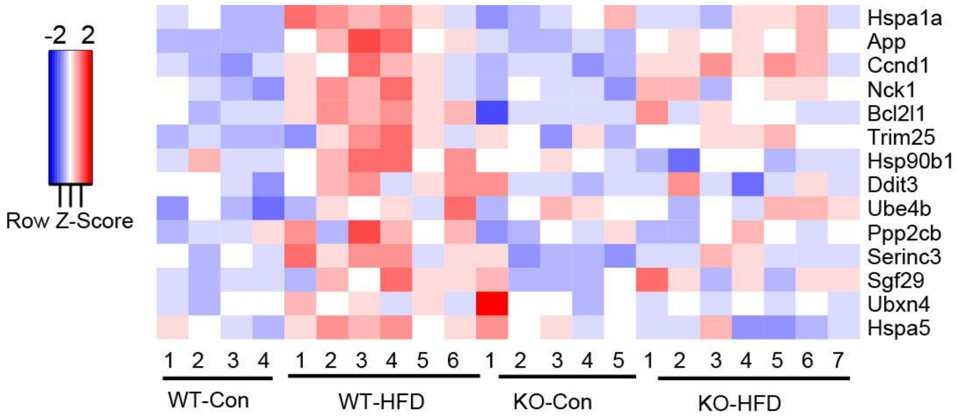

Figure S7. Factors related to endoplasmic reticulum stress are improved in brown adipose tissue of lncMGC KO HFD female mice. Heatmap showing key genes related to endoplasmic reticulum (ER) stress were significantly modulated between regions of interest (ROIs) in UCP-1 positive segments across all groups; normalized Q3 values were used to generate the heatmap. WT-Con=4 ROI, WT-HFD=6 ROI, lncMGC Con=5 ROI, lncMGCKO HFD=7 ROI. Data analysis was performed using R. Log2 fold change ±0.378 and p-value<0.05. The columns of the heatmap represent indicated genes, showing markedly altered expression levels as indicated by fold change.

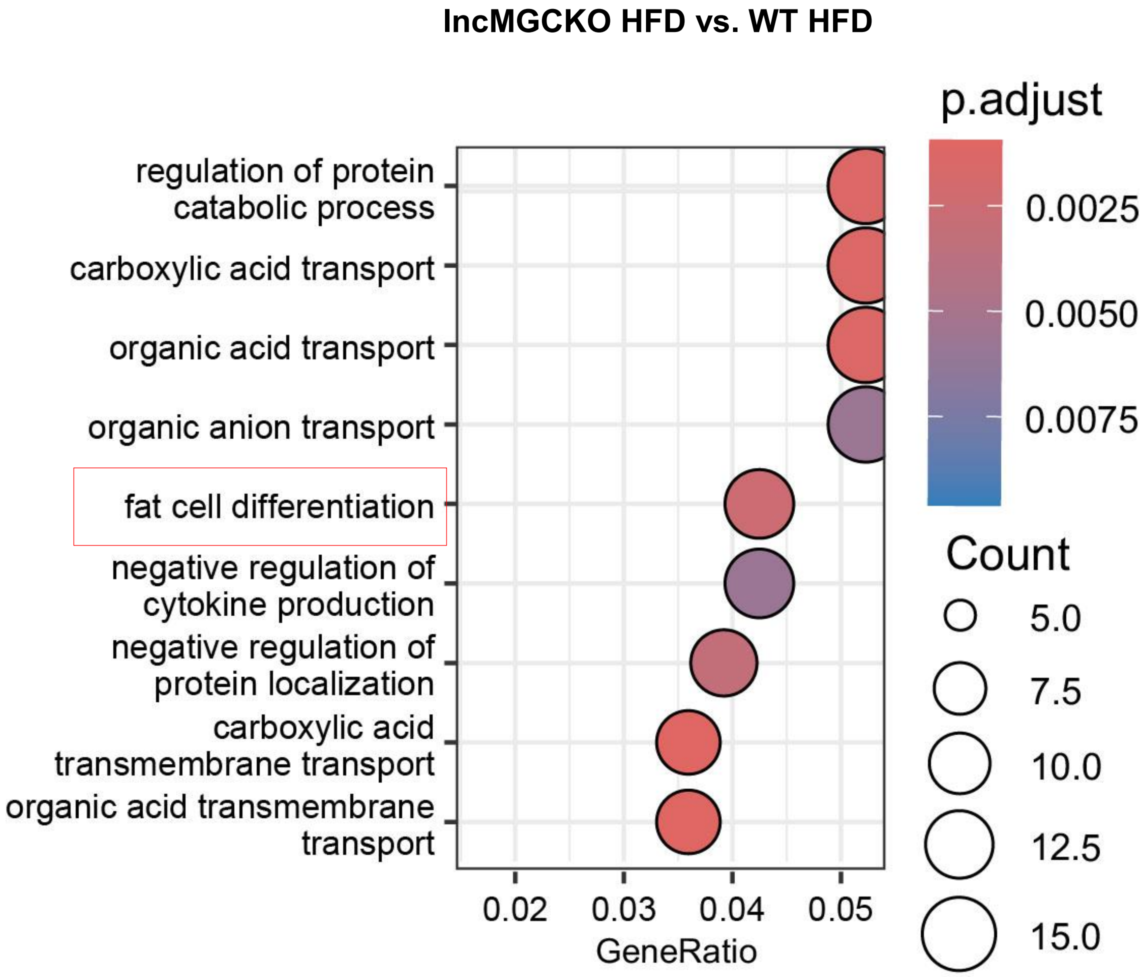

Figure S8. Gene ontology biological process in brown adipose tissue from WT and lncMGCKO HFD female mice. Top enriched up-regulated gene ontology biological process (GOBP) in lncMGC KO HFD compared to WT HFD. p-value<0.05.

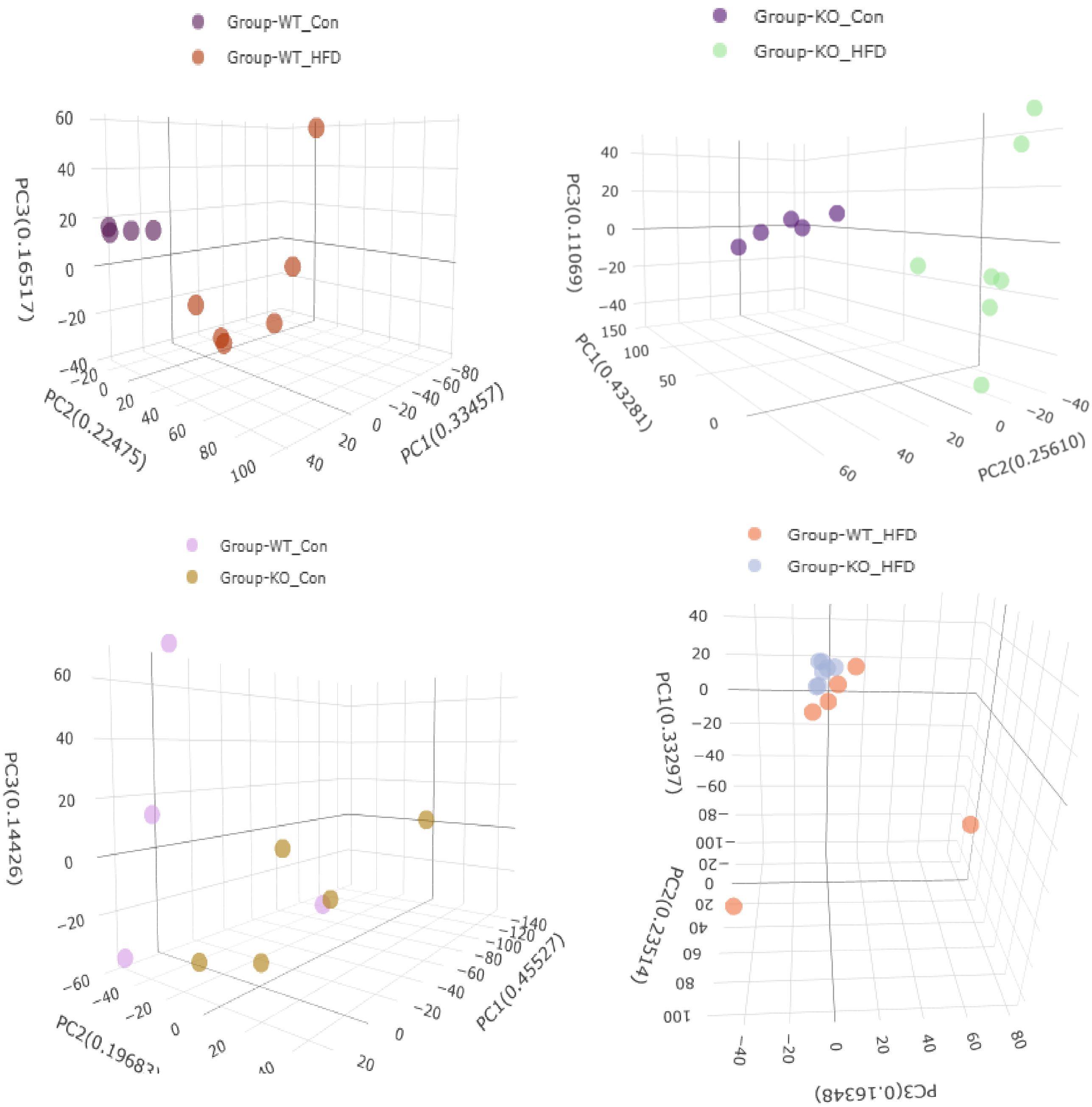

Figure S9. Principal component analysis (PCA) of RNA-seq data from brown adipose tissue. PCA was performed on the transcriptomic profiles of all biological replicates from each experimental group. The plot shows separation between groups based on gene expression variance along PCA 1,2, and 3, indicating distinct transcriptional signatures. Wild type (WT) control (Con), WT-high fat diet (HFD), lncMGCKO Con, lncMGCKO HFD

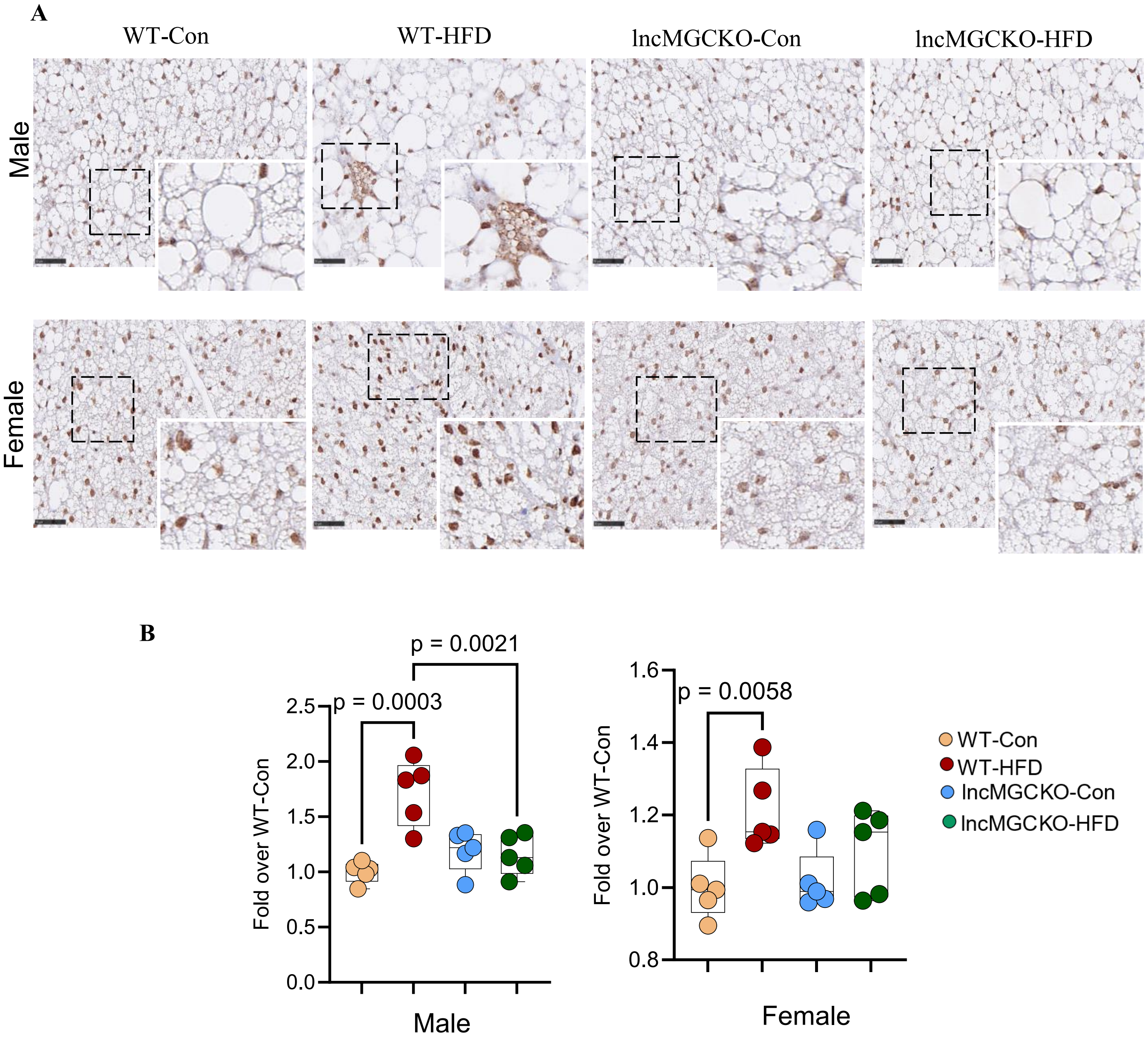

Figure S10. lncMGC KO improved expression of ER stress marker CHOP in brown adipose tissue of HFD mice. (A) Immunohistochemistry staining and (B) quantitative analysis of CHOP in BAT in male and female mice. 20 weeks of HFD. n=5/group. Statistical analyses were performed by Two-way ANOVA with post-hoc Tukey test for multiple comparisons. The bar and whisker plot displays the distribution of the data. The whiskers extend from the minimum to the maximum values. Individual data points are overlaid as dots. Statistically significant p-values are indicated in the bar graphs. The magnification indicated by the lines in the IHC images represents the selected area that has been enlarged and shown at a higher resolution.

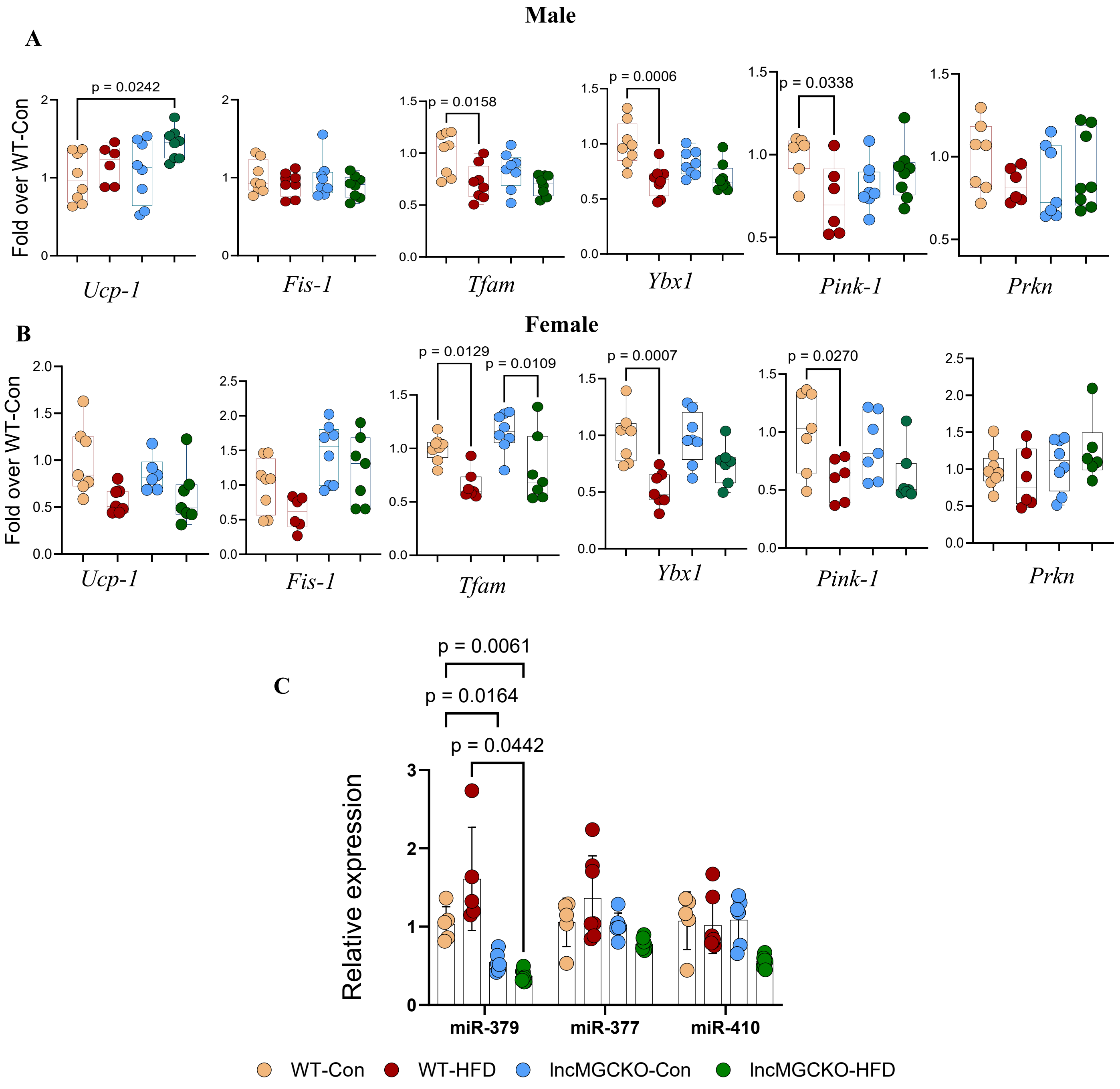

Figure S11. Gene expression of mitochondrial markers in brown adipose tissue. Gene expression of indicated mitochondrial markers in (A) male and (B) female mice. (n= 6-8 mice/group). (C) Expression of some of the cluster micro RNAs (miRs) in the brown adipose tissues in WT and lncMGCKO control and HFD male mice. 20 weeks HFD. Statistical analyses were performed by Two-way ANOVA with post-hoc Tukey test for multiple comparisons. The bar and whisker plot displays the distribution of the data. The whiskers extend from the minimum to the maximum values. Individual data points are overlaid as dots. Bar graphs show the mean (SD). Statistically significant p-values are indicated in the bar graphs.

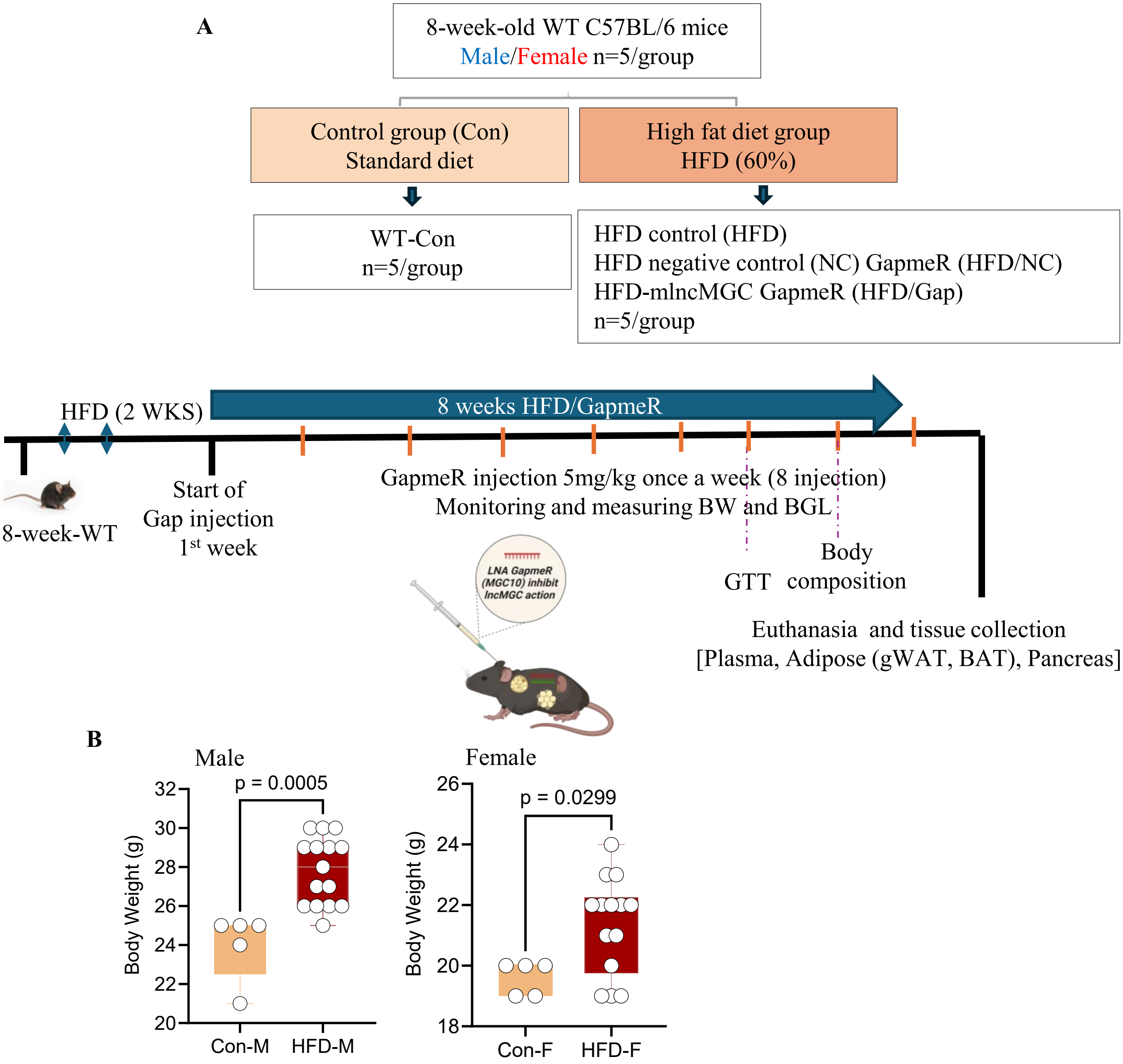

Figure S12. Experimental scheme for treatment with GapmeR targeting mouse lncMGC in high-fat diet (HFD) fed induced- obesity mice model. (A) 8-wk-old female and male wild type (WT) C57BL/6J mice were randomly divided into groups and fed with laboratory control chow diet (Con) or high-fat diet (HFD) (60% kcal from fat) for 2 weeks. Then, mice under HFD were randomly divided into 3 groups and injected with negative control (NC), GapmeR (HFD/NC), or GapmeR targeting mouse lncMGC (HFD/Gap) at 5 mg/kg body weight once a week for 8 weeks. HFD-fed mice without treatment were used as control (HFD). Body weights (BW) and blood glucose levels (BGL) were monitored weekly during 10 weeks of diets. Glucose tolerance tests (GTT) were performed, and body composition was measured at 10 weeks of study. After euthanasia, plasma, perigonadal white adipose tissue (gWAT), and interscapular brown adipose tissue (BAT) and pancreata were harvested. (B) Average body weights after the first 2 weeks of HFD. Statistical comparisons between two groups were performed using two-sided Student's t-tests. The whiskers extend from the minimum to the maximum values. Individual data points are overlaid as dots. P-values are indicated in the bar graphs.

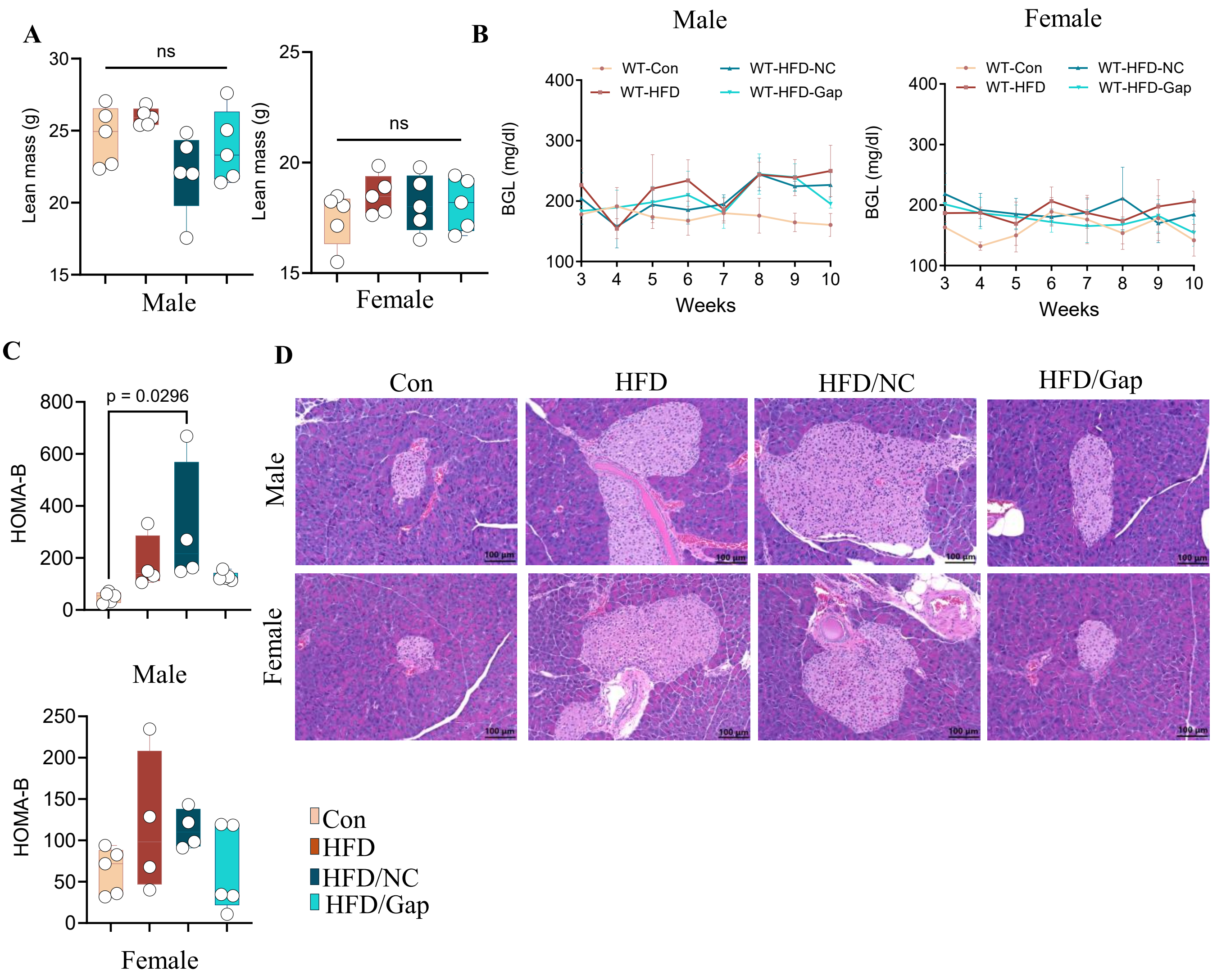

Figure S13. GapmeR targeting lncMGC improved beta cell function in HFD-fed mice. (A) Lean mass in male and female mice at 10 weeks of diets. (B) Average non-fasting blood glucose levels (BGL) of male and female mice over 10 weeks of diets. (C) Percentage of Homeostasis Model Assessment (HOMA) analysis for β-cell function (HOMA-B) in male and female mice. (D) H&E-stained sections showing islets hyperplasia and hypertrophy in HFD and HFD/NC mice and improvement in HFDGap mice. Control chow-diet (Con), high-fat diet (HFD), negative control GapmeR (HFD-NC) and GapmeR targeting lncMGC (HFD-Gap). n=4-5/group. Statistical analyses were performed by Two-way ANOVA with post-hoc Tukey test for multiple comparisons. XY graphs show the mean (SD). The bar and whisker plot displays the distribution of the data. The whiskers extend from the minimum to the maximum values. Individual data points are overlaid as dots. Statistically significant p-values are indicated in the bar graphs.

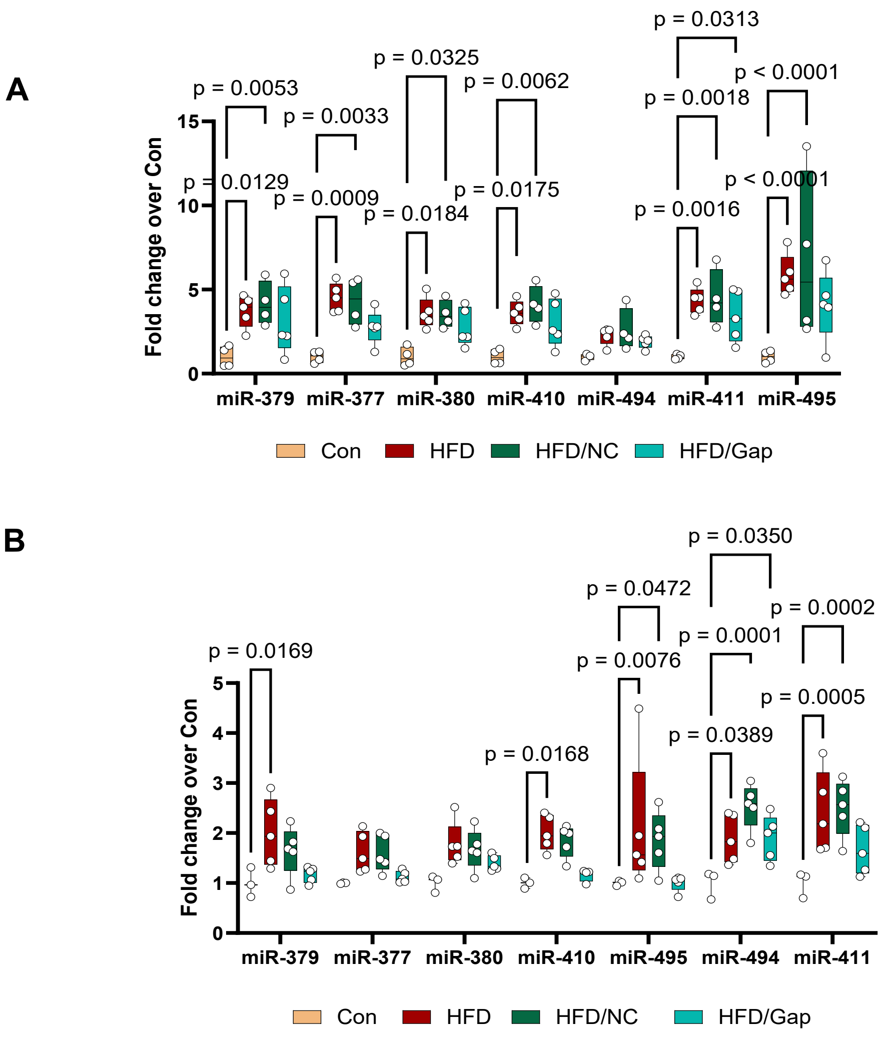

Figure S14. Expression of some of the cluster micro RNAs (miRs) in the perigonadal white adipose tissues from (A) male and (B) female mice treated with or without GapmeRs.10 weeks HFD. Control chow-diet (Con), high-fat diet (HFD), negative control (NC) GapmeR (HFD/NC) and GapmeR targeting lncMGC (HFD/Gap). Statistical analyses were performed by two-way ANOVA with a post-hoc Tukey test for multiple comparisons. n=4-5. The bar and whisker plot displays the distribution of the data. The whiskers extend from the minimum to the maximum values. Individual data points are overlaid as dots. Statistically significant p-values are indicated in the bar graphs.

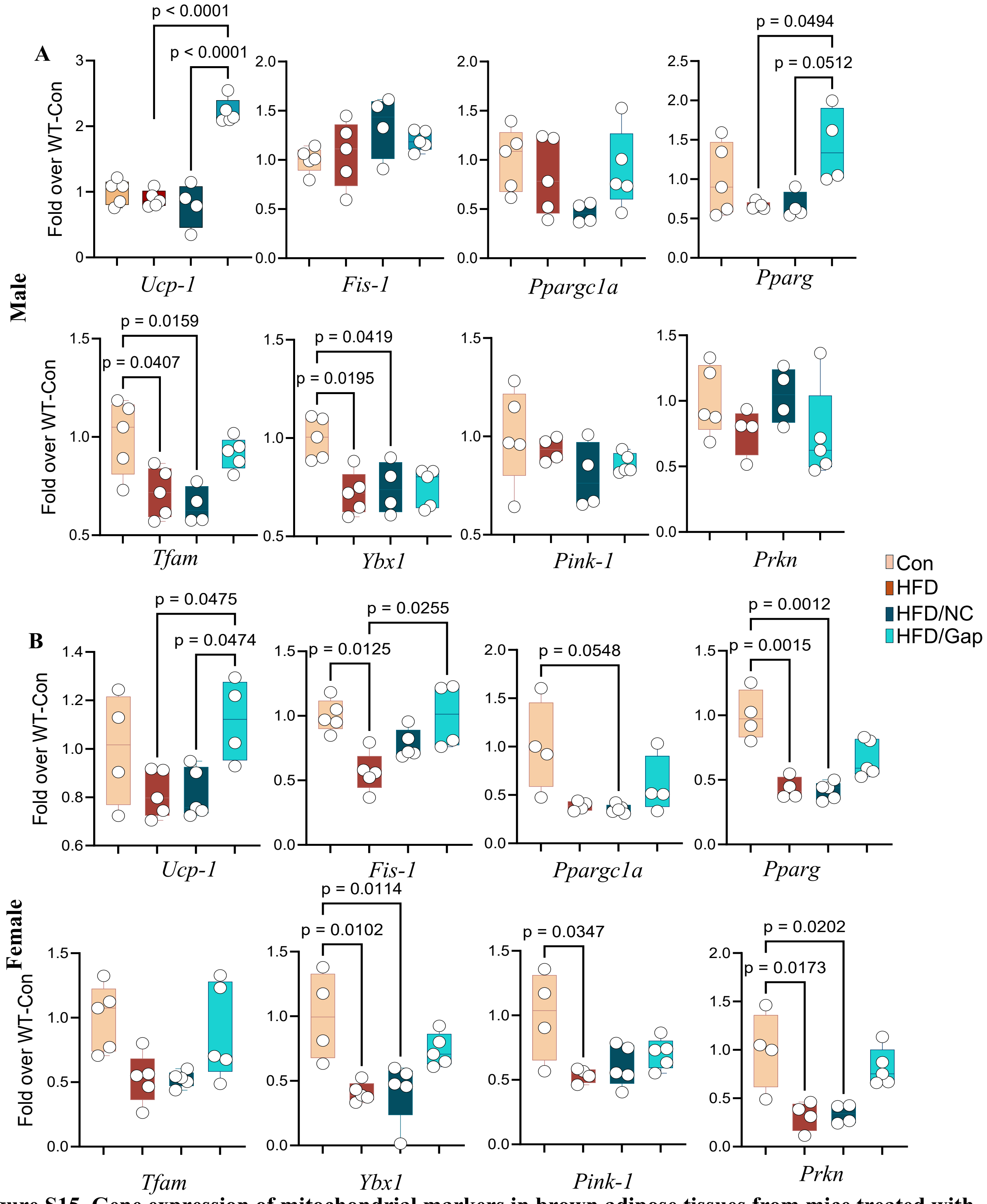

Figure S15. Gene expression of mitochondrial markers in brown adipose tissues from mice treated with or without GapmeRs. Gene expression of mitochondrial markers in (A) male and (B) female mice. 10 weeks HFD. Statistical analyses were performed by Two-way ANOVA with post-hoc Tukey test for multiple comparisons. Control chow-diet (Con), high-fat diet (HFD), negative control (NC) GapmeR (HFD/NC) and GapmeR targeting lncMGC (HFD/Gap). n=4-5. The bar and whisker plot displays the distribution of the data. The whiskers extend from the minimum to the maximum values. Individual data points are overlaid as dots. Statistically significant p-values are indicated in the bar graphs.

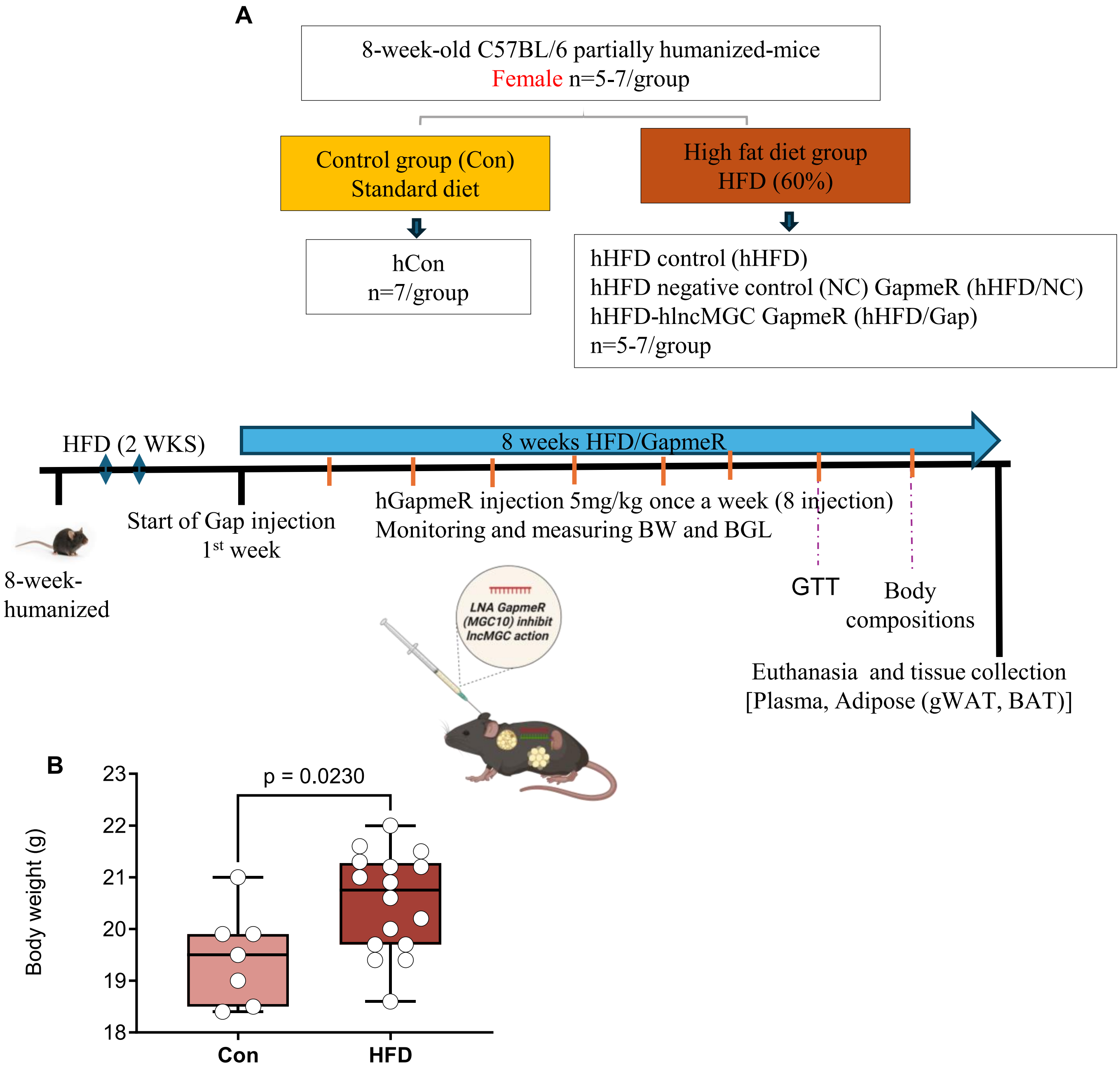

Figure S16. Experimental scheme for treatment with GapmeR targeting human lncMGC in high-fat diet (HFD) fed induced- obesity mice model with partially humanized lncMGC mice. (A) 8-wk-old female partially humanized lncMGC (hlncMGC) mice were randomly divided into groups and fed with laboratory control chow-diet (hCon) or high-fat diet (hHFD) (60% kcal from fat) for 2 weeks, then, hHFD mice were randomly divided into 3 groups and injected with negative control (NC) GapmeR (hHFD/NC) or GapmeR targeting lncMGC (hHFD/Gap) at 5 mg/kg body weight once a week for 8 weeks. hHFD-fed mice without treatment were used as control (hHFD). Body weight (BW) and blood glucose levels (BGL) were monitored weekly during 10 weeks of diets. Glucose tolerance tests (GTT) were performed, and body composition was measured at 10 weeks of study. After euthanasia, plasma, perigonadal white adipose tissue (gWAT), and interscapular brown adipose tissue (BAT) were harvested. (B) Average body weights after the first 2 weeks of HFD. Statistical comparisons between two groups were performed using two-sided Student's t-test. The whiskers extend from the minimum to the maximum values. Individual data points are overlaid as dots. P-values are indicated in the bar graphs.

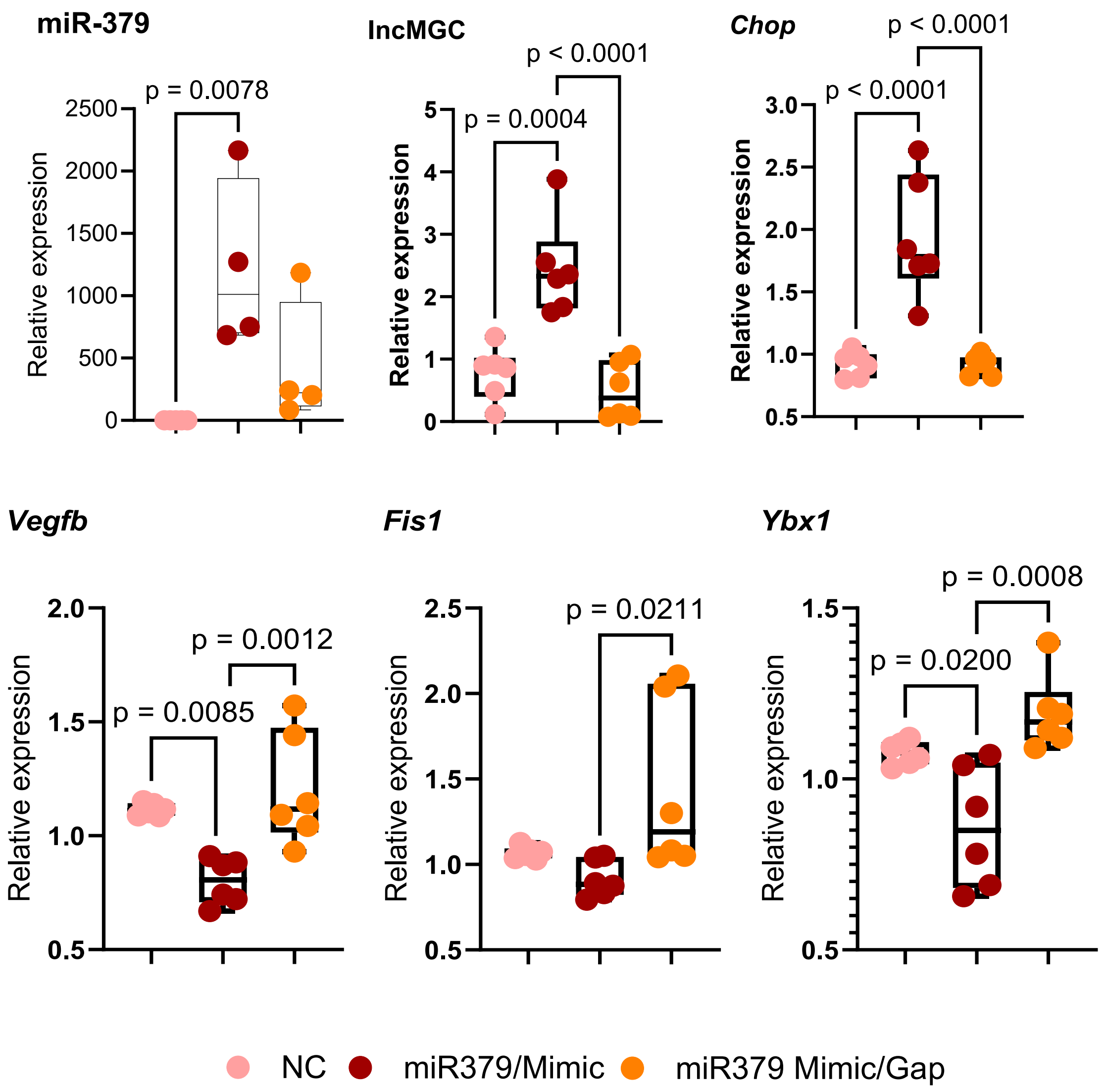

Figure S17. HIB1B brown adipocytes (~3.5 × 10⁵ cells/transfection) were transfected with oligo mimics of miR-379 (50 nmol/L) or the corresponding negative control (NC) oligos (50 nmol/L) using Lipofectamine RNAiMAX. Cells were then treated with lncMGC-GapmeR (2 µmol/L) for 3 days via Gymnosis. Expression of miR-379, lncMGC , *Chop,* and the miR-379 target genes *Vegfb*, *Fis-1,* and *Ybx1*. Statistical analyses were performed by one-way ANOVA with a post-hoc Tukey test for multiple comparisons. n=6.The bar and whisker plot displays the distribution of the data. The whiskers extend from the minimum to the maximum values. Individual data points are overlaid as dots. Statistically significant p-values are indicated in the bar graphs.

Table S1. Clinical parameters of lean, overweight /obese human donors of adipose tissues.

Individual ID		HbA1c %	BMI
Lean	N1	4.4	23.63
	N2	5.1	23.8
	N3	5.3	22.79
	N4	5.2	22
	N5	5.1	20.7
overweight/obese	N1	4.6	36
	N2	5.6	32
	N3	5.3	33
	N4	5.8	29.9
	N5	5.7	26.8
	N6	4.9	25.2
	N7	5.9	31.3
	N8	5.7	32.3
	N9	5.4	33
	N10	4.5	36

The fully de-identified white adipose tissue (WAT) samples were obtained as discard tissues from the Southern California Islet Cell Resource Center (City of Hope) and classified into two groups—lean, and overweight/obese based on the donor's body mass index (BMI).

Table S2. The sequences of PCR primers used in this study.

Target	Forward primer	Reverse primer
mVegfb	GAACCTCATGTCTCACCTCAG	TCATAACAGAACCCAAATCCCG
mTfam	AGC GTG CTA AAA GCA CTG GG	ACT TCG GAA TAC AGA CAA GAC TGA
mFis1	ACCATCGCCTTCCCTTTTC	ATGACAGGGTTAAACGACAGG
mYbx1	GCA GAC CGT AAC CAT TAT AGA CG	TCT CCG CAT GTA GTA AGG TGG
mChop (Ddit)	GCACCTATATCTCATCCCCAG	TGCGTGTGACCTCTGTTG
mPpargc1a (Pgc1)	CACCAAACCCACAGAAAACAG	GGGTCAGAGGAAGAGATAAAGTTG
mPrkn	GCA CAC CCA ACC TCA GAC AA	TCA GTG GAG ATG AGG CCG A
mPink1	GTG GGA CTC AGA TGG CTG TC	ACT GGA GCT GTT GAA AGG CAG
mCebpb	AGCCCCTACCTGGAGCCGCTCGCG	GCGCAGGGCGAACGGGAAACCG
mPparg	TGTTATGGGTGAAACTCTGGG	AGAGCTGATTCCGAAGTTGG
mlncMGC	ATTTTTCTGAGTTAGTGTGGCCTTCATCTG	TCAGGAACCATGGAACGGTGTTGACCCCTAG
mCypa	ATGGTCAACCCCACCGTGT	TTCTTGCTGTCTTTGGAACTTTGTC
hlncMGC	GCCTGCTTCCAATGCCAAATC	CTTCAGGAACCACGGAATGGT
hCYPA	CCCACCGTGTTCTTCGACATT	GGACCCGTATGCTTTAGGATGA

Table S3: **Sample ID, ROI selected area, and nuclei count per ROI**.

Sample_ID	ROI	Segment	Area	Nuclei	(v1.0) Mouse NGS Whole Transcriptome Atlas RNA
WT Con	001	Full ROI	97592.29	290	MW0031123
WT Con	002	Full ROI	97592.29	392	MW0031123
WT Con	003	Full ROI	97592.29	221	MW0031123
WT Con	004	Full ROI	97592.29	217	MW0031123
WT HFD	005	Full ROI	97592.29	129	MW0031123
WT HFD	006	Full ROI	97592.29	168	MW0031123
WT HFD	007	Full ROI	97592.29	174	MW0031123
WT HFD	008	Full ROI	97592.29	158	MW0031123
WT HFD	009	Full ROI	97592.29	206	MW0031123
WT HFD	010	Full ROI	97592.29	170	MW0031123
lncMGC KO Con	011	Full ROI	97592.29	323	MW0031123
lncMGC KO Con	012	Full ROI	97592.29	360	MW0031123
lncMGC KO Con	013	Full ROI	97592.29	345	MW0031123
lncMGC KO Con	014	Full ROI	97592.29	330	MW0031123
lncMGC KO Con	015	Full ROI	97592.29	365	MW0031123
lncMGC KO HFD	016	Full ROI	97592.29	223	MW0031123
lncMGC KO HFD	017	Full ROI	97592.29	274	MW0031123
lncMGC KO HFD	018	Full ROI	97592.29	258	MW0031123
lncMGC KO HFD	019	Full ROI	97592.29	282	MW0031123
lncMGC KO HFD	020	Full ROI	97592.29	263	MW0031123
lncMGC KO HFD	021	Full ROI	97592.29	311	MW0031123
lncMGC KO HFD	022	Full ROI	97592.29	269	MW0031123

Sample ID, ROI selected area, and nuclei count per ROI. from brown adipose tissues in wild type control (WT Con), WT-high fat diet (HFD), lncMGCKO Con and lncMGCKO HFD female mice. ROI: Region of interest

Table S4: **ER-stress related genes.**

Gene ID	Log2FC WT HFD vs WT Con	P value	Log2FC KO HFD vs KO Con	P value
Hspa1a	1.3299	0.0086	0.6337	0.6105
App	0.7965	0.0063	0.4292	0.0001
Ccnd1	0.5279	0.0139	0.7302	0.0003
Nck1	0.4272	0.0137	0.3314	0.0095
Bcl2l1	0.3983	0.0023	0.2974	0.0727
Trim25	0.2849	0.0493	0.1361	0.1540
Hsp90b1	0.2661	0.0296	-0.1852	0.0325
Ddit3	0.2602	0.0566	-0.0335	0.8923
Ube4b	0.2532	0.0728	-0.0013	0.9915
Ppp2cb	0.2467	0.0696	0.0786	0.4564
Serinc3	0.2446	0.0258	0.1418	0.1631
Sgf29	0.2434	0.0445	0.1445	0.2667
Ubxn4	0.2127	0.0805	-0.1772	0.4392
Hspa5	0.2038	0.0277	-0.1657	0.1423

Differential expression of ER stress–related genes in brown adipose tissue from WT and lncMGC-KO mice. Using RNA-seq analysis, genes associated with endoplasmic reticulum (ER) stress were upregulated in WT-HFD mice compared with controls. Expression of these ER stress–related genes was attenuated in lncMGC KO HFD mice relative to lncMGC KO controls. The table summarizes fold-change (FC) values for ER stress–related genes.
