# Supplementary material for: Metabolic beneficial effects of targeting a long non-coding RNA, lnc-megacluster, in obesity
Source: Mol Ther Nucleic Acids. 2025 Dec 8;37(1):102792. doi: 10.1016/j.omtn.2025.102792 (PMC12794071; doi:10.1016/j.omtn.2025.102792)
Supplement: Document S2. Article plus supplemental information [file mmc2.pdf]

Molecular Therapy
Nucleic Acids
Original Article

Metabolic beneficial effects of targeting a long non-coding RNA, lnc-megacluster, in obesity

Maryam Abdollahi,[1] Vajir Malek,[1] Vinay Singh Tanwar,[1] Mitsuo Kato,[1] Linda Lanting,[1] Alireza Rezaei,[2] Lingxiao Zhang,[1] Lixin Yang,[2] Raju K. Pillai,[2,3] Leah Kebrom,[1] Jathan Nandi,[1] Wendong Huang,[1,4] Ke Ma,[1,4] and Rama Natarajan[1,4]

[1]Department of Diabetes Complications and Metabolism, Arthur Riggs Diabetes and Metabolism Research Institute and Beckman Research Institute of City of Hope, Duarte, CA 91010, USA; [2]Research Molecular Pathology Shared Resource, Beckman Research Institute of City of Hope, Duarte, CA 91010, USA; [3]Department of Pathology, City of Hope National Medical Center, Duarte, CA 91010, USA; [4]Irell and Manella Graduate School of Biological Sciences, Beckman Research Institute of City of Hope, Duarte, CA 91010, USA

The long noncoding RNA (lncRNA) lnc-megacluster (lncMGC) is implicated in diabetic kidney disease and pancreatic islet dysfunction. However, its role in obesity and insulin resistance (IR) is unknown. Herein, we investigated the regulatory role of lncMGC in obesity and adipose dysfunction using lncMGC knockout-(KO) mice and further determined the translational potential of lncMGC-based therapeutics for obesity using GapmeR antisense oligonucleotides in wild-type and partially humanized-lncMGC mice. We found lncMGC is upregulated in perigonadal white adipose (gWAT) and brown adipose tissues (BAT) from high-fat diet (HFD)-induced obese mice along with increased endoplasmic reticulum stress signaling. Inhibition of lncMGC in mice via genetic ablation or GapmeRs targeting mouse or human lncMGC displayed protective effects against HFD-induced IR, weight gain, and associated adipose dysfunction, with some sex-specific differences. In parallel, key lncMGC targets regulating gWAT and BAT functions were altered. In gWAT, loss of lncMGC either in KO mice or through GapmeR treatment improved angiogenesis and reduced adipocyte hypertrophy and inflammation. In BAT, lncMGC deficiency or inhibition enhanced mitochondrial thermogenesis and mitophagy markers. Collectively, these new findings underscore the pathogenic role of lncMGC in adipose dysfunction and the therapeutic potential of targeting key lncRNAs for obesity and associated metabolic dysfunction.

INTRODUCTION

The prevalence of obesity has reached epidemic proportions worldwide, posing a major public health challenge,[1,2] because obesity, a chronic inflammatory condition, greatly increases the risk for type 2 diabetes (T2D) and associated comorbidities like kidney, liver, and cardiovascular diseases.[2–4] The number of overweight adults is estimated to increase significantly, with over 40% of adults expected to be obese in the U.S. by 2030[5,6] and hence effective interventions are needed to counteract this trend. Currently, lifestyle and dietary modifications, along with therapeutic treatment including glucagon-like peptide receptor-1 agonists (GLP1-RAs), have been highly effective in managing obesity.[7] However, these drugs can also have adverse outcomes, including body weight regain after discontinuation of medication, weight loss accompanied by reduction in muscle mass, and high costs.[8,9] There is thus an urgent need to increase efforts in identifying additional factors driving obesity and target them using mono- or combination therapies to prevent obesity.[10]

Over 90 percent of the human genome encodes for RNAs that are not translated into proteins, known as noncoding RNAs (ncRNAs), including long noncoding RNAs (lncRNAs, greater than 200 nucleotides), microRNAs (miRNAs), and tRNA-derived and rRNA-derived small RNAs. Since their discovery, the biological relevance of ncRNAs has become increasingly apparent, including their involvement in genome organization and regulation of gene expression through various mechanisms, such as post-translational modifications and epigenetic regulation.[11] Besides, ncRNAs are increasingly recognized as crucial contributors to various diseases, including obesity.[12,13] miRNAs regulate gene expression via interaction with their target genes to repress their translation or induce their degradation, thereby altering cellular states and disease conditions regulated by these target genes. LncRNAs, on the other hand, can have several mechanisms of action, which also depend on their subcellular location. Some lncRNAs can serve as host genes for miRNAs, or as sponges for miRNAs, thereby preventing interactions with the corresponding miRNA target

Received 8 April 2025; accepted 3 December 2025;
https://doi.org/10.1016/j.omtn.2025.102792.

Correspondence: Maryam Abdollahi, Department of Diabetes Complications and Metabolism, Arthur Riggs Diabetes and Metabolism Research Institute and Beckman Research Institute of City of Hope, Duarte, CA 91010, USA.
E-mail: mabdollahi@coh.org
Correspondence: Rama Natarajan, Department of Diabetes Complications and Metabolism, Arthur Riggs Diabetes and Metabolism Research Institute and Beckman Research Institute of City of Hope, Duarte, CA 91010, USA.
E-mail: rnatarajan@coh.org

© 2025 The Author(s).
Published by Elsevier Inc. on behalf of The American Society of Gene and Cell Therapy.
This is an open access article under the CC BY license (http://creativecommons.org/licenses/by/4.0/).

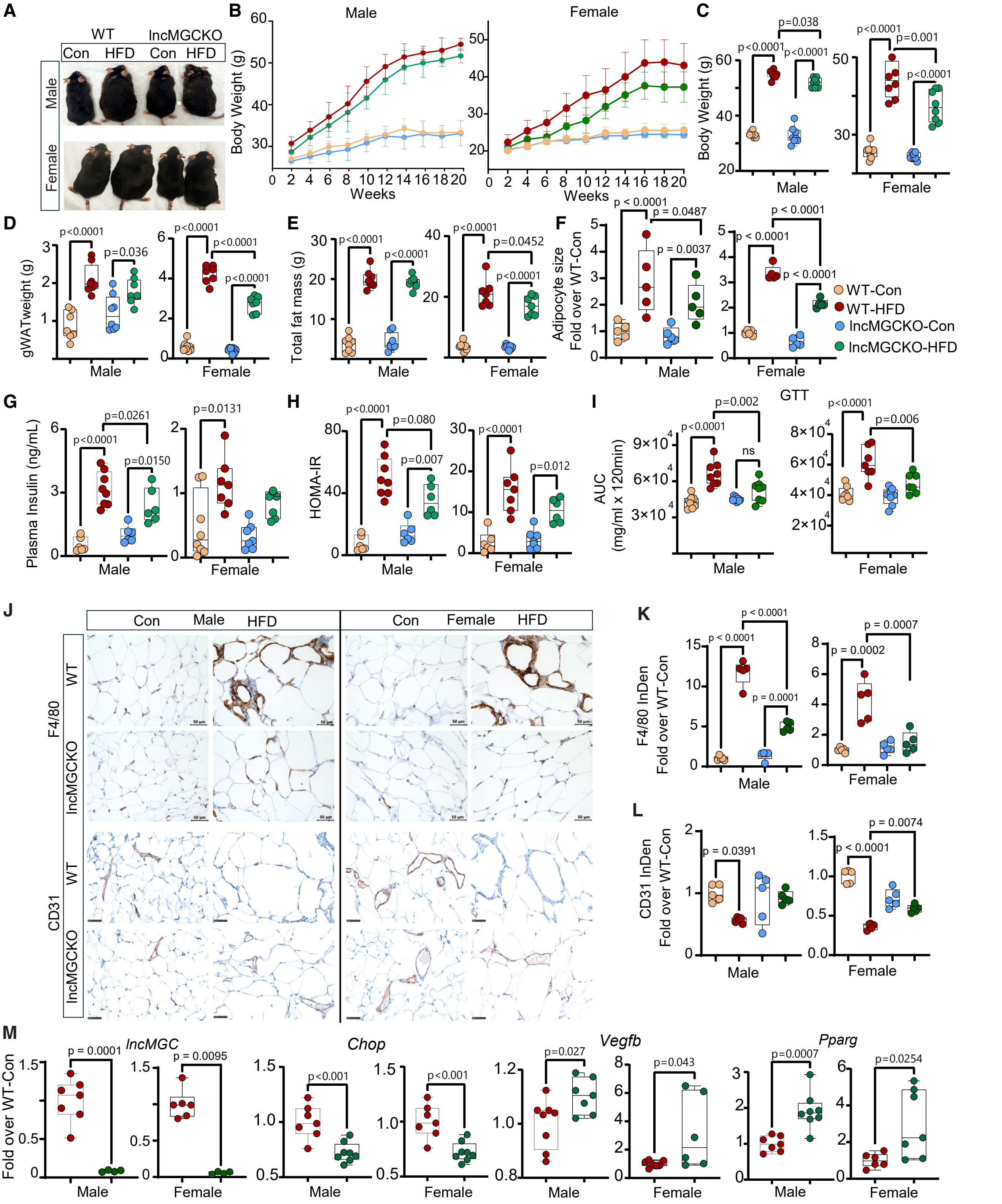

(legend on next page)

genes.[14,15] LncRNAs are emerging as promising therapeutic targets and biomarkers due to their roles in regulating cellular processes and disease progression.

Using various molecular techniques in combination with genetic mouse models, our previous studies revealed the functional involvement of several key miRNAs and lncRNAs in metabolic disorders, including vascular dysfunction, hypertension, inflammation, diabetic kidney disease, obesity, and insulin resistance (IR).[14,16–28] The human lnc-megacluster (lncMGC) lncRNA, hosting about 40 miRNAs (miR-379 cluster), is located on human Chromosome 14q32.2 (chr14). In mice, the lncMGC is situated on chromosome 12qF1 (chr12) and includes the miR-379 cluster from miR-379 to miR-3072.[20,27,29] We demonstrated that miR-379 and its host transcript, lncMGC, are induced by endoplasmic reticulum (ER) stress in the pancreas of diabetic mice and isolated human pancreatic islets from diabetic subjects.[29] A GapmeR (locked nucleic acid [LNA] and phosphorothioate-modified antisense oligonucleotide [ASO]) targeting lncMGC could ameliorate hyperglycemia in mice models of type 1 diabetes and also preserve human and mouse islet viability.[29] The lncMGC/miR-379 axis is increased in the kidney glomeruli of diabetic[18] and high-fat diet (HFD) fed mice,[30] and miR-379 has been implicated in obesity and IR in mice through modulation of key target genes.[31] The functional roles of several miRNAs in obesity and related disorders have been reported to involve adipocyte differentiation, fat metabolism, or insulin signaling.[32–34] However, the specific role of lncMGC in obesity and adipose tissue dysfunction has not been examined. Moreover, the translational potential of lncRNA-based therapies for adipose dysfunction associated with IR and obesity has not been well investigated. Here, using novel lncMGC knockout (lncMGC KO) mice created by CRISPR-Cas9 editing, we show that HFD upregulates adipose lncMGC, and that lncMGC alters the expression of target factors involved in adipogenesis and mitochondrial function in adipose tissues, thereby contributing to the pathogenesis of obesity, adipose dysfunction, IR, and metabolic disease. In addition, using GapmeRs targeting mouse and human lncMGC, we show that targeting lncMGC may be a novel therapeutic approach to combat these disorders.

RESULTS

HFD-induced obesity, adipocyte hypertrophy, and impaired insulin sensitivity are attenuated in lncMGC KO mice

LncMGC KO mice were generated by CRISPR-Cas9 editing approach as described earlier[27,29] and the lncMGC KO5 line was used in the current study. To determine the regulatory role of lncMGC in obesity-associated adipose tissue dysfunction, wild-type (WT) and lncMGC KO male and female mice were fed with a 60% HFD for 20 weeks, while keeping mice fed with a normal chow diet as controls (Con) (Figure S1A). All HFD-fed mice gained significant body weight (Figures 1A–1C and S1B), perigonadal white adipose tissue (gWAT), and total fat mass compared to their respective controls (Figures 1D and 1E). Interestingly, lncMGC KO HFD mice (male and female) showed significantly lower body weight along with comparatively lower gWAT and total fat mass than WT HFD mice, and these differences were more evident in female mice (Figures 1A–1E and S1B). However, lean mass, body temperature, plasma total cholesterol (TC), and triglyceride levels of WT HFD and lncMGC KO HFD mice were comparable (Figures S2B–S2D). Consistent with the lower body weight observed in lncMGC KO HFD mice, gWAT expansion and adipocyte hypertrophy seen in WT HFD mice were significantly attenuated in male and female lncMGC KO mice on HFD (Figure 1F).

WT HFD mice showed moderate increase in blood glucose levels (BGLs, Figure S2A), along with significant insulin resistance characterized by hyperinsulinemia (Figure 1G), and as assessed by HOMA-IR (an insulin resistance index, Figure 1H), and glucose tolerance tests (GTT, Figure 1I) as compared to WT Con mice. Interestingly, male lncMGC KO HFD mice display improvement in these indices, indicating better insulin sensitivity when compared to male WT HFD mice. On the other hand, female lncMGC KO HFD mice showed improved glucose tolerance but no change in plasma insulin and HOMA-IR, versus female WT HFD mice. In addition, HOMA-B%, an index of beta cell function,[35,36] which had a significant correlation with plasma insulin levels, was increased in WT HFD mice (Figures S3A and S3B), whereas no change was observed in lncMGC KO HFD mice when compared to respective control mice (Figure S3A). Hematoxylin and eosin (H&E) staining revealed pancreatic islet hyperplasia in WT HFD, which was alleviated in lncMGC KO HFD mice (Figure S3C), suggesting potential protective effects against HFD-induced beta cell dysfunction in these KO mice. We compared lncMGC KO and WT mice under baseline (control diet) conditions and did not observe any significant differences in general characteristics or phenotype, including body weight, GTT, BGLs, or key physiological markers such as insulin levels. These findings suggest that the observed effects under HFD conditions are not due to baseline differences in the knockout mice.

Figure 1. High-fat diet (HFD)-induced obesity and impaired insulin sensitivity are attenuated in lncMGC KO mice

(A) Representative images of wild type (WT) and lncMGC KO control (Con) and HFD-fed male and female mice after 20 weeks on the HFD. (B–D) Biweekly body weight gain, (C) endpoint body weight, (D) gWAT weight, (E) total fat mass, (n = 7–8 mice/group), and (F) adipocyte size (5/group), in male and female mice. (G–I) plasma insulin levels, (H) HOMA-IR, and (I) area under the curve (AUC) for GTT, in male and female mice. (J–L) IHC staining of F4/80 (macrophage inflammation marker) and CD31 (endothelial cell marker) in gWAT sections from WT and lncMGC KO male and female mice. Scale bars, 50 μm. Quantitative analysis of (K) F4/80 (*n* = 5/group) and (L) CD31 (*n* = 5/group) in male and female mice. (M) Gene expression of *lncMGC (n = 4-7,* lncMGC was not detected by qPCR in 4 samples in lncMGC KO HFD mice*), Chop*, *Vegfb*, and *Pparg* in isolated stromal vascular fraction (SVF) from gWAT in male and female mice (n = 7–8/group). Statistical analyses were performed by two-way ANOVA with post-hoc Tukey test for multiple comparisons. Student's *t* tests for comparisons between two groups. XY graphs show the mean (SD). The bar and whisker plot displays the distribution of the data. The whiskers extend from the minimum to the maximum values. Individual data points are overlaid as dots. Statistically significant *p* values are indicated in the bar graphs.

HFD-induced impaired angiogenesis, adipogenesis, and ER-stress are attenuated in lncMGC KO mice

Appropriate angiogenesis helps maintain adipose tissue function, whereas impaired angiogenesis in an obese state may induce hypoxia, exacerbate inflammation, and lead to metabolic dysfunction.[37] Herein, we examined whether lncMGC KO can improve these pathways in the gWAT of HFD mice. In comparison with the male and female WT HFD cohort, lncMGC KO HFD mice displayed markedly lower gWAT inflammation, along with improved angiogenesis, as indicated by F4/80 (a macrophage marker) and CD31 (an endothelial marker) immunostaining, respectively (Figures 1J–1L).

The stromal vascular fraction (SVF) isolated from adipose tissue represents heterogeneous cell populations composed of preadipocytes (a precursor of mature adipocytes), endothelial cells (integral for angiogenesis), and various progenitors (involved in adipose tissue remodeling).[38,39] To examine the potential mechanisms/factors mediating the involvement of lncMGC in adipocyte hypertrophy and gWAT dysfunction, we isolated SVF from gWAT of WT and lncMGC KO mice. lncMGC expression was significantly increased in SVF from WT HFD mice (male and female) compared to chow diet controls (Figure S2E). As expected, lncMGC was absent in SVF from lncMGC KO HFD mice (Figure 1M). The expression of C/EBP homologous protein (*Chop*), pro-endoplasmic reticulum [ER] stress factor, and regulator of lncMGC expression[20] was significantly decreased in parallel. In contrast, vascular endothelial growth factor B (*Vegfb*), a proangiogenic factor and target of miR-379, and peroxisome proliferator-activated receptor γ (*Pparg*), adipogenic marker, were upregulated in SVF from lncMGC KO HFD mice compared to WT HFD male and female mice (Figure 1M). These findings suggest that lncMGC KO may attenuate HFD-induced inflammation and ER stress, and potentially enhance angiogenesis and adipogenesis, although further studies are needed to confirm these effects.

Energy expenditure and oxygen consumption are increased in lncMGC KO mice

Whole-body energy homeostasis was monitored to explore possible mechanisms by which lncMGC KO protects mice against HFD-induced weight gain and adipose tissue dysfunction. These analyses include food intake, movement, energy expenditure (EE), oxygen consumption (VO2), and energy balance.[31] EE was greater in male lncMGC KO HFD mice as compared to WT HFD mice (Figures 2A and 2C), while in females, EE was increased only in lncMGC KO mice under chow diet (Con) condition as determined by ANOVA using total mass as covariate (Figures 2B and 2D). Similarly, oxygen consumption (VO2) was also increased in lncMGC KO mice (Figure S4). Total calorie intake from food, measured in kilocalories using metabolic cages, was increased during the nighttime in WT and lncMGCKO HFD compared to their respective control mice (Figure 2E). However, food and water intake, locomotor activity, and respiratory exchange ratio (RER) were comparable between the genotypes (Figures 2E–2G), indicating that lncMGC KO did not alter feeding and activity patterns. Interestingly, HFD-induced increased energy balance observed in WT male mice was nearly abolished in lncMGC KO HFD mice (Figure 2H). Together, these data suggest that increased EE in the KO mice may be potentially attributed to the improvement in brown adipose tissue (BAT) function, as BAT is closely associated with energy production.

Factors related to energy metabolism and mitochondrial activity in BAT are improved in lncMGC KO HFD female mice

Recent advances in spatial transcriptomics have made it possible to perform high-throughput quantification of RNAs within specific regions of intact tissue sections, thereby enabling precise measurement of gene expression *in situ* at cellular resolution.[40] BAT is integral to EE, primarily by generating heat through thermogenesis, which significantly influences lipid metabolism and plays a vital role in maintaining the body's overall energy homeostasis.[41] Therefore, to elucidate the mechanisms underlying our observation of improved EE in lncMGC KO mice, we generated a whole transcriptome atlas (WTA) using GeoMx digital spatial profiling (DSP) platform in BAT from WT and lncMGC KO female mice. Subsequently, we performed RNA sequencing analysis (Figure S5, as described in the methods section. Volcano plots represent the differentially expressed genes (DEGs) between WT HFD versus WT Con, lncMGC KO HFD versus lncMGC KO Con, and lncMGC KO HFD versus WT HFD female mice, respectively (Figures 3A–3C). Interestingly, lncMGC KO HFD mice showed upregulation of mitochondrial function (e.g., *Ucp-1* and *Cox15*) and lipid metabolism [very-long-chain fatty acid elongase (*Elovl3*)][42] related genes in comparison to WT HFD mice (Figure 3C). Gene ontology biological process (GOBP) analysis of the downregulated genes revealed strong enrichment of genes (gene numbers based on their associated biological processes) involved in the generation of precursor metabolites and energy and fatty acid metabolic processes in the WT HFD, and fatty acid metabolic process in lncMGC KO HFD mice compared to their respective controls (Figures 3D and 3E). Notably, these pathways were significantly improved in lncMGC KO HFD, as assessed by adjusted *p* values for the enrichment of these pathways (Figure 3F). As illustrated in the heatmap in Figure 3G, key mitochondrion-related genes were markedly decreased under HFD as compared to the chow diet in the WT mice. However, these reductions were attenuated in lncMGC KO HFD mice. These include Mdh1 (malate dehydrogenase 1), which is involved in cellular metabolism, or *Cox7c* and *b*, subunits of cytochrome *c* oxidase (Complex IV) in the mitochondrial electron transport chain, which plays a pivotal role in the final step of oxidative phosphorylation.[43] Additionally, Insulin receptor substrates 1 and 2 *(Irs1* and *Irs2*), which are critical factors in the insulin signaling pathway and influence glucose uptake, lipid metabolism, and thermogenesis in BAT,[44] were markedly downregulated in WT HFD but improved in lncMGC KO HFD compared to WT HFD mice. However, no significant changes were observed in differential gene expression (Figure S6A) or GOBP (Figures S6B andS6C) when comparing basal levels between WT and lncMGC KO control groups, indicating the results observed in lncMGC KO HFD mice are likely due to the reduction of lncMGC under HFD conditions.

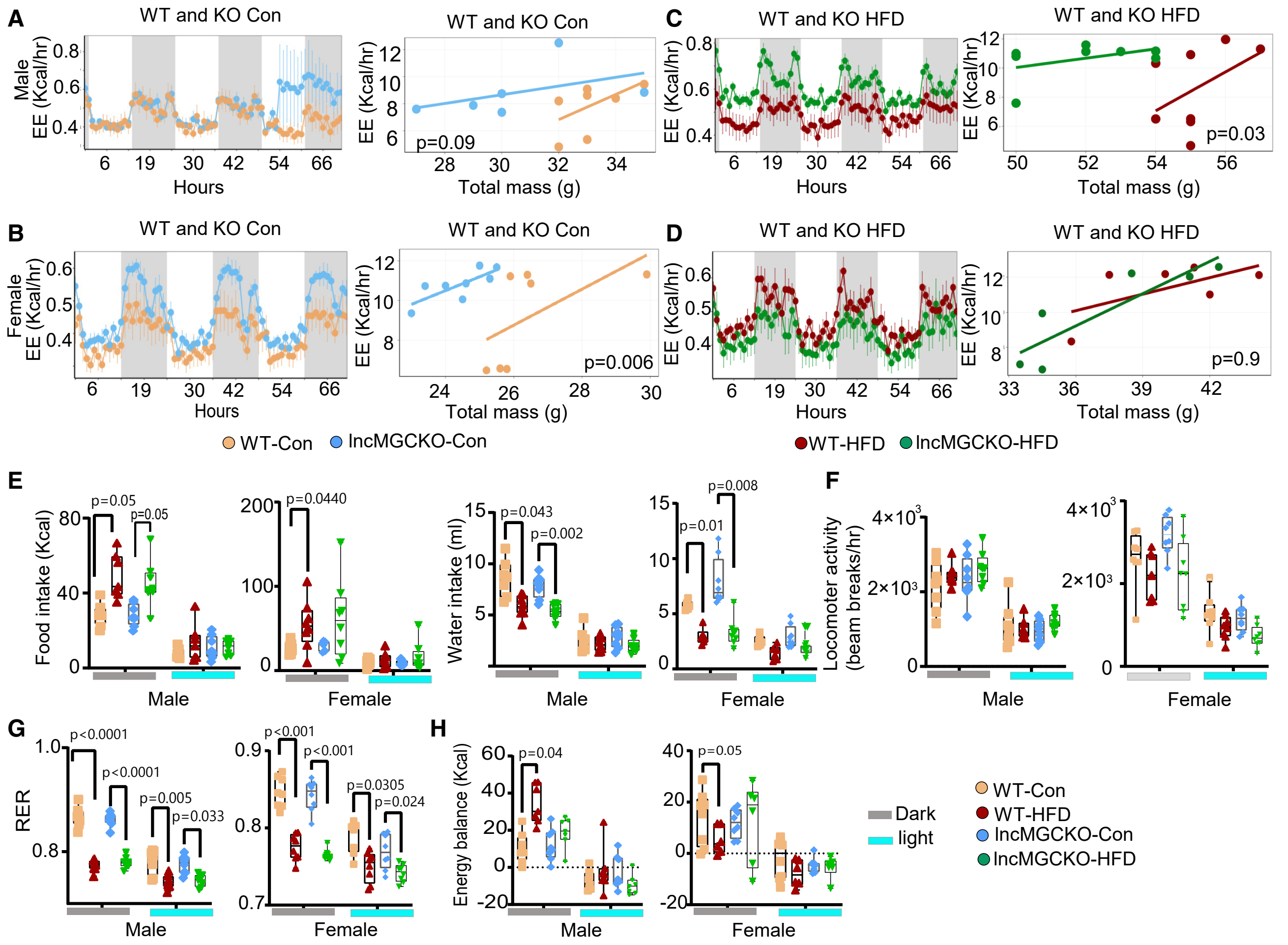

Figure 2. lncMGC KO mice exhibited higher energy expenditure compared to their respective controls

Energy expenditure (EE) and regression analysis of EE and total body mass in (A and B) WT and lncMGC KO Control and in (C and D) WT and lncMGC KO HFD male and female mice during day and night (gray-shades) time. Metabolic parameters, including (E) food and water intake, (F) movement, (G) respiratory exchange ratio (RER), and (H) energy balance in male and female mice during day and night-time. Data were analyzed for EE and total mass regression using ANCOVA with EE as a dependent variable, genotype as fixed variable, and body mass as a covariate. two-way ANOVA with post hoc Tukey's test for multiple comparisons was used to analyze metabolic parameters. The bar and whisker plot displays the distribution of the data. The whiskers extend from the minimum to the maximum values. Individual data points are overlaid as dots. Statistically significant *p* values are indicated in the bar graphs.

Factors related to ER-stress in BAT are improved in lncMGC KO HFD female mice

Further analysis of RNA-seq data revealed a significant enrichment of genes related to ER stress in the WT-HFD group. These ER stress-associated genes were markedly upregulated in WT-HFD, indicating activation of the unfolded protein response. Notably, these ER stress-related genes were attenuated in the lncMGCKO-HFD group, suggesting a potential protective effect that may contribute to improved BAT function (Figure S7). ER dysfunction is not limited to the endoplasmic reticulum but also impacts other components of the protein secretory pathway, as evidenced by the upregulation of some protein chaperones, including heat shock proteins (HSPs).[45,46] For instance, ER stress-related transcript HSPA5 is elevated in individuals with higher body mass index.[47] In our RNA-seq analysis, we found HSP genes such as *Hsp90b1*, *Hspa5*, *Hspa1a* were increased in WT-HFD and reduced in lncMGCKO HFD mice. Notably, the increased expression of *Ddit3/Chop*, ER stress marker and regulator of lncMGC expression, observed in WT-HFD mice vs. control mice, decreased in lncMGCKO HFD mice (Figure S7). It is known that cyclin D1 (*Ccnd1*) plays a key role in regulating PPARγ-driven adipocyte differentiation by increasing histone deacetylase activity, thereby inhibiting both PPARγ activity and adipogenesis.[48] Furthermore, studies have shown that tripartite motif-containing 25 (TRIM25) regulates adipocyte differentiation through proteasome-mediated degradation of PPARγ, and that knockdown of TRIM25 increases PPARγ protein levels and enhances adipogenic differentiation.[49] Our RNA-seq data show that the increased expression of these genes in WT-HFD mice was

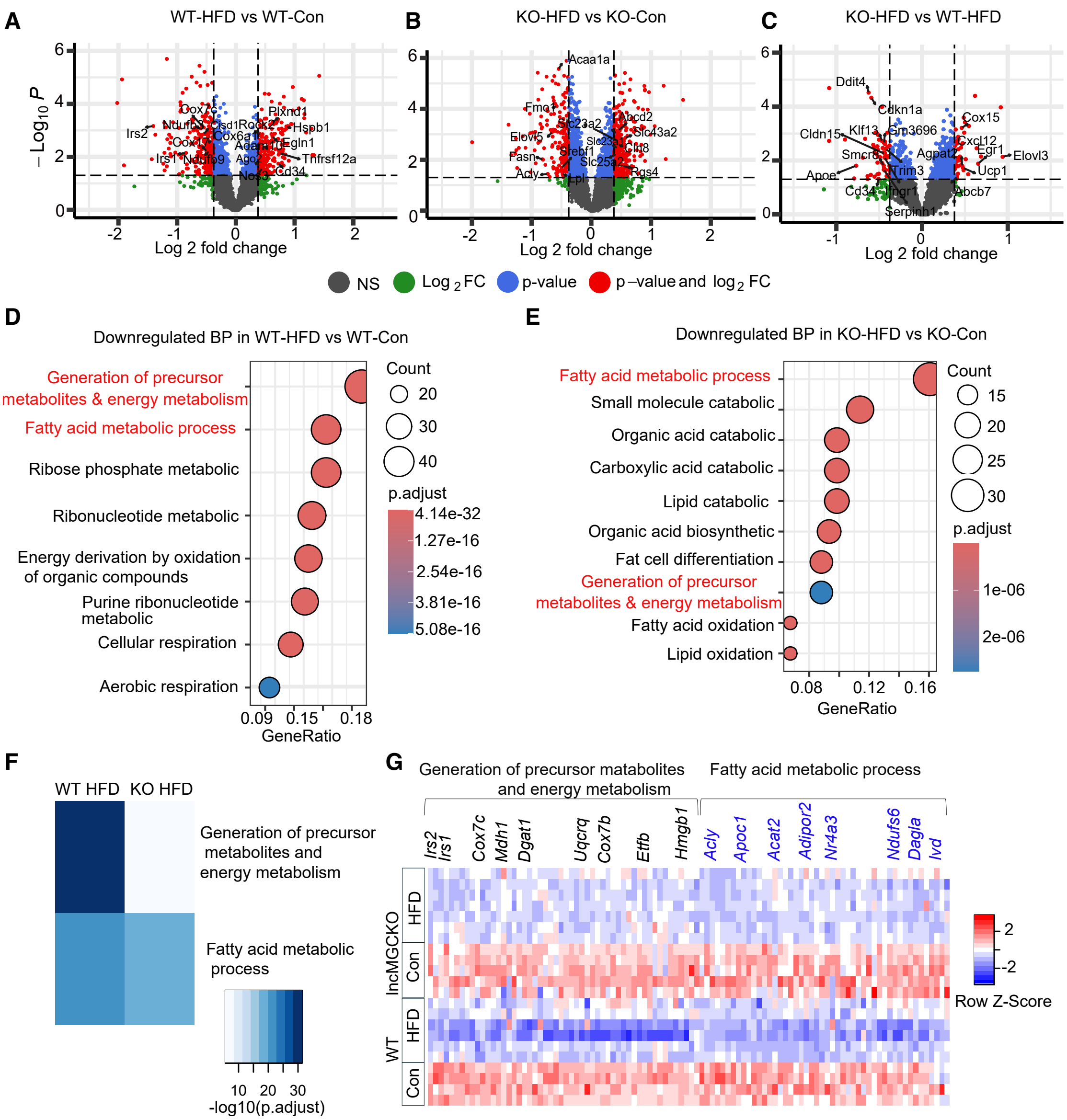

Figure 3. Factors related to energy metabolism and mitochondrial activity are improved in brown adipose tissue of lncMGC KO HFD female mice

The Nanostring GeoMx Digital Spatial Profiler (DSP) Whole Transcriptome Atlas (WTA) was used to analyze multiple RNA analytes from a single paraffin-embedded BAT sample. Volcano plots obtained from RNA sequencing analysis represent comparison of differential gene expression between (A) WT HFD versus WT Con, (B) lncMGC KO HFD versus lncMGC KO Con, and (C) lncMGC KO HFD versus WT HFD mice. Top enriched downregulated gene ontology biological process (GOBP), gene numbers based on their associated biological processes, in (D) WT HFD vs. WT Con and (E) lncMGC KO HFD vs. lncMGC KO Con and (F) comparison of enriched biological process of WT HFD vs. WT Con and lncMGC KO HFD vs. lncMGC KO Con. (G) A heatmap illustrating the genes related to generation of precursor metabolites and energy and fatty acid metabolic process were significantly modulated between regions of interest (ROIs) in UCP-1 positive segments across all groups; normalized Q3 values used to generate the heatmap. Data analysis was performed using R. Log2 fold change ±0.378 and p value <0.05. The columns of the heatmap represent mitochondrion-related genes depicting markedly altered expression levels as indicated by fold change.

downregulated in lncMGCKO HFD mice (Figure S7). We compared the fold changes (FC) in the expression of ER stress-related genes across groups. This analysis revealed that lncMGC KO decreased the HFD-induced upregulation of ER stress-related genes, indicating an attenuation of ER stress signaling in lncMGC KO HFD mice compared with WT-HFD mice (Table S4).

Collectively, these findings also suggest that enhanced BAT function observed in lncMGCKO HFD mice may be mediated, at least in part, by improved ER stress responses and enhanced adipogenesis.

To strengthen the mechanistic aspects, we also conducted a more in-depth pathway analysis of the RNA-seq data, which revealed strong

enrichment in pathways related to protein regulation, including protein catabolic processes, localization, as well as transport (e.g., carboxylic acid, organic acid, organic anion, and organic amine), and adipogenesis, also evidenced by increased fat cell differentiation in lncMGC KO HFD vs. WT HFD mice (Figure S8).

Together, these data suggest that increased EE in lncMGC KO mice is due to increased mitochondrial activity, adipogenesis, and related processes in BAT.

Loss of lncMGC improves mitochondrial structure and function in brown adipose tissue in HFD-fed mice

The data from DSP noted previously revealed that metabolic improvements in BAT of the lncMGC KO mice could be driving the anti-obesity effect, at least in part. We thus examined the regulatory impact of lncMGC on BAT structure and function through analysis of BAT whitening and mitochondrial morphological changes. Increased whitening of BAT and lipid droplets in WT HFD mice (versus WT Con) were significantly attenuated in lncMGC KO HFD, in both male (Figures 4A and 4B) and female (Figures 4F and 4G) mice. Transmission electron microscopy (TEM) analysis of mitochondrial ultrastructure revealed that abnormal morphology of BAT mitochondria characterized by elongated shape and loss of structured cristae (outlined in red) seen in WT HFD mice were markedly alleviated in lncMGC KO HFD male (Figure 4A) and female (Figure 4F) mice. Furthermore, decreased mitochondria number and increased mitochondrial area and length detected in WT HFD mice were not observed in lncMGC KO HFD male (Figure 4C) and female mice (Figure 4H). Immunostaining revealed that HFD-induced decrease in the expression of uncoupling protein-1 (UCP-1), a key thermogenic marker, mitochondrial fission 1 protein [(FIS1), a mitophagy marker and target of miR-379],[18] and RNA binding protein Y-box binding protein 1 [(YBX1),[50] a potential target of miR-379] in WT HFD mice were attenuated in lncMGC KO HFD male (Figure 4D) and female (Figure 4I) mice. Additionally, increased expression of CHOP protein staining observed in WT-HFD vs. WT-Con was attenuated in lncMGCKO HFD male and female mice (Figure S10). Compared to WT Con, the expression of lncMGC was significantly increased in BAT of WT HFD male (Figure 4E) and female mice (Figure 4J). In male mice, compared to WT Con, we did not observe a significant decrease in gene expression of *Ucp-1* and *Fis-1* despite decreases in their protein levels in WT HFD mice (Figure S11A); this may be due to post-transcriptional regulation since certain miRNA targets are regulated at both the post-transcriptional and translational levels.[51] However, gene expression of mitochondrial transcription factor A [(*Tfam*), a potential target of miR-3072 and miR-539),[52] *Ybx1*, and mediator of mitophagy PTEN-induced putative kinase 1 (*Pink-1*)[53] were significantly decreased in WT HFD but not in the corresponding lncMGC KO HFD mice compared to the respective controls (Figure S11A). Furthermore, partial reduction of mitophagy regulator Parkin RBR E3 ubiquitin-protein ligase (*Prkn*)[50] seen in WT HFD versus WT con was attenuated in lncMGC KO HFD mice (Figure S11A). In female mice, decreased gene expressions of *Ucp-1, Fis-1*, *Tfam*, *Ybx1*, and *Pink1* in WT HFD compared to WT Con were restored in lncMGC KO HFD mice (Figure S11B), while no significant differences in *Prkn* gene expression were detected between the groups. In addition, we measured the expression of miRs-379, -377, and -410 in the BAT from WT and lncMGC KO HFD male mice (Figure S11C). We observed that the expression of miR-379 was significantly decreased in lncMGC KO control and HFD mice compared to their respective controls (Figure S11C).

These data suggest that lncMGC/miR-379 axis can regulate key parameters involved in BAT mitochondrial function.

GapmeR targeting mouse lncMGC reduces weight gain and improves insulin sensitivity in HFD-fed mice

Locked nucleic acid (LNA)-modified GapmeRs (DNA-RNA hybrid antisense oligonucleotides) are optimized for specific RNA inhibition *in vitro* and *in vivo* and are particularly effective for inhibiting nuclear lncRNAs.[20,54] We recently demonstrated that GapmeR targeting miR-379 (the first miRNA in the lncMGC/miR379 cluster) efficiently reduced the expression of miR-379 and increased target genes in 3T3L1 preadipocyte cells.[31] Moreover, GapmeR targeting lncMGC significantly reduced lncMGC/miR379 in mouse models of diabetic kidney disease and in human and mouse renal cells and islets.[20,29] Here, we tested the potential effects of the GapmeR targeting mouse lncMGC (mlncMGC) on HFD-induced obesity and adipose dysfunction in WT-C57BL/6J mice (Figure S12A). We fed WT male and female mice with chow diet or HFD, and as expected, we observed significant weight gain in HFD-fed mice after two weeks (Figure S12B). Then, mice were randomly divided into 3 groups and injected with either negative control (NC) GapmeR (HFD/NC) or GapmeR targeting mlncMGC (HFD/Gap) at 5 mg/kg body weight once a week for an additional 8 weeks, while untreated HFD-fed mice were used as control (HFD) (Figure S12A).

Delivery and accumulation of mlncMGC GapmeR (red signals) into the gWAT was confirmed by an *in situ* hybridization assay with a fluorescent antisense LNA-modified probe (Figure 5A). mlncMGC expression was decreased in gWAT in HFD/Gap mice compared to the HFD/NC groups (Figure 5B).

We found that mice receiving the GapmeR targeting lncMGC showed significantly lower rates of weight gain in HFD/Gap mice vs. HFD/NC mice (Figures 5C and 5D), accompanied by reduced total body fat as assessed by Echo/MRI when compared to HFD or HFD/NC mice (Figure 5E). Total lean mass did not differ between these groups (Figure S13A).

At the end of the study, both male and female mice in the HFD groups (control and NC) exhibited higher BGLs compared to chow-fed control mice, with HFD/Gap mice showing a slight BGLs reduction (Figure S13B). Interestingly, HFD/Gap male mice exhibited significant improvement in hyperinsulinemia, HOMA-IR, glucose tolerance (GTT), and HOMA-B%, while HFD/Gap female mice showed a moderate improvement in these parameters

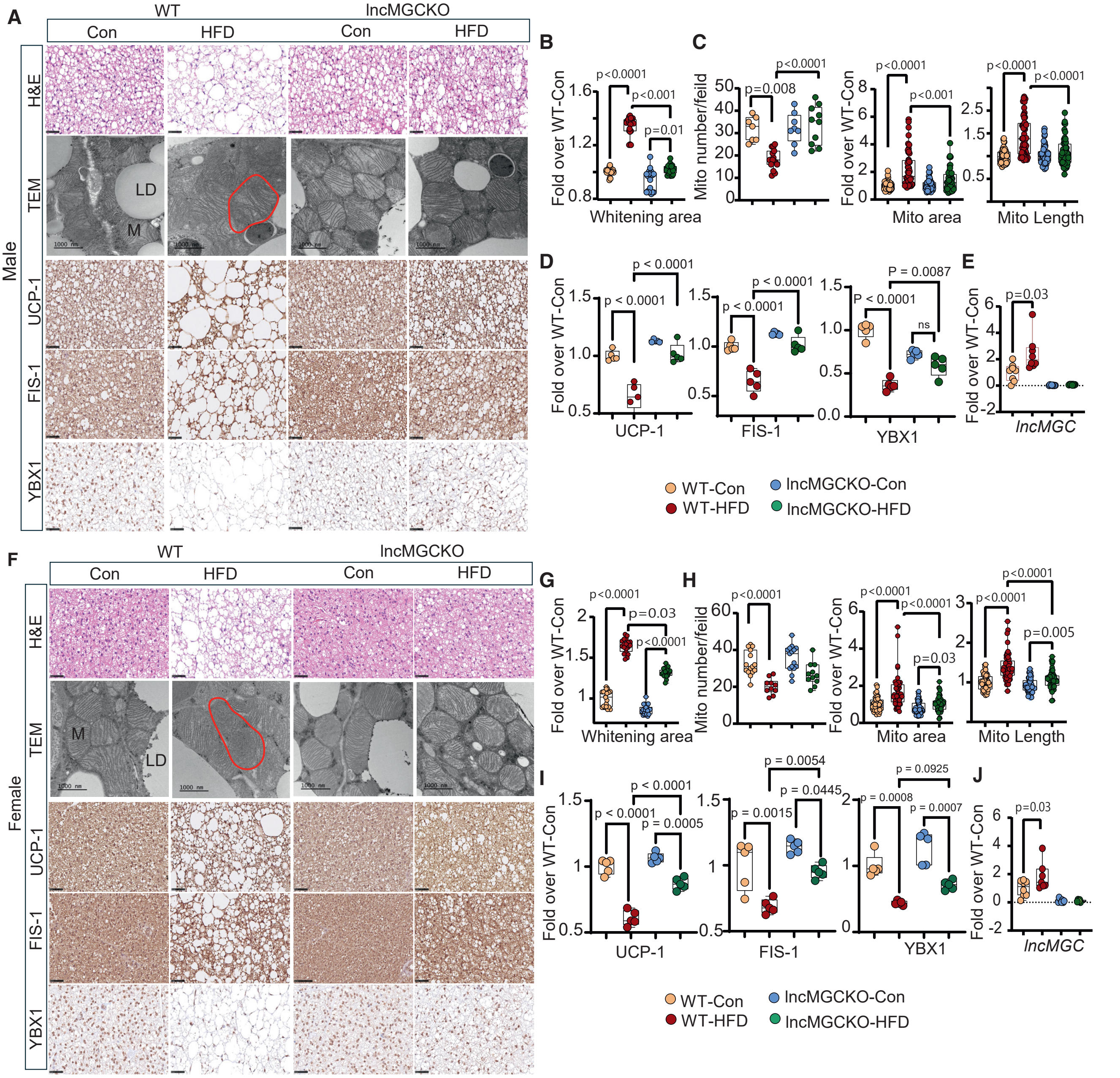

Figure 4. lncMGC KO improves mitochondrial structure and function markers in brown adipose tissue of HFD-fed mic

In male mice, (A) H&E staining shows increased lipid droplets and whitening of BAT in WT HFD, which are reduced in lncMGC KO HFD mice. TEM images show mitochondrial structure (Red line). IHC shows staining of UCP-1 (thermogenesis marker), FIS1 (mitophagy marker and target of miR-379), and YBX1 (RNA binding protein which regulates thermogenesis and mitophagy) in BAT. Quantitative analysis of (B) lipid droplet accumulation (H&E staining, whitening area) ($n = 20$ area/group). (C) mitochondrial number (n = 7–15 field), area ($n = 50$ mitochondria/field), and length ($n = 50$ mitochondria/field); (D) UCP-1 FIS1, and YBX1. $n = 5$/group. (E) Gene expression of lncMGC in BAT (n = 6–8/ group). In female mice, (F) H&E staining shows whitening of BAT in WT- HFD female mice, which was reduced in lncMGC KO HFD. TEM images showing mitochondrial structure (Red line), and IHC staining of UCP-1, FIS1, and YBX1 in BAT. Quantitative analysis of (G) lipid droplet accumulation ($n = 20$ area/group). (H) mitochondrial number ($n = 10$–15 field), area ($n = 50$ mitochondria/field), and length ($n = 50$ mitochondria/field), (I) UCP-1, FIS1, and YBX1. $n = 5$/group. (J) Gene expression of lncMGC in BAT (n = 7–8 mice/group). 20 weeks HFD. Scale bars, 50 μm. Statistical analyses were performed by two-way ANOVA with post-hoc Tukey test for multiple comparisons. The bar and whisker plot displays the distribution of the data. The whiskers extend from the minimum to the maximum values. Individual data points are overlaid as dots. Statistically significant p values are indicated in the bar graphs.

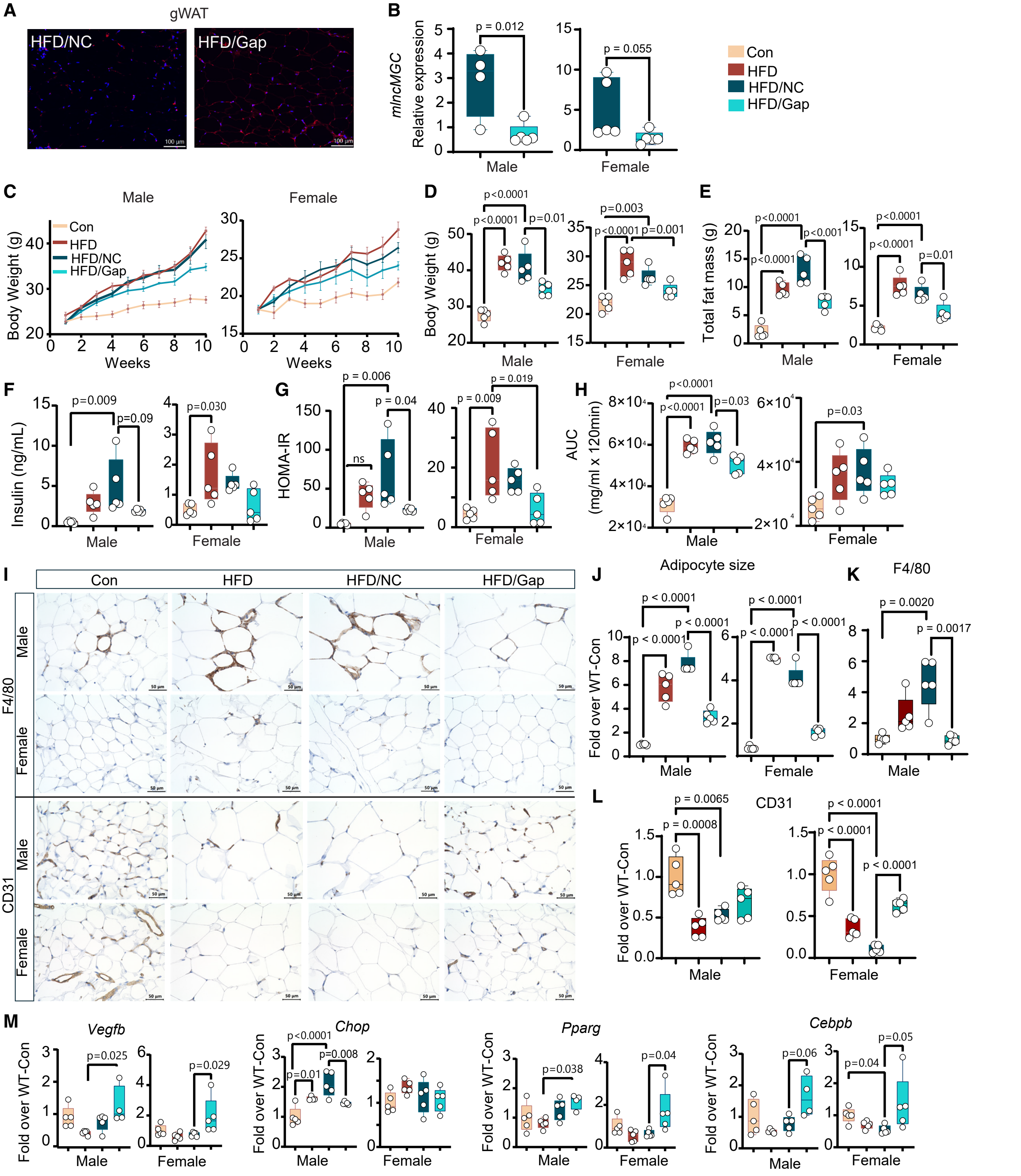

Figure 5. lncMGC-GapmeR treatment reduces the rate of weight gain and improves insulin sensitivity in HFD-fed mice

(A) Representative images show GapmeR accumulation in gWAT in HFD/NC and HFD/Gap mice by *in situ* hybridization with a fluorescent antisense LNA-modified probe. (B) Gene expression of mlncMGC (n = 4–5/group) in HFD/NC and HFD/Gap mice. (C–H)Weekly body weight gain, (D) endpoint body weight, (E) total body fat, (F) plasma insulin

(legend continued on next page)

when compared to respective HFD/NC mice (Figures 5F–5H and S13C). Moreover, pancreatic islet hyperplasia seen in HFD was alleviated in HFD/Gap mice (Figure S13D).

Histological examination and quantitative analysis showed that mlncMGC-GapmeR attenuated adipocyte hypertrophy in HFD/Gap mice versus HFD/NC (Figures 5I and 5J). Immunohistochemistry data showed that mlncMGC-GapmeR reduced gWAT inflammation (F4/80) in HFD male mice (Figures 5I and 5K). We did not observe distinct F4/80 positive cells in gWAT from female HFD mice (Figure 5I). The decreased level of CD31 (vascular endothelial marker) observed in HFD mice was also restored in gWAT of HFD/Gap mice (Figures 5I–5L), suggesting improved angiogenesis. This was further supported by data showing that in gWAT, *Vegfb (*an angiogenesis marker*)* gene expression was significantly increased in HFD/Gap compared to HFD mice (Figure 5M). The expression of *Chop* was significantly increased by HFD in male mice but was attenuated by lncMGC Gap treatment, suggesting ameliorated ER stress. *Pparg* and *Cebpb* (adipogenic markers) were increased in HFD/Gap compared to the HFD/NC group (Figure 5M). We have also measured the expression of some other cluster miRNAs, including miR-379, in the gWAT in control and HFD lncMGC-GapmeR-treated male and female groups. The expressions of miRs-379, -377, -380, -410, -411, and -495 were increased by HFD in gWAT in male mice. In female mice, miRs-379, 494, -410, -411, and -495 were increased by HFD in gWAT (Figure S14). Although we observed lncMGC GapmeR-mediated reduction in the expression of these miRNAs in HFD/Gap mice, the changes were not statistically significant compared to HFD/NC mice (Figure S14). Unlike targeting a single microRNA such as miR-379, targeting lncMGC provides broader regulatory effects. Our findings demonstrate that inhibition of lncMGC with a GapmeR confers significant metabolic benefits and anti-obesity effects.

GapmeR targeting mlncMGC improves BAT mitochondrial structure and function markers in HFD-fed mice

Using *in situ* hybridization, we confirmed that the GapmeR accumulated in BAT (Figure 6A). In parallel, gene expression of mlncMGC was significantly reduced in BAT of HFD/Gap mice compared to HFD/NC in male and female mice (Figure 6B). BAT whitening observed in HFD mice was attenuated in HFD/Gap male (Figure 6C) and female mice (Figure 6D). IHC staining and quantitative analysis showed the levels of UCP-1, FIS-1, and mitochondrial oxidative phosphorylation marker succinate dehydrogenase B (SDHB)/mitochondrial complex II,[55] were significantly increased in BAT in HFD/Gap male (Figures 6C and 6E) and female (Figures 6D and 6F) mice versus HFD/NC mice. Decreased level of YBX1 in HFD and HFD/NC mice (Figures 6C and 6F) was restored only in HFD/Gap female mice (Figures 6D and 6F). We found that mlncMGC GapmeR significantly increased the gene expression of *Ucp-1*, *Pparg*, and *Tfam* in HFD/Gap male mice compared to HFD/NC mice (Figure S15A). However, we did not observe any differences in the gene expression of other putative targets [*Fis-1,* thermogenesis regulator peroxisome proliferator-activated receptor γ coactivator 1α (*Ppargc1α*), *Ybx1*, *Pink*, *Prkn*] between the HFD groups (Figure S15A). On the other hand, the altered expression of these genes was fully or partially attenuated in corresponding HFD/Gap female mice, revealing some sex-specific differences (Figure S15B). The observation that certain endpoints show significance only in one sex suggests potential sex-specific biological differences in other endpoints tested that may influence the measured outcomes. Female C57BL/6 mice have been reported to be less prone to HFD diet-induced obesity relative to male mice.[56] These differences could be attributed to various factors, including hormonal regulation, genetic patterns, metabolic differences, and variations in immune responses between males and females. For instance, sex hormones such as estrogen can modulate signaling pathways differently, potentially affecting gene expression, insulin sensitivity, inflammatory responses, or sex differences in gut microbiome.[57–59] Additionally, epigenetic modifications unique to each sex may contribute to variations in chromatin accessibility and gene regulation, influencing the observed effects.[60]

GapmeR targeting human lncMGC protects partially humanized lncMGC mice from obesity and adipocyte hypertrophy

In WAT samples obtained from obese/overweight human donors, we observed that human lncMGC expression was significantly increased as compared to lean individuals (Figure 7A). To determine the translational potential of targeting lncMGC to reduce obesity/metabolic dysfunction in humans, we used novel partially humanized lncMGC (hlncMGC) mice generated in our laboratory using CRISPR-Cas9 editing in which key non-homologous mouse sequences are replaced with human sequences.[29] hlncMGC homo (500 bp) mice were selected for experiments as recently described.[29]

Based on our findings that mlncMGC-GapmeR protected further weight gain in WT HFD mice, we tested whether hlncMGC-GapmeR reduces/slows down weight gain in hlncMGC HFD mice. We fed hlncMGC female mice with a chow diet or HFD for two weeks and observed significant weight gain in HFD-fed mice after two weeks (Figure S16B). They were then randomly treated with

levels, (G) Homeostatic model assessment HOMA-IR, and (H) area under the curve (AUC) for glucose tolerance test (GTT) in male and female mice. (I–L) IHC staining of F4/80 (macrophage marker) and CD31 (endothelial cell marker) in gWAT in male and female mice. Scale bars, 50 μm; 40× magnification. Quantitative analysis of (J) adipocyte size, (K) F4/80, and (L) CD31. $n = 5$/group. (M) Gene expression of *Vegfb* (angiogenesis marker) *Chop* (ER stress marker) *Pparg* and *Cebpb* (adipogenesis markers) in gWAT in male and female mice. Control chow-diet (Con), high-fat diet (HFD), negative control (NC) GapmeR (HFD/NC), GapmeR targeting lncMGC (HFD/Gap). Statistical analyses were performed by two-way ANOVA with post-hoc Tukey test for multiple comparisons. XY graphs show the mean (SD). The bar and whisker plot displays the distribution of the data. The whiskers extend from the minimum to the maximum values. Individual data points are overlaid as dots. Statistically significant *p*-values are indicated in the bar graphs.

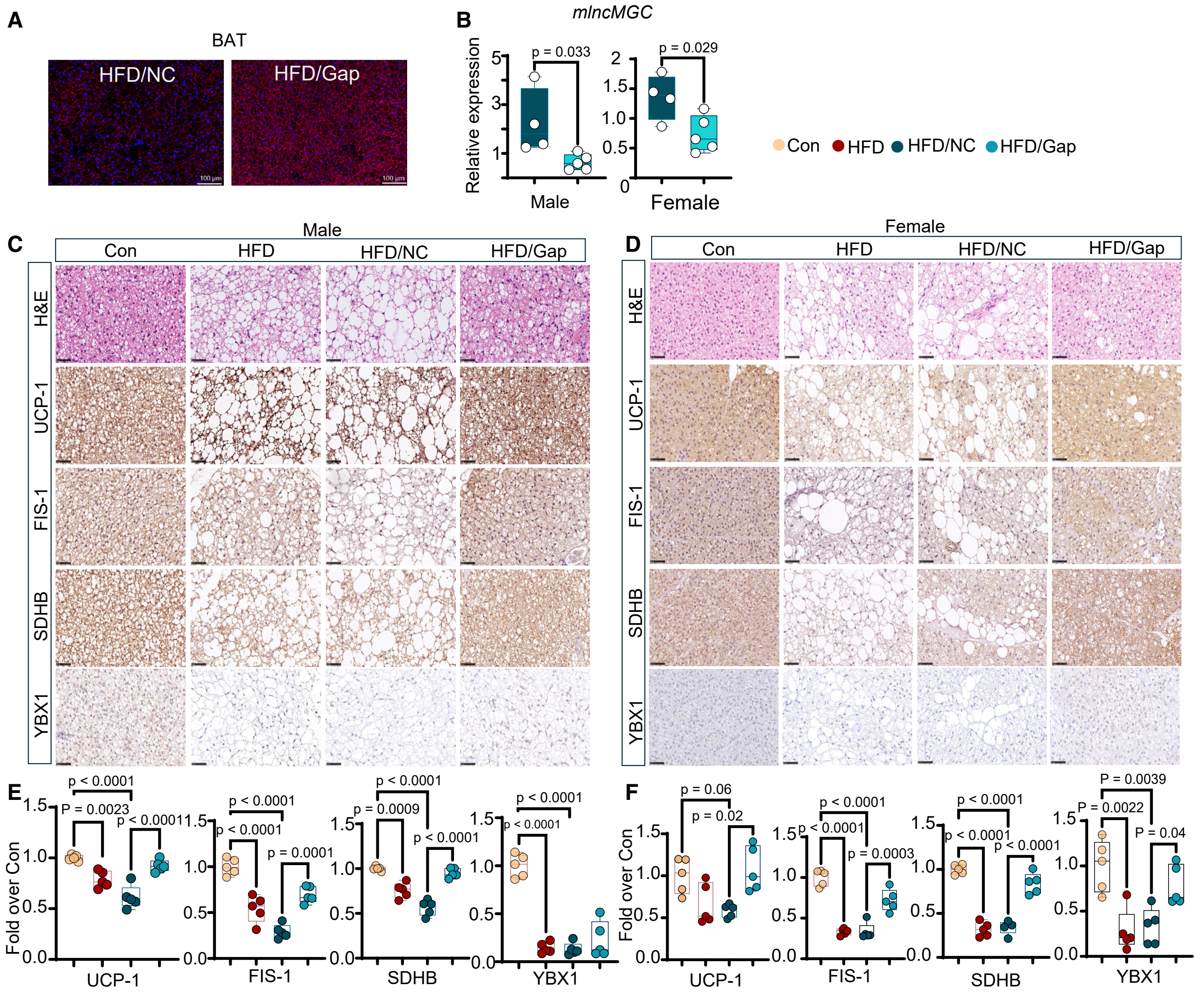

Figure 6. GapmeR targeting mouse lncMGC improves mitochondrial structure and function markers in brown adipose tissue of HFD-fed mice
(A) Representative images show GapmeR accumulation in BAT in HFD /GapmeR mice by *in situ* hybridization with a fluorescent antisense LNA-modified probe. Scale bars, 100 μm; 20× magnification. (B) Gene expression of lncMGC in BAT in HFD/NC and HFD/Gap male and female mice. (n = 4–5 mice/group). In male mice, (C) H&E staining shows reduced lipid droplets and whitening of BAT in HFD mice treated with lncMGC-GapmeR. (C) IHC staining and (E) quantitative analysis of UCP-1, FIS1, SDHB (mitochondrial respiratory marker), and YBX1. *n* = 5/group. In female mice, (D) H&E staining reveals an increase in lipid droplets and whitening of BAT in HFD mice, which is reduced in HFD mice treated with lncMGC-GapmeR. (D) IHC staining and (F) quantitative analysis of UCP-1, FIS1, SDHB, and YBX1.*n* = 5/group.10 weeks HFD. Scale bars, 50 μm; 40× magnification. Control chow-diet (Con), high-fat diet (HFD), negative control (NC) GapmeR (HFD/NC), GapmeR targeting lncMGC (HFD/Gap). Statistical analyses were performed by two-way ANOVA with post-hoc Tukey test for multiple comparisons. The bar and whisker plot displays the distribution of the data. The whiskers extend from the minimum to the maximum values. Individual data points are overlaid as dots. Statistically significant *p* values are indicated in the bar graphs.

NC-GapmeR (hHFD/NC) or GapmeR targeting hlncMGC (hHFD/Gap) 5 mg/kg/once a week for 8 weeks. Untreated HFD (hHFD) mice served as control (Figure S16A). As shown in Figures 7B and 7C, hHFD/Gap mice gained weight at a significantly slower rate compared to hHFD/NC mice. The increase in total fat mass observed in hHFD and hHFD/NC mice was significantly reduced in hHFD/Gap mice (Figure 7D).

We confirmed GapmeR accumulation in gWAT using *in situ* hybridization (Figure 7E, red signals) and a significant decline in hlncMGC expression in both gWAT and BAT of hHFD/Gap mice compared to hHFD/NC mice (Figure 7F). Histology of gWAT showed significant improvements in adipocyte hypertrophy in hHFD/Gap mice compared to HFD/NC mice (Figure 7H). In addition, the decrease in CD31 observed in HFD/NC mice was significantly reversed

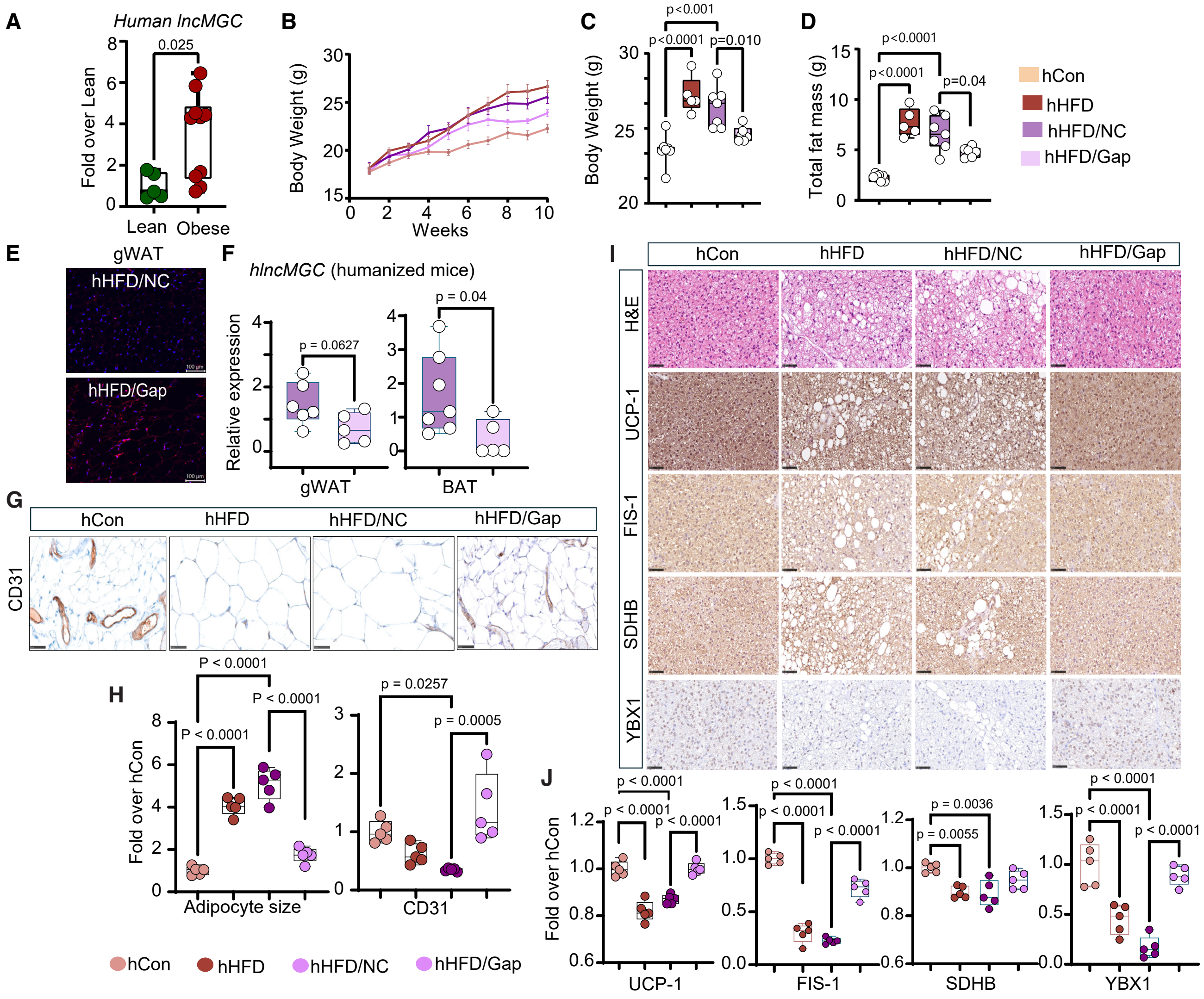

Figure 7. hlncMGC-GapmeR treatment reduces the rate of weight gain and improves key functional markers in gWAT and BAT of partially humanized lncMGC HFD-fed female mice

(A) Expression of lncMGC in white adipose tissue (WAT) samples obtained from human lean and overweight/obese donors. (B) Weekly and (C) endpoint body weight, (D) total body fat in partially humanized female mice fed HFD for 10 weeks. (E) Representative images show GapmeR accumulation (red signals) in gWAT in partially humanized lncMGC HFD/GapmeR (hHFD/Gap) female mice by *in situ* hybridization. (F) Expression of hlncMGC in gWAT in negative control (NC) GapmeR (hHFD/NC) and hHFD/Gap humanized mice (n = 5–7/group) and (G) IHC staining of CD31 in gWAT sections. (H) Quantitative analysis of adipocyte size and CD31. *n* = 5/group. (I) H&E staining shows a significant reduction of lipid droplets and whitening of BAT in hHFD/Gap mice. (I) IHC staining and (J) quantitative analysis of UCP-1, FIS1, SDHB, and YBX1 in BAT. *n* = 5/group. Scale bars, 50 μm; 40× magnification. Control chow-diet (hCon), high-fat diet (hHFD). Statistical analyses were performed by two-way ANOVA with post-hoc Tukey test for multiple comparisons. XY graph shows mean SD. The whiskers extend from the minimum to the maximum values. Individual data points are overlaid as dots. Statistically significant *p* values are indicated in the bar graphs.

in hHFD/Gap mice, indicating improvement in angiogenesis (Figures 7G and 7H). Consistent with our observations in mlncMGC KO and WT GapmeR HFD mice, BAT whitening was markedly attenuated in hHFD/Gap female mice (Figure 7I). IHC examination and quantification analysis revealed that UCP-1, FIS-1, SDHB, and YBX1 levels were significantly elevated in HFD/Gap versus HFD/NC mice (Figures 7I and 7J).

LncMGC controls key regulators of adipogenesis and mitochondrial gene programs

To further interrogate regulatory connections, we examined if the factors related to adipogenesis and mitochondrial function that were altered in lncMGC deficient mice are also directly altered by the lncMGC GapmeR *in vitro* in adipose cells. We treated cultured 3T3L1 preadipocytes and HIB 1B brown adipocytes with

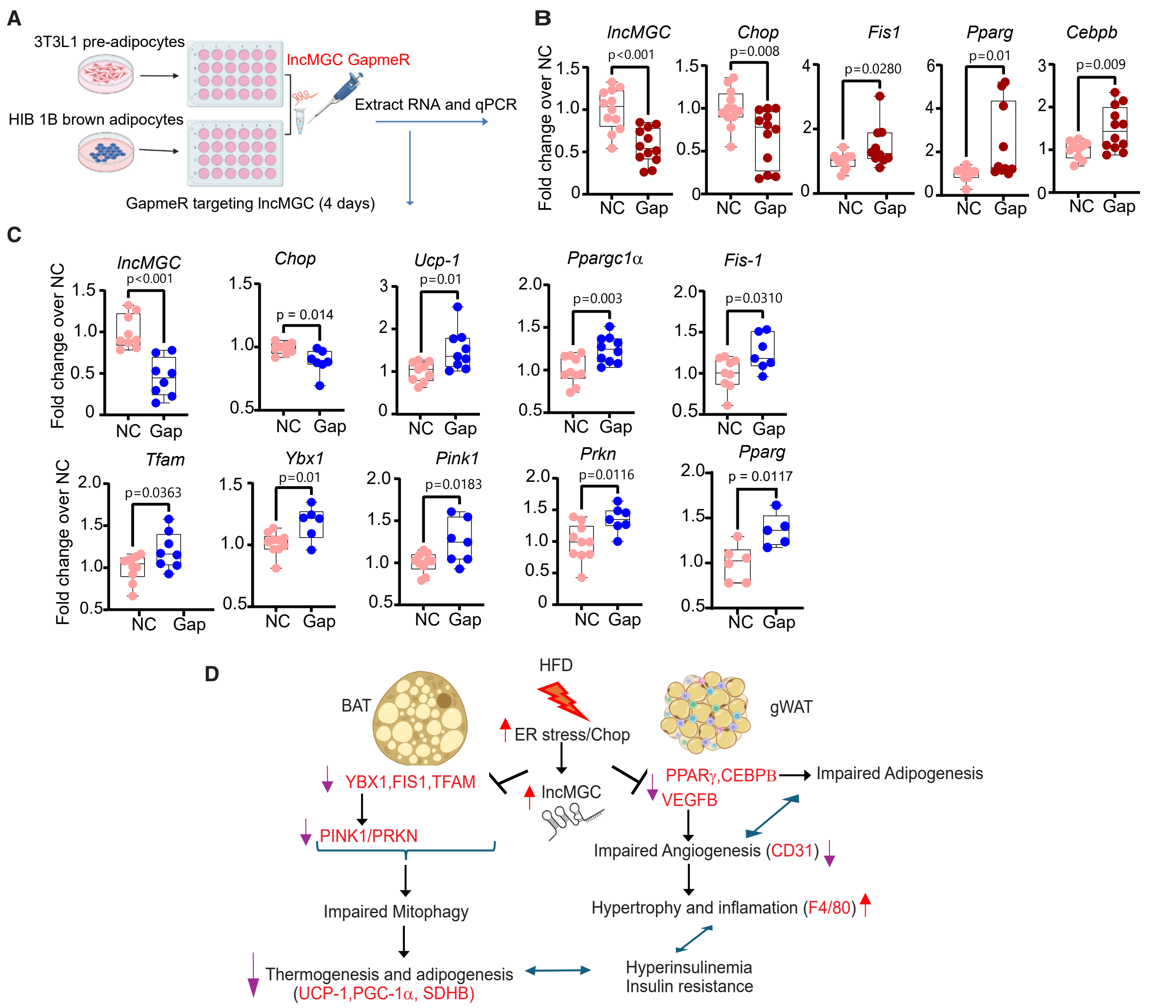

Figure 8. lncMGC regulates key factors related to adipogenesis and mitochondrial gene program

(A and B) 3T3L1 pre-adipocytes were transfected with negative control GapmeR (NC) or mlncMGC-GapmeR (1 μmol/L) for 4 days by Gymnosis. (B) gene expression of lncMGC, *Chop*, *Fis1*, *Pparg*, and *Cebpb* in 3T3L1 preadipocytes (n = 10–12/group). (A, C) HIB 1B brown adipocytes were transfected with NC or lncMGC-GapmeR (1 μmol/L) for 4 days by Gymnosis. (C) gene expression of lncMGC, and mitochondrial markers in HIB 1B brown adipocytes (n = 5–10/group). Data were analyzed using Student's t-tests for comparisons between two groups. The whiskers extend from the minimum to the maximum values. Individual data points are overlaid as dots. Statistically significant p-values are indicated in the bar graphs. (D) HFD increases ER stress and lncMGC in gWAT and BAT. In gWAT and BAT, lncMGC regulates adipose tissue function, in part, by influencing adipogenesis, angiogenesis, inflammation, and mitochondrial markers associated with thermogenesis and mitophagy, potentially through mechanisms involving ER stress. These processes contribute to metabolic dysfunction and insulin resistance in obesity. Abdollahi and colleagues show that decreasing levels of a long noncoding-RNA, lncMGC, by genetic knockout or GapmeR antisense oligonucleotides can attenuate obesity, obesity induced adipose tissue dysfunction, and insulin resistance in mice. Targeting lncMGC may represent a promising therapeutic strategy for treating adipose dysfunction, obesity, and associated complications.

NC or lncMGC GapmeR [(1 μmol/L) for 4 days] (Figure 8A). Expression of lncMGC was effectively suppressed in 3T3L1 and HIB 1B cells treated with lncMGC GapmeR (Figures 8B and 8C). Compared to the NC group, lncMGC GapmeR-treated 3T3-L1 cells depicted significantly decreased *Chop* expression, while *Fis1*, *Pparg*, and *Cebpb* expression levels were upregulated (Figure 8B). The expressions of genes related to ER stress and mitochondrial function, and adipogenesis were significantly improved by GapmeR treatment in HIB 1B brown adipocytes compared to NC controls (Figure 8C). As miR-379 is one of lncMGC's major downstream effectors targeting *Vegfb*, *Fis1*, and potentially Ybx1, (RNA-binding protein and a potential target of miR-379), we performed

miR-379–lncMGC axis rescue in HIB 1B brown adipocytes (Figure S17). The relative expression of miR-379 was significantly increased in cells transfected with miR-379 mimics (901.9 ± 321.8 vs. 1.185 ± 0.68 in NC) and reduced following treatment with lncMGC targeting GapmeR (175.7 ± 81.26 vs. 901.9 ± 321.8 in miR-379 mimics). Furthermore, transfection with miR-379 mimics enhanced the expression of lncMGC and *Chop*, an ER stress marker and positive regulator of lncMGC expression, which was significantly reduced by lncMGC-GapmeR treatment (Figure S17). miR-379 enhances ER stress by repressing ER-associated degradation components such as EDEM3, which also elevates CHOP expression. Since CHOP transcription factor directly activates the lncMGC/miR-379 cluster locus,[20] this creates an autoregulatory feedback amplification loop where increased miR-379 can in turn upregulate lncMGC expression. Furthermore, expressions of *Vegfb*, *Fis-1*, and *Ybx1* were significantly increased by lncMGC-GapmeR treatment (Figure S17), indicating the observed phenotype is caused (at least partly) by decreased levels/activity of lncMGC.

Together, our data suggests that HFD-induced ER stress upregulates lncMGC via *Chop* (ER stress marker and regulator of lncMGC expression), which in turn, downregulates key targets, including *Vegfb* (angiogenesis marker and target of miR-379) and *Pparg* and *Cebpb* (adipogenic markers), leading to impaired angiogenesis and adipogenesis in gWAT. These changes exacerbate gWAT inflammation and insulin resistance, which can be attributed at least in part to the induction of lncMGC (Figure 8D). Our findings also suggest that lncMGC dysregulates BAT function, which could be mediated, at least in part, by influencing mitochondrial markers associated with thermogenesis (UCP-1, PGC-1α) and mitophagy (FIS-1, PINK1), potentially through mechanisms involving ER stress (Figure 8D). As a result, targeting and reducing lncMGC by genetic deletion or with a GapmeR can potentially provide protection against HFD-induced obesity and adipose dysfunction via the indicated mechanisms (Figure 8D).

DISCUSSION

In this study, we used a combination of genetic KO mice as well as GapmeR antisense oligo targeting to investigate the role of the lncRNA lncMGC in HFD-induced obesity and adipose tissue dysfunction and explore underlying factors/mechanisms. We demonstrate that HFD upregulates lncMGC in white and brown adipose tissues in mice, and lncMGC levels are also increased in adipose samples obtained from obese humans, suggesting lncMGC upregulation can drive WAT and BAT dysfunction in obesity. Our data suggest that lncMGC exerts these effects via modulating key targets involved in ER stress, adipogenesis, thermogenesis, and mitochondrial function in both WAT and BAT. Notably, inhibition of lncMGC with a GapmeR ameliorated HFD-induced obesity, IR, and mitochondrial function parameters in both WT and partially humanized lncMGC mice, supporting the therapeutic potential of targeting lncMGC for obesity and adipose tissue dysfunction and further underscoring the emerging utility of noncoding RNA therapeutics (Figure 8D).

Given the critical role of BAT in thermogenesis and maintaining energy balance, promoting BAT activity has emerged as an attractive preventive and therapeutic strategy for combating obesity.[61] BAT possesses abundant mitochondria required for adaptive thermogenesis. Damage to mitochondria in BAT is associated with increased intracellular lipid droplets, reflecting a reduced capacity for fatty acid oxidation, leading to oxidative stress and further damage to mitochondrial structures. One of the major observations that we made in this study is that lncMGC KO, or HFD-fed mice treated with lncMGC GapmeR depict better BAT mitochondrial structures and functions relative to WT HFD, which could be potentially mediated through modulation of key mitochondrial gene programs involved in thermogenesis and mitophagy. LncMGC KO HFD mice also displayed preservation of energy balance. In parallel, mitochondrial markers were upregulated, including those in thermogenesis (UCP-1 and PGC-1), mitophagy-driven specific proteins/genes (FIS-1,[18] Pink1, and Prkn),[50] and SDHB, a mitochondrial complex II respiratory enzyme required for oxidative phosphorylation.[55,62] Furthermore, TFAM, crucial for mitochondrial DNA stability and BAT development and differentiation,[52] was increased by lncMGC inhibition in HFD-fed mice. Studies show that adipocyte-specific knockout of TFAM results in adipose inflammation, BAT whitening, severe lipodystrophy, insulin resistance, and decreased EE in obese mice.[63] In line with our *in vivo* results, we observed increased levels of mitochondrial function markers in lncMGC GapmeR-treated cultured brown adipocytes, providing direct support for the regulatory effect of lncMGC on adipocyte mitochondrial functions/health.

We previously showed that lncMGC expression was increased in renal glomeruli of mouse models of diabetic kidney disease,[20] in TGF-beta or high glucose-treated kidney mesangial cells,[27,37] and isolated pancreatic islets from T2D donors[29] and is regulated by CHOP (ER stress-responsive transcription factor).[20] We also identified key proteins that interact with lncMGC RNA in kidney mesangial cells, including several RNA-binding proteins like IQGAP, SMARCA5, SMARCC2, DBC1, BAT2, and YBX1, suggesting lncMGC may also function via epigenetic mechanisms involving interactions with these proteins, thereby altering target gene expression, including lncMGC itself, as reported by us.[27] Interestingly, YBX1, a potential target of miR-379, was also increased by lncMGC inhibition in HFD-fed mice. YBX1 was reported to promote brown fat development and heat production through mitophagy mediated by PINK1 and PRKN.[50] These observations suggest that increased lncMGC in obesity conditions potentially modulates lncMGC/YBX1 interactions to (mis)regulate YBX1 function and/or expression, leading to altered brown adipocyte thermogenic function via PINK1/PRKN-mediated mitophagy.[50] However, further studies are needed to validate these connections.

It is well known that impaired insulin receptor (IRS)/AKT signaling leads to defective adipogenesis in obesity[64,65] and contributes to

increased inflammation, which, in turn, hinders glucose disposal, resulting in a compensatory rise in beta-cell insulin production and hyperinsulinemia.[66] Interestingly, our DSP data showed that the downregulation of insulin receptors (IRS1, IRS2) observed in WT HFD was alleviated in BAT from lncMGC KO HFD mice. As mentioned earlier, lncMGC can interact with the RNA-binding protein IQGAP, which acts as a scaffold for insulin receptors and plays a role in signaling pathways that regulate metabolic processes, including insulin signaling and glucose metabolism.[67] Such interaction may be a potential mechanism by which lncMGC contributes to impaired insulin signaling in obesity, with attendant dysregulated adipose lipid metabolism and thermogenesis.

Gene ontology and differential gene expression analysis revealed that lncMGC inhibition could also restore pathways related to the generation of precursor metabolites and energy. For example, Cox7c and b, subunits of cytochrome *c* oxidase (Complex IV) in the mitochondrial electron transport chain,[43] *Mdh1* (malate dehydrogenase 1), a cytosolic enzyme that plays a crucial role in cellular metabolism,[68] and *Etfβ* (electron transfer flavoprotein subunit beta), an essential protein involved in mitochondrial function, particularly in the process of fatty acid oxidation and energy metabolism,[69] and *Uqcrq* (ubiquinol-cytochrome c reductase, complex III subunit VII), essential for cellular energy production[70] depicted higher levels in the setting of lncMGC inhibition in HFD mice. We found that inhibiting lncMGC could also restore pathways associated with the fatty acid metabolic process and fatty acid oxidation: these processes are particularly important in BAT for generating heat through non-shivering thermogenesis.[61,71] For example, HFD-induced disruption of adiponectin receptor2 (*Adipor2*) was partially ameliorated by lncMGC KO, which can restore adiponectin signaling, enhance glucose uptake, and fatty acid breakdown[72] and thereby cf. protective effects against metabolic disorders. Collectively, these data indicate that lncMGC directly or indirectly regulates metabolism as well as mitochondrial functions via modulating pathways involved in thermogenesis.

Our findings showing increased whole-body EE and weight loss in lncMGC KO mice may be due, at least in part, to increased BAT mitochondrial activity since BAT is a primary energy source for thermogenesis. Additionally, we found that reducing lncMGC levels in HFD mice can improve angiogenesis and adipogenesis (two crucial processes in adipose tissue function) in the gWAT. It is important to note that since our GapmeR targeting lncMGC is not cell/tissue-specific, it can accumulate in various organs, including the pancreas,[29] liver, and kidney.[20] Consequently, the protective effects observed *in vivo* may be influenced not only by lncMGC inhibition in the gWAT and BAT but also by the impact on these other metabolic organs that regulate body weight and metabolism. However, our *in vitro* data in 3T3 adipocytes and brown adipocytes (Figure 8A) show that lncMGC can directly regulate key parameters of WAT function and BAT mitochondrial health.

Previous studies have shown that exercise independently reduces body weight and fat mass by increasing EE, enhancing mitochondrial function, and improving adipose tissue remodeling and angiogenesis.[73,74] Therefore, incorporating exercise would likely attenuate body weight and fat accumulation across all groups, regardless of lncMGC knockout and moreover, our metabolic data showed no significant difference in movement between the genotypes.

Our new data here are aligned with our earlier findings, which demonstrated that the upregulation of miR-379 (the first miRNA in the miR-379/lncMGC cluster) in adipose tissues from HFD-fed mice and obese human donors is associated with increased cellular stress, impaired metabolic profiles, and disrupted adipose tissue function.[31] It is possible that some of the protective effects seen with lncMGC deficiency in the current study are due to the reduction in miR-379, especially because we observed that some miR-379 targets were also upregulated during lncMGC KO or GapmeR treatment. However, other non-miR-379 targets were also altered, implying other mechanisms, which could be dependent at least in part on other miRNAs in the lncMGC cluster. However, besides miR-379, other cluster miRNAs such as miR-494 (targeting ATF3, inhibiting CHOP, or potentially targeting YBX1) are also hosted by lncMGC and may also act as effectors. Furthermore, as we discussed, apart from working through effector miRNAs embedded within lncMGC, lncMGC RNA can also regulate gene expression via RNA-binding proteins and chromatin opening/accessibility by interacting with nucleosome remodelers, such as SMARCA5 and thereby altering target gene expression, including lncMGC itself, as reported by us recently.[27] Together, these data and observations suggest miR-379 is a key, but not the only downstream effector of lncMGC. Therefore, targeting lncMGC is different from targeting the single microRNA miR-379. Targeting lncMGC may have more beneficial effects for obesity treatment than targeting only miR-379 or other miRNAs in the cluster. It is well known that GLP-1 receptor agonists (GLP-1-RAs) have multiple beneficial effects and reduce body weight by activating hypothalamic pro-opiomelanocortin (POMC) and cocaine- and amphetamine-regulated transcript (CART) neurons to suppress appetite.[75–77] In contrast, our study demonstrates that targeting lncMGC reduces body weight through complementary and distinct peripheral mechanisms, specifically by improving obesity-induced dysfunctional pathways within adipose (and likely other metabolic) tissues. In the future, a head-to-head comparison of GLP-RAs with lncMGC targeting can highlight the value of the latter as an alternative treatment for obesity.

Taken together, our findings reveal that obesity induced by HFD in mice can increase lncMGC expression in gWAT through ER stress signaling, leading to elevated CHOP expression. This, in turn, augments ER stress, and alters the expression of key target genes and proteins that lead to impaired angiogenesis and adipogenesis in gWAT and contribute to gWAT inflammation and insulin resistance. In parallel, obesity also upregulates lncMGC in BAT and downregulates several targets that are protective for mitochondrial function, including those involved in thermogenesis and mitophagy. Together, these regulatory pathways establish a vicious positive feedback loop involving increased ER stress, inflammation, disrupted

adipogenesis, and mitochondrial dysfunction (Figure 8D). Collectively, our study highlights the detrimental effects of lncMGC upregulation in adipose tissue in obesity and suggests that targeting lncMGC could hold therapeutic potential for treating adipose dysfunction, obesity, and its associated complications.

MATERIALS AND METHODS

Animal studies

All animal studies were conducted according to protocols approved by the Institutional Animal Care and Use Committee at the Beckman Research Institute of the City of Hope National Medical Center.

LncMGC knockout and partially humanized lncMGC mice obtained by CRISPR-Cas9 genome editing

LncMGC KO male and female mice were generated using CRISPR-Cas9 editing as described by us.[29,78] The partially humanized lncMGC (hlncMGC) mice were also generated using CRISPR-Cas9 editing in which the non-homologous mouse sequences were replaced with human sequences as described.[29]

Mouse models of obesity using high-fat diet

Eight-week-old male and female C57BL/6J WT, lncMGC KO, and partially humanized lncMGC female mice were randomly divided into groups and fed a control chow or HFD [(60% kcal from fat) (D12492I, Research Diets, New Brunswick, USA)][30,31] for 10 or 20 weeks. Body weights and BGLs were measured during the experiments. At the end of the studies, mice were fasted overnight, and then, after euthanasia, plasma was collected to measure insulin levels and lipid profile including triglyceride (TG) and TC. Perigonadal gWAT, interscapular brown adipose tissue (BAT), and pancreata were harvested for molecular and histological analysis (Figures S1, S12, and S16).

Human adipose tissue samples

We obtained discarded, de-identified human visceral adipose tissue from the Southern California Islet Cell Resource Center (City of Hope). The study was approved as exempt by the City of Hope Institutional Review Board. The visceral WAT samples were classified into two groups—lean and overweight/obese based on the donor's body mass index (BMI) (Table S1).

GapmeRs

The LNA modified GapmeRs targeting mouse or human lncMGC used in this study have been reported.[29] They are GapmeRs targeting mouse lncMGC [(mlncMGC), MGC10], ATTtggcagtgggAAG, GapmeR targeting human lncMGC, [(hlncMGC),HMGC10], GATttggcattggAAG; GapmeR NC, 5′-ATTttattcggaGCT-3′. All uppercase: LNA; lowercase: DNA, full phosphorothioate. They were obtained from Integrated DNA Technologies (Integrated DNA Technologies, IDT, USA).

Metabolic assays

For assessment of GTT, mice fasted for 5 h, and after measuring fasting BGLs, mice were injected with glucose (intraperitoneally, 2 g/kg body weight), followed by measuring BGLs consecutively at 15, 30, 90, and 120 min post glucose injection.[31] BGLs were monitored with a glucometer (Alpha Track), and the area under the curve (AUC) was calculated using GraphPad Prism software. Plasma insulin levels were measured using a mouse insulin ELISA kit (Crystal Chem, 90080). Plasma lipid profile was enzymatically measured using the following kits: triglycerides reagent, TR22421, Thermo Scientific; TC Assay Kits, STA-384, Cell Biolabs, INC, according to the provided protocols. Homeostatic model assessment of insulin resistance (HOMA-IR) and HOMA-B% (estimates steady state β-cell function)[79] were calculated according to the following formula: HOMA-IR: fasting insulin (μU/L) × fasting glucose (nmol/L)/22.5[31,80] and HOMA-B%: (20 x fasting insulin)/(fasting glucose-3.5) %.[79] Metabolic parameters, such as EE, food intake, water consumption, and locomotor activity, were measured using an indirect calorimetry cage system (PhenoMaster, TSE Systems, Bad Homburg, Germany). Body composition, including total fat and lean mass, were measured using quantitative magnetic resonance imaging (EchoMRI; Echo Medical Systems, Houston, TX) by City of Hope Comprehensive Metabolic Phenotyping Core.[31] The total duration of HFD was 20 weeks, but metabolic parameters and glucose GTT were measured during weeks 16–18 of the HFD period. Peripheral body temperature was measured using an infrared temperature gun (thermal imaging camera RoHs).

Sample processing for NanoString GeoMx digital spatial profiler and quality control analysis

Tissue microarray (TMA) sections of BAT harvested from WT and lncMGC KO female mice were prepared, and slides were processed following the protocol of RNA slide preparation for formalin-fixed paraffin-embedded (FFPE) samples as described in the GeoMx DSP manual slide preparation user manual (NanoString technologies, GeoMx-DSP-manual-slide-preparation, MAN-10150). Briefly, deparaffinization and rehydration, antigen retrieval (invitrogen, IHC antigen retrieval solution, Cat#00-4956-58), and proteinase K digestion (1 μg/ml, at 37°C) were performed. Mouse WTA probes (GeoMx WTA, mouse RNA probe for NGS, catalog #121401103) were hybridized in Buffer R (GeoMx RNA Slide Prep Kit PCLN catalog #121300313) and incubated in a hybridization humidity chamber (UVP, HB-1000 Hybridizer) overnight at 37°C. To remove nonspecifically bound probes, slides were washed in 50% formamide/2× SSC (Thermo Fisher, catalog AM9763, USA) at 37°C (2 washes/25 min each), then slides were blocked and incubated with antibodies diluted with 200 μL Buffer W (GeoMx RNA Slide Prep Kit PCLN catalog 121300313). Then, immunostaining was performed for the detection of the region of interest (ROI) with UCP-1, CD45, and SYTO13 markers. Briefly, morphology marker solution was prepared in the following proportions per slide: 22 μL SYTO (GeoMx nuclear stain morphology kit, catalog 121300303), 5.5 μL UCP-1 (Protentech, catalog 23673-1-AP), 5.5 μL CD45 (GeoMx solid tumor TME morphology, mouse FFPE RNA compatible, catalog 121300315), and 181.5 μL Buffer W (GeoMx RNA slide prep kit PCLN catalog 121300313) for a total volume of 220 μL/slide. Slides

were incubated in this solution at room temperature for 1 h followed by 2 consecutive SSC washes, then, slides were loaded for ROI selection following GeoMxTM digital spatial profiler instrument user manual (Figure S5).

After ROI selection based on UCP-1 positive cells, WTA probe tags were selectively isolated (UV-cleaved) and collected from each of these ROIs, then transferred to individual wells of a collection plate on the DSP. The collections were processed for library preparation following the manual of MANUAL GeoMx DSP NGS Readout (MAN-10153-06). The quality of the library was assessed by the Bioanalyzer DNA 1000 assay (Agilent, catalog 5067-1504). The library was then sequenced on Illumina NextSeq2000. The fastq files were converted to DCC files using the software of GeoMx-NGS Pipeline; the DCC files were loaded on GeoMx DSP for QC, normalization. Q3 data was used to generate the heatmap.log2 fold change ±0.378 and p value <0.05.

ROIs were selected for transcript quantification, with four ROIs from the wild type control (WT Con), six from WT HFD, five from lncMGC KO Con, and seven from the group lncMGC KO HFD. The minimum nuclei we set up is 100, and the actual minimum nuclei count for the experiment is 158. The QC data were filtered by segment: keep segments with >10% targets above threshold, all 22 ROI passed the QC and filtration. This data are further filtered by target: keep targets with greater 10% segments above threshold. 8320 out of 19963 targets passed the filter. After filtration, the Q3 data were generated and used for all the analyses. A summary of individual ROI selections is provided in Table 3. Principal-component analysis (PCA) of RNA-seq data from brown adipose tissue was performed on the transcriptomic profiles of all biological replicates from each experimental group. The plot shows separation between groups based on gene expression variance along PCA 1, 2, and 3, indicating distinct transcriptional signatures (Figure S9).

Histology, immunohistochemistry, and *in situ* hybridization

The gWAT, interscapular BAT, and pancreas tissues were excised, fixed with 10% buffered formalin, and embedded in paraffin. Deparaffinized tissue slides were prepared for hematoxylin and eosin (H&E), IHC staining, and *in situ* hybridization assay. For IHC, the following primary antibodies were used: anti-UCP-1 (1:100, ab10983, Abcam); anti-FIS-1 Rabbit polyclonal antibody (1:100, 10956-1-AP, Proteintech, USA); anti-SDHB (1:200, 10620-1-A, Proteintech, USA); ani-YBX1(1:500, LS-B12352, LSBio, USA); and rabbit monoclonal antibody anti-CD31(1:50, 77699, cell signaling) and anti-F4/80 (1:100, 70076, cell signaling) and anti-CHOP (1:50, 15204-1-AP, Proteintech, USA). Secondary antibody goat anti-rabbit (1;200, BA-1000, Invitrogen). Images were taken at 40× magnification using a microscope (KEYENCE-BZ-800 series, Osaka, Japan) or Nano Zoomer S360 (Hamamatsu) scanner. Integrated density (IntDen) was measured using ImageJ software (ImageJ.win32) and data were reported as fold changes compared to control chow diets.

To confirm GapmeR accumulation in gWAT and BAT, *in situ* hybridization was performed as described by us.[20,29] Briefly, slides were stained with a TEX615 Red (Exiqon) fluorescent-labeled LNA-modified oligonucleotide probe complementary to LNA-MGC10 followed by DAPI staining for the nucleus. Images were taken at 20× magnifications using a fluorescence microscope (KEYENCE-BZ-800 series, Osaka, Japan).

Transmission electron microscope

TEM examination for mitochondrial structure in BAT collected from WT and lncMGC KO female and male mice was performed as we previously reported.[31] Briefly, fixed tissues in 2.5% (v/v) glutaraldehyde solution, were processed for TEM examination using FEI Tecnai 12 transmission electron microscope equipped with a Gatan Ultrascan 2 K charge-coupled device (CCD) camera in the City of Hope Electron Microscopy and Atomic Force Microscopy Core.

Reverse transcription-quantitative polymerase chain reaction

The RT-qPCR analysis was conducted as described previously.[29–31,78] RNA was extracted using the RNeasy Mini kit (Qiagen, Valencia, CA) and data were normalized to *Cypa* as internal control. Table S2 contains the sequences of primers used in this study.

Isolation of gWAT-derived stromal vascular fraction

The gWAT-SVF was isolated following a previously described experimental protocol, with slight modifications.[38] Briefly, a portion of isolated gWAT was collected in ice-cold base solution containing 1X-Dulbecco's phosphate-buffered saline (DPBS, without Ca and Mg) supplemented with 0.5% bovine serum albumin (BSA). Approximately, 1.2–1.5 g gWAT/sample was weighed, minced with surgical blades, and transferred into 50 mL conical tube containing digestion solution (3 mL/Sample). Digestion solution consists of 4 mg/mL collagenase, 10 mM CaCl2 in base solution. Next, gWAT homogenates were incubated at 37°C for 20 min with shaking (∼200 rpm), followed by addition of 10 mL ice-cold base solution. After triturating multiple times with a serological pipette, the cell suspension was passed through a 100 μm filter into a new 50 mL conical tube, followed by centrifugation at 500 × g (10 min, 4°C). After decanting the supernatant, the SVF cell pellet was resuspended in RBC lysis buffer to remove erythrocytes. After 5 min incubation, we added 20 mL base solution and centrifuged cell suspension at 500 × g (10 min, 4°C). Finaly, after cell counting, SVF cells were cryopreserved for further experiments.

GapmeR targeting lncMGC *in vitro* in cells

Mouse 3T3L1 pre-adipocytes were cultured in Dulbecco's modified Eagle's medium (DMEM) with 10% (v/v) bovine calf serum and 1% penicillin-streptomycin. Mouse HIB 1B brown adipocytes were cultured in DMEM supplemented with 10% (v/v) fetal bovine serum and 1% penicillin-streptomycin. Then, 3T3L1 and HIB 1B cells were transfected with 1 μmol/L lncMGC-GapmeR or NC GapmeR for 4 days by Gymnosis[31,81] and cultured in 12-well plates. For miR-379 overexpression and lncMGC GapmeR antagomir/rescue in brown adipocytes, HIB1B brown adipocytes (∼3.5×10^5 cells/transfection) were transfected with oligo mimics of miR-379 (50 nmol/L) or the corresponding NC oligos (50 nmol/L) using Lipofectamine

RNAiMAX. Cells were then treated with lncMGC-specific GapmeR (2 μmol/L) for 3 days via Gymnosis.[31]

Statistics and data analysis

Statistical analyses were conducted using GraphPad Prism software (version 10.0.0). Prior to intergroup comparisons, the normal distribution of each sample group was verified using either the χ^2 test or the Shapiro-Wilk test. For comparisons involving multiple groups, one-way or two-way analysis of variance (ANOVA) was performed as appropriate, followed by Tukey's post hoc test for multiple comparisons. Statistical comparisons between two groups were performed using two-sided Student's *t* tests. Analysis of indirect calorimetry experiments was performed using CalR software and ANCOVA test was used for statistics. X/Y Graphs show the means and standard deviation (SD). Box and whiskers graphs show Min. to Max. with all data points. To maintain figure clarity, only statistically significant *p* values ($p < 0.05$) are displayed in the graphs. All statistical tests and sample sizes are provided in the corresponding figure legends.

DATA AND CODE AVAILABILITY

The authors declare that GeoMx datasets generated and analyzed during the current study are available in the Gene Expression Omnibus (GEO) repository (accession number: GSE293681). Other supporting data are available within the article and supplemental information.

ACKNOWLEDGMENTS

We are grateful to members of the Natarajan laboratory for helpful discussions and Dr. Sung Hee Kil (Arthur Riggs Diabetes and Metabolism Research Institute, AR-DMRI) for valuable editing and insightful comments. This project was supported in part by grants and funds from the National Institutes of Health (NIH), R01 DK081705, R01 DK065073, and R01DK143577, an AR-DMRI Innovative Award at the City of Hope, and the George & Irina Schaeffer Foundation. Research reported in this publication included work performed in the following Cores: Pathology Research Services; Solid tumor and Molecular pathology, Light Microscopy/Digital Imaging, Electron Microscopy (EM/AFM), Integrative Genomics, and Comprehensive Metabolic Phenotyping, supported by the National Cancer Institute of the NIH under award number P30CA33572, as well as the Animal Resource Center, and the Transgenic/Knockout Animal Cores at City of Hope. J.N. was supported by an AR-DMRI summer student fellowship.

AUTHOR CONTRIBUTIONS

M.A. conceptualized the work, designed, and performed most experiments, generated the Figures and Tables, and wrote the manuscript. R.N. conceptualized the work, contributed to the experimental design and data interpretation, edited the manuscript, acquired funding, and supervised the study. M.K. generated the lncMGC KO mice and contributed to the experimental design. M.A., V.M., V.S.T., M.K., L.L., L.Z., A.R., L.K., and J.N. performed research. V.S.T., A.R., and L.Y. performed GeoMx data analysis and R.K.P. provided support for GeoMx profiling and analyses. K.M. provided BAT cells, and data interpretation. K.M. and W.H. provided valuable advice and edited the manuscript. All authors have read and approved the manuscript.

DECLARATION OF INTERESTS

M.K. and R.N. have a patent issued and pending patent applications through the City of Hope, disclosing and claiming certain parts of the work detailed in this manuscript. These pending applications and patents include the following two families: (1) U.S. Patent Number 10,787,664 and U.S. Patent Application No. 16/985,779 (published as U.S. Patent Application Publication no. 2020/0407721), which both claim priority to U.S. Provisional Application no. 62/166,533; and (2) U.S. Patent Application no. 17/268,068 (published as U.S. Patent Application Publication no. 2021/0310000), which claims priority to PCT/US2019/046896 and U.S. Provisional Application no. 62/719,566.

SUPPLEMENTAL INFORMATION

Supplemental information can be found online at https://doi.org/10.1016/j.omtn.2025.102792.

REFERENCES

1. Cardel, M.I., Atkinson, M.A., Taveras, E.M., Holm, J.C., and Kelly, A.S. (2020). Obesity Treatment Among Adolescents: A Review of Current Evidence and Future Directions. JAMA Pediatr. *174*, 609–617.
2. Abel, E.D., Gloyn, A.L., Evans-Molina, C., Joseph, J.J., Misra, S., Pajvani, U.B., Simcox, J., Susztak, K., and Drucker, D.J. (2024). Diabetes mellitus—Progress and opportunities in the evolving epidemic. Cell *187*, 3789–3820.
3. Stenvinkel, P., Zoccali, C., and Ikizler, T.A. (2013). Obesity in CKD–what should nephrologists know? J. Am. Soc. Nephrol. *24*, 1727–1736.
4. Lega, I.C., and Lipscombe, L.L. (2020). Review: Diabetes, Obesity, and Cancer-Pathophysiology and Clinical Implications. Endocr. Rev. *41*, bnz014.
5. Fitzgerald, S.J., Janorkar, A.V., Barnes, A., and Maranon, R.O. (2018). A new approach to study the sex differences in adipose tissue. J. Biomed. Sci. *25*, 89.
6. Terrazas, S., Brashear, L., Escoto, A.-K., Lynch, S., Slaughter, D., Xavier, N., Estes, I.I.,N.R., and Giordano-Mooga, S. (2019). Sex Differences in Obesity-Induced Inflammation (Translational Studies on Inflammation).
7. Jensterle, M., Rizzo, M., Haluzík, M., and Janež, A. (2022). Efficacy of GLP-1 RA Approved for Weight Management in Patients With or Without Diabetes: A Narrative Review. Adv. Ther. *39*, 2452–2467.
8. Sidik, S. (2023). Beyond Ozempic: brand-new obesity drugs will be cheaper and more effective. Nature *619*, 19.
9. Lenharo, M. (2023). Game-changing obesity drugs go mainstream: what scientists are learning. Nature *618*, 17–18.
10. Chakhtoura, M., Haber, R., Ghezzawi, M., Rhayem, C., Tcheroyan, R., and Mantzoros, C.S. (2023). Pharmacotherapy of obesity: an update on the available medications and drugs under investigation. eClinicalMedicine *58*, 101882.
11. Nemeth, K., Bayraktar, R., Ferracin, M., and Calin, G.A. (2024). Non-coding RNAs in disease: from mechanisms to therapeutics. Nat. Rev. Genet. *25*, 211–232.
12. Cabili, M.N., Trapnell, C., Goff, L., Koziol, M., Tazon-Vega, B., Regev, A., and Rinn, J.L. (2011). Integrative annotation of human large intergenic noncoding RNAs reveals global properties and specific subclasses. Genes Dev. *25*, 1915–1927.
13. Li, P., Chen, X., Chang, X., Tang, T., and Qi, K. (2020). A preliminary study on the differential expression of long noncoding RNAs and messenger RNAs in obese and control mice. J. Cell. Biochem. *121*, 1126–1143.
14. Kato, M., and Natarajan, R. (2019). Epigenetics and epigenomics in diabetic kidney disease and metabolic memory. Nat. Rev. Nephrol. *15*, 327–345.
15. Shang, R., Lee, S., Senavirathne, G., and Lai, E.C. (2023). microRNAs in action: biogenesis, function and regulation. Nat. Rev. Genet. *24*, 816–833.
16. Deshpande, S., Abdollahi, M., Wang, M., Lanting, L., Kato, M., and Natarajan, R. (2018). Reduced Autophagy by a microRNA-mediated Signaling Cascade in Diabetes-induced Renal Glomerular Hypertrophy. Sci. Rep. *8*, 6954.
17. Deshpande, S.D., Putta, S., Wang, M., Lai, J.Y., Bitzer, M., Nelson, R.G., Lanting, L.L., Kato, M., and Natarajan, R. (2013). Transforming growth factor-β-induced cross talk between p53 and a microRNA in the pathogenesis of diabetic nephropathy. Diabetes *62*, 3151–3162.
18. Kato, M., Abdollahi, M., Tunduguru, R., Tsark, W., Chen, Z., Wu, X., Wang, J., Chen, Z.B., Lin, F.M., Lanting, L., et al. (2021). miR-379 deletion ameliorates features of diabetic kidney disease by enhancing adaptive mitophagy via FIS1. Commun. Biol. *4*, 30.
19. Kato, M., Putta, S., Wang, M., Yuan, H., Lanting, L., Nair, I., Gunn, A., Nakagawa, Y., Shimano, H., Todorov, I., et al. (2009). TGF-beta activates Akt kinase through a microRNA-dependent amplifying circuit targeting PTEN. Nat. Cell Biol. *11*, 881–889.
20. Kato, M., Wang, M., Chen, Z., Bhatt, K., Oh, H.J., Lanting, L., Deshpande, S., Jia, Y., Lai, J.Y.C., O'Connor, C.L., et al. (2016). An endoplasmic reticulum stress-regulated lncRNA hosting a microRNA megacluster induces early features of diabetic nephropathy. Nat. Commun. *7*, 12864.

21. Das, S., Reddy, M.A., Senapati, P., Stapleton, K., Lanting, L., Wang, M., Amaram, V., Ganguly, R., Zhang, L., Devaraj, S., et al. (2018). Diabetes Mellitus-Induced Long Noncoding RNA Dnm3os Regulates Macrophage Functions and Inflammation via Nuclear Mechanisms. Arterioscler. Thromb. Vasc. Biol. *38*, 1806–1820.

22. Das, S., Senapati, P., Chen, Z., Reddy, M.A., Ganguly, R., Lanting, L., Mandi, V., Bansal, A., Leung, A., Zhang, S., et al. (2017). Regulation of angiotensin II actions by enhancers and super-enhancers in vascular smooth muscle cells. Nat. Commun. *8*, 1467.

23. Das, S., Zhang, E., Senapati, P., Amaram, V., Reddy, M.A., Stapleton, K., Leung, A., Lanting, L., Wang, M., Chen, Z., et al. (2018). A Novel Angiotensin II-Induced Long Noncoding RNA Giver Regulates Oxidative Stress, Inflammation, and Proliferation in Vascular Smooth Muscle Cells. Circ. Res. *123*, 1298–1312.

24. Reddy, M.A., Amaram, V., Das, S., Tanwar, V.S., Ganguly, R., Wang, M., Lanting, L., Zhang, L., Abdollahi, M., Chen, Z., et al. (2021). lncRNA DRAIR is downregulated in diabetic monocytes and modulates the inflammatory phenotype via epigenetic mechanisms. JCI Insight *6*, e143289.

25. Stapleton, K., Das, S., Reddy, M.A., Leung, A., Amaram, V., Lanting, L., Chen, Z., Zhang, L., Palanivel, R., Deiuliis, J.A., and Natarajan, R. (2020). Novel Long Noncoding RNA, Macrophage Inflammation-Suppressing Transcript (MIST), Regulates Macrophage Activation During Obesity. Arterioscler. Thromb. Vasc. Biol. *40*, 914–928.

26. Tanwar, V.S., Reddy, M.A., Das, S., Samara, V.A., Abdollahi, M., Dey, S., Malek, V., Ganguly, R., Stapleton, K., Lanting, L., et al. (2023). Palmitic acid–induced long noncoding RNA PARAIL regulates inflammation via interaction with RNA-binding protein ELAVL1 in monocytes and macrophages. Arterioscler. Thromb. Vasc. Biol. *43*, 1157–1175.

27. Kato, M., Chen, Z., Das, S., Wu, X., Wang, J., Li, A., Chen, W., Tsark, W., Tunduguru, R., Lanting, L., et al. (2023). Long non-coding RNA lncMGC mediates the expression of TGF-beta-induced genes in renal cells via nucleosome remodelers. Front. Mol. Biosci. *10*, 1204124.

28. Tanwar, V.S., Reddy, M.A., and Natarajan, R. (2021). Emerging Role of Long Non-Coding RNAs in Diabetic Vascular Complications. Front. Endocrinol. *12*, 665811.

29. Kato, M., Abdollahi, M., Omori, K., Malek, V., Lanting, L., Kandeel, F., Rawson, J., Tsark, W., Zhang, L., and Wang, M. (2024). Lowering an ER-stress-regulated long noncoding RNA protects mice from diabetes and isolated pancreatic islets from cell death. Molecular Therapy-Nucleic Acids *35*, 102252.

30. Abdollahi, M., Kato, M., Lanting, L., Wang, M., Tunduguru, R., and Natarajan, R. (2022). Role of miR-379 in high-fat diet-induced kidney injury and dysfunction. Am. J. Physiol. Renal Physiol. *323*, F686–F699.

31. Abdollahi, M., Kato, M., Lanting, L., Tunduguru, R., Wang, M., Wang, Y., Fueger, P.T., Wang, Q., Huang, W., and Natarajan, R. (2022). miR-379 mediates insulin resistance and obesity through impaired angiogenesis and adipogenesis regulated by ER stress. Mol. Ther. Nucleic Acids *30*, 115–130.

32. Zhong, H., Ma, M., Liang, T., and Guo, L. (2018). Role of MicroRNAs in Obesity-Induced Metabolic Disorder and Immune Response. J. Immunol. Res. *2018*, 2835761.

33. Lorente-Cebrián, S., González-Muniesa, P., Milagro, F.I., and Martínez, J.A. (2019). MicroRNAs and other non-coding RNAs in adipose tissue and obesity: emerging roles as biomarkers and therapeutic targets. Clin. Sci. *133*, 23–40.

34. Landrier, J.-F., Derghal, A., and Mounien, L. (2019). MicroRNAs in obesity and related metabolic disorders. Cells *8*, 859.

35. Khalili, D., Khayamzadeh, M., Kohansal, K., Ahanchi, N.S., Hasheminia, M., Hadaegh, F., Tohidi, M., Azizi, F., and Habibi-Moeini, A.S. (2023). Are HOMA-IR and HOMA-B good predictors for diabetes and pre-diabetes subtypes? BMC Endocr. Disord. *23*, 39.

36. Guo, S. (2014). Insulin signaling, resistance, and the metabolic syndrome: insights from mouse models into disease mechanisms. J. Endocrinol. *220*, T1–T23.

37. Herold, J., and Kalucka, J. (2020). Angiogenesis in Adipose Tissue: The Interplay Between Adipose and Endothelial Cells. Front. Physiol. *11*, 624903.

38. Orr, J.S., Kennedy, A.J., and Hasty, A.H. (2013). Isolation of adipose tissue immune cells. JoVE J *75*, e50707.

39. Ramakrishnan, V.M., and Boyd, N.L. (2018). The Adipose Stromal Vascular Fraction as a Complex Cellular Source for Tissue Engineering Applications. Tissue Eng. Part B Rev. *24*, 289–299.

40. Wu, H., Dixon, E.E., Xuanyuan, Q., Guo, J., Yoshimura, Y., Debashish, C., Niesnerova, A., Xu, H., Rouault, M., and Humphreys, B.D. (2024). High resolution spatial profiling of kidney injury and repair using RNA hybridization-based in situ sequencing. Nat. Commun. *15*, 1396.

41. Shinde, A.B., Song, A., and Wang, Q.A. (2021). Brown Adipose Tissue Heterogeneity, Energy Metabolism, and Beyond. Front. Endocrinol. *12*, 651763.

42. Wang, X., Yu, H., Gao, R., Liu, M., and Xie, W. (2023). A comprehensive review of the family of very-long-chain fatty acid elongases: structure, function, and implications in physiology and pathology. Eur. J. Med. Res. *28*, 532.

43. Kadenbach, B. (2021). Complex IV - The regulatory center of mitochondrial oxidative phosphorylation. Mitochondrion *58*, 296–302.

44. Tseng, Y.-H., Kriauciunas, K.M., Kokkotou, E., and Kahn, C.R. (2004). Differential roles of insulin receptor substrates in brown adipocyte differentiation. Mol. Cell Biol. *24*, 1918–1929.

45. Kim, G., Lee, J., Ha, J., Kang, I., and Choe, W. (2023). Endoplasmic Reticulum Stress and Its Impact on Adipogenesis: Molecular Mechanisms Implicated. Nutrients *15*, 5082.

46. Tripathi, Y.B., and Pandey, V. (2012). Obesity and endoplasmic reticulum (ER) stresses. Front. Immunol. *3*, 240.

47. Wang, Y., Vera, L., Fischer, W.H., and Montminy, M. (2009). The CREB coactivator CRTC2 links hepatic ER stress and fasting gluconeogenesis. Nature *460*, 534–537.

48. Fu, M., Rao, M., Bouras, T., Wang, C., Wu, K., Zhang, X., Li, Z., Yao, T.P., and Pestell, R.G. (2005). Cyclin D1 inhibits peroxisome proliferator-activated receptor gamma-mediated adipogenesis through histone deacetylase recruitment. J. Biol. Chem. *280*, 16934–16941.

49. Lee, J.M., Choi, S.S., Lee, Y.H., Khim, K.W., Yoon, S., Kim, B.G., Nam, D., Suh, P.G., Myung, K., and Choi, J.H. (2018). The E3 ubiquitin ligase TRIM25 regulates adipocyte differentiation via proteasome-mediated degradation of PPARγ. Exp. Mol. Med. *50*, 1–11.

50. Wu, R., Cao, S., Li, F., Feng, S., Shu, G., Wang, L., Gao, P., Zhu, X., Zhu, C., Wang, S., and Jiang, Q. (2022). RNA-binding protein YBX1 promotes brown adipogenesis and thermogenesis via PINK1/PRKN-mediated mitophagy. Faseb j *36*, e22219.

51. Lu, T.X., and Rothenberg, M.E. (2018). MicroRNA. J. Allergy Clin. Immunol. *141*, 1202–1207.

52. Fujii, M., Setoyama, D., Gotoh, K., Dozono, Y., Yagi, M., Ikeda, M., Ide, T., Uchiumi, T., and Kang, D. (2022). TFAM expression in brown adipocytes confers obesity resistance by secreting extracellular vesicles that promote self-activation. iScience *25*, 104889.

53. Ko, M.S., Yun, J.Y., Baek, I.-J., Jang, J.E., Hwang, J.J., Lee, S.E., Heo, S.-H., Bader, D.A., Lee, C.-H., Han, J., et al. (2021). Mitophagy deficiency increases NLRP3 to induce brown fat dysfunction in mice. Autophagy *17*, 1205–1221.

54. Putta, S., Lanting, L., Sun, G., Lawson, G., Kato, M., and Natarajan, R. (2012). Inhibiting microRNA-192 ameliorates renal fibrosis in diabetic nephropathy. J. Am. Soc. Nephrol. *23*, 458–469.

55. Cornejo, K.M., Lu, M., Yang, P., Wu, S., Cai, C., Zhong, W.d., Olumi, A., Young, R.H., and Wu, C.L. (2015). Succinate dehydrogenase B: a new prognostic biomarker in clear cell renal cell carcinoma. Hum. Pathol. *46*, 820–826.

56. Casimiro, I., Stull, N.D., Tersey, S.A., and Mirmira, R.G. (2021). Phenotypic sexual dimorphism in response to dietary fat manipulation in C57BL/6J mice. J. Diabetes Complications *35*, 107795.

57. Becker, J.B., and Ahmed, S.B. (2025). Sex differences research is important. Biol. Sex Differ. *16*, 20.

58. Stapleton, S., Welch, G., DiBerardo, L., and Freeman, L.R. (2024). Sex differences in a mouse model of diet-induced obesity: the role of the gut microbiome. Biol. Sex Differ. *15*, 5.

59. Koceva, A., Herman, R., Janez, A., Rakusa, M., and Jensterle, M. (2024). Sex- and Gender-Related Differences in Obesity: From Pathophysiological Mechanisms to Clinical Implications. Int. J. Mol. Sci. *25*, 7342.

60. Chlamydas, S., Markouli, M., Strepkos, D., and Piperi, C. (2022). Epigenetic mechanisms regulate sex-specific bias in disease manifestations. J. Mol. Med. *100*, 1111–1123.

61. Carpentier, A.C., Blondin, D.P., Haman, F., and Richard, D. (2023). Brown adipose tissue—a translational perspective. Endocr. Rev. *44*, 143–192.

62. Rustin, P., Munnich, A., and Rötig, A. (2002). Succinate dehydrogenase and human diseases: new insights into a well-known enzyme. Eur. J. Hum. Genet. *10*, 289–291.

63. Vernochet, C., Damilano, F., Mourier, A., Bezy, O., Mori, M.A., Smyth, G., Rosenzweig, A., Larsson, N.G., and Kahn, C.R. (2014). Adipose tissue mitochondrial dysfunction triggers a lipodystrophic syndrome with insulin resistance, hepatosteatosis, and cardiovascular complications. Faseb j *28*, 4408–4419.

64. Li, H., Yu, L., and Zhao, C. (2019). Dioscin attenuates high-fat diet-induced insulin resistance of adipose tissue through the IRS-1/PI3K/Akt signaling pathway. Mol. Med. Rep. *19*, 1230–1237.

65. Cignarelli, A., Genchi, V.A., Perrini, S., Natalicchio, A., Laviola, L., and Giorgino, F. (2019). Insulin and insulin receptors in adipose tissue development. Int. J. Mol. Sci. *20*, 759.

66. Chandrasekaran, P., and Weiskirchen, R. (2024). Cellular and molecular mechanisms of insulin resistance. Curr. Tissue Microenviron. Rep. *5*, 79–90.

67. Chawla, B., Hedman, A.C., Sayedyahossein, S., Erdemir, H.H., Li, Z., and Sacks, D.B. (2017). Absence of IQGAP1 Protein Leads to Insulin Resistance. J. Biol. Chem. *292*, 3273–3289.

68. Recabarren, D., and Alarcón, M. (2017). Gene networks in neurodegenerative disorders. Life Sci. *183*, 83–97.

69. Henriques, B.J., Katrine Jentoft Olsen, R., Gomes, C.M., and Bross, P. (2021). Electron transfer flavoprotein and its role in mitochondrial energy metabolism in health and disease. Gene *776*, 145407.

70. Barel, O., Shorer, Z., Flusser, H., Ofir, R., Narkis, G., Finer, G., Shalev, H., Nasasra, A., Saada, A., and Birk, O.S. (2008). Mitochondrial complex III deficiency associated with a homozygous mutation in UQCRQ. Am. J. Hum. Genet. *82*, 1211–1216.

71. Sepa-Kishi, D.M., Jani, S., Da Eira, D., and Ceddia, R.B. (2019). Cold acclimation enhances UCP1 content, lipolysis, and triacylglycerol resynthesis, but not mitochondrial uncoupling and fat oxidation, in rat white adipocytes. Am. J. Physiol. Cell Physiol. *316*, C365–C376.

72. Liu, Y., Qian, S.-W., Tang, Y., and Tang, Q.-Q. (2024). The secretory function of adipose tissues in metabolic regulation. Life Metab. *3*, loae003.

73. Garritson, J.D., and Boudina, S. (2021). The Effects of Exercise on White and Brown Adipose Tissue Cellularity, Metabolic Activity and Remodeling. Front. Physiol. *12*, 772894.

74. Memme, J.M., Erlich, A.T., Phukan, G., and Hood, D.A. (2021). Exercise and mitochondrial health. J. Physiol. *599*, 803–817.

75. Secher, A., Jelsing, J., Baquero, A.F., Hecksher-Sørensen, J., Cowley, M.A., Dalbøge, L.S., Hansen, G., Grove, K.L., Pyke, C., Raun, K., et al. (2014). The arcuate nucleus mediates GLP-1 receptor agonist liraglutide-dependent weight loss. J. Clin. Investig. *124*, 4473–4488.

76. Chen, X.Y., Chen, L., Yang, W., and Xie, A.M. (2021). GLP-1 Suppresses Feeding Behaviors and Modulates Neuronal Electrophysiological Properties in Multiple Brain Regions. Front. Mol. Neurosci. *14*, 793004.

77. Drucker, D.J. (2025). GLP-1-based therapies for diabetes, obesity and beyond. Nat. Rev. Drug Discov. *24*, 631–650.

78. Kato, M., Chen, Z., Das, S., Wu, X., Wang, J., Li, A., Chen, W., Tsark, W., Tunduguru, R., Lanting, L., et al. (2023). Long non-coding RNA lncMGC mediates the expression of TGF-β-induced genes in renal cells via nucleosome remodelers. Front. Mol. Biosci. *10*, 1204124.

79. Guo, A., Daniels, N.A., Thuma, J., McCall, K.D., Malgor, R., and Schwartz, F.L. (2015). Diet is critical for prolonged glycemic control after short-term insulin treatment in high-fat diet-induced type 2 diabetic male mice. PLoS One *10*, e0117556.

80. Fraulob, J.C., Ogg-Diamantino, R., Fernandes-Santos, C., Aguila, M.B., and Mandarim-de-Lacerda, C.A. (2010). A Mouse Model of Metabolic Syndrome: Insulin Resistance, Fatty Liver and Non-Alcoholic Fatty Pancreas Disease (NAFPD) in C57BL/6 Mice Fed a High Fat Diet. J. Clin. Biochem. Nutr. *46*, 212–223.

81. Stein, C.A., Hansen, J.B., Lai, J., Wu, S., Voskresenskiy, A., Høg, A., Worm, J., Hedtjärn, M., Souleimanian, N., Miller, P., et al. (2010). Efficient gene silencing by delivery of locked nucleic acid antisense oligonucleotides, unassisted by transfection reagents. Nucleic Acids Res. *38*, e3.

OMTN, Volume *37*

Supplemental information

Metabolic beneficial effects of targeting

a long non-coding RNA,

lnc-megacluster, in obesity

Maryam Abdollahi, Vajir Malek, Vinay Singh Tanwar, Mitsuo Kato, Linda Lanting, Alireza Rezaei, Lingxiao Zhang, Lixin Yang, Raju K. Pillai, Leah Kebrom, Jathan Nandi, Wendong Huang, Ke Ma, and Rama Natarajan

A

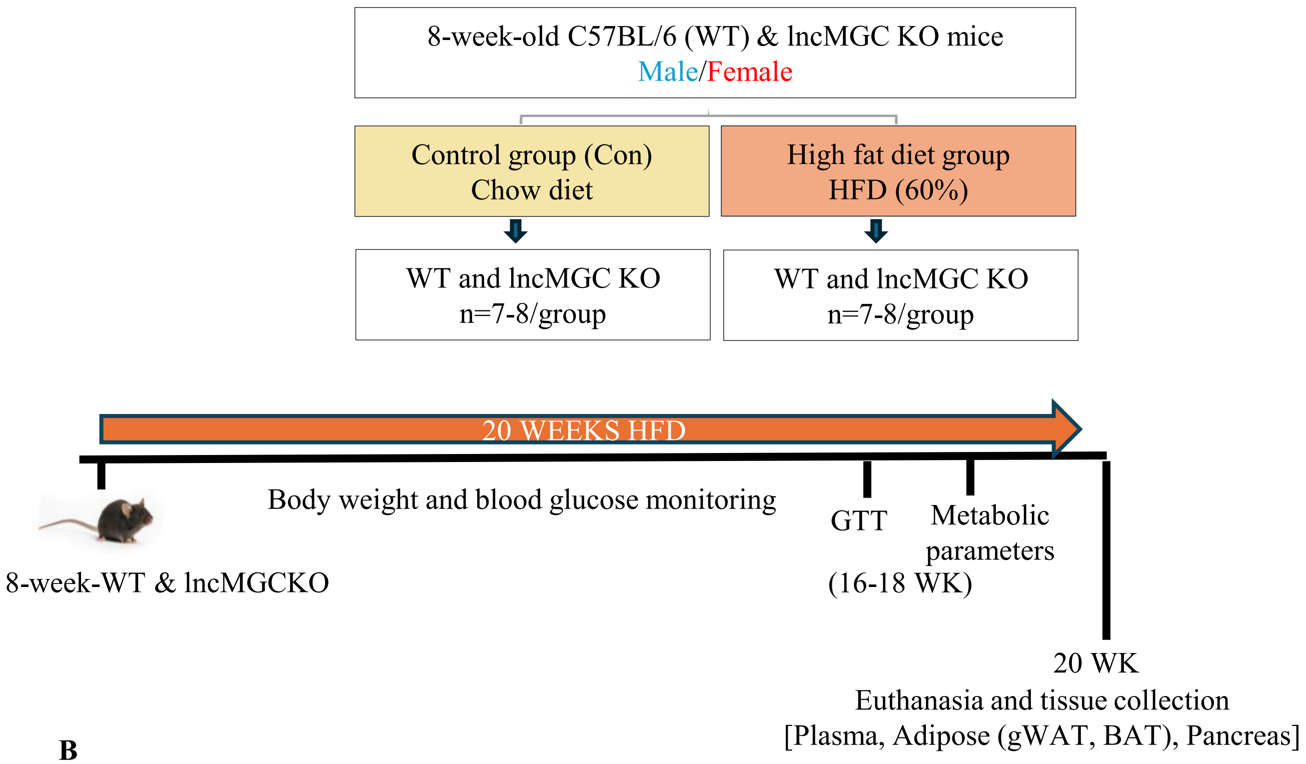

B

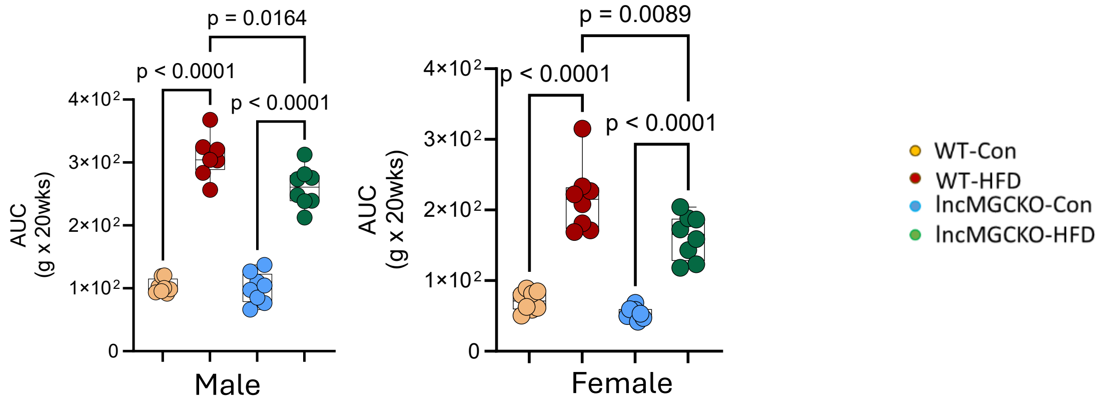

Figure S1. Experimental scheme for tracking high-fat diet (HFD) fed induced- obesity in WT and lncMGC KO mice. (A) Eight-week-old female and male wild type (WT) C57BL/6J mice and lncMGC (KO) mice were randomly divided into groups and fed with laboratory control chow-diet (Con) or high-fat diet (HFD) (60% kcal from fat) for 20 weeks. Body weights and blood glucose levels were monitored biweekly. Glucose tolerance tests (GTT) were performed, and metabolic parameters, including body composition, food and water intake, movement, oxygen consumption, and energy expenditure, were measured. Total HFD period was 20 weeks, but metabolic parameters and GTT were measured at 16-18 weeks of HFD. After euthanasia, plasma, perigonadal white adipose tissue (gWAT), interscapular brown adipose tissue (BAT), and pancreata were harvested. (B) Body weight gain was calculated as the increase from initial body weight using the formula:[(Final body weight – Initial body weight) / Initial body weight], and the area under the curve (AUC) was shown in the graphs. Statistical analyses were performed by Two-way ANOVA with post-hoc Tukey test for multiple comparisons. The bar and whisker plot displays the distribution of the data. The whiskers extend from the minimum to the maximum values. Individual data points are overlaid as dots. Statistically significant p-values are indicated in the bar graphs.

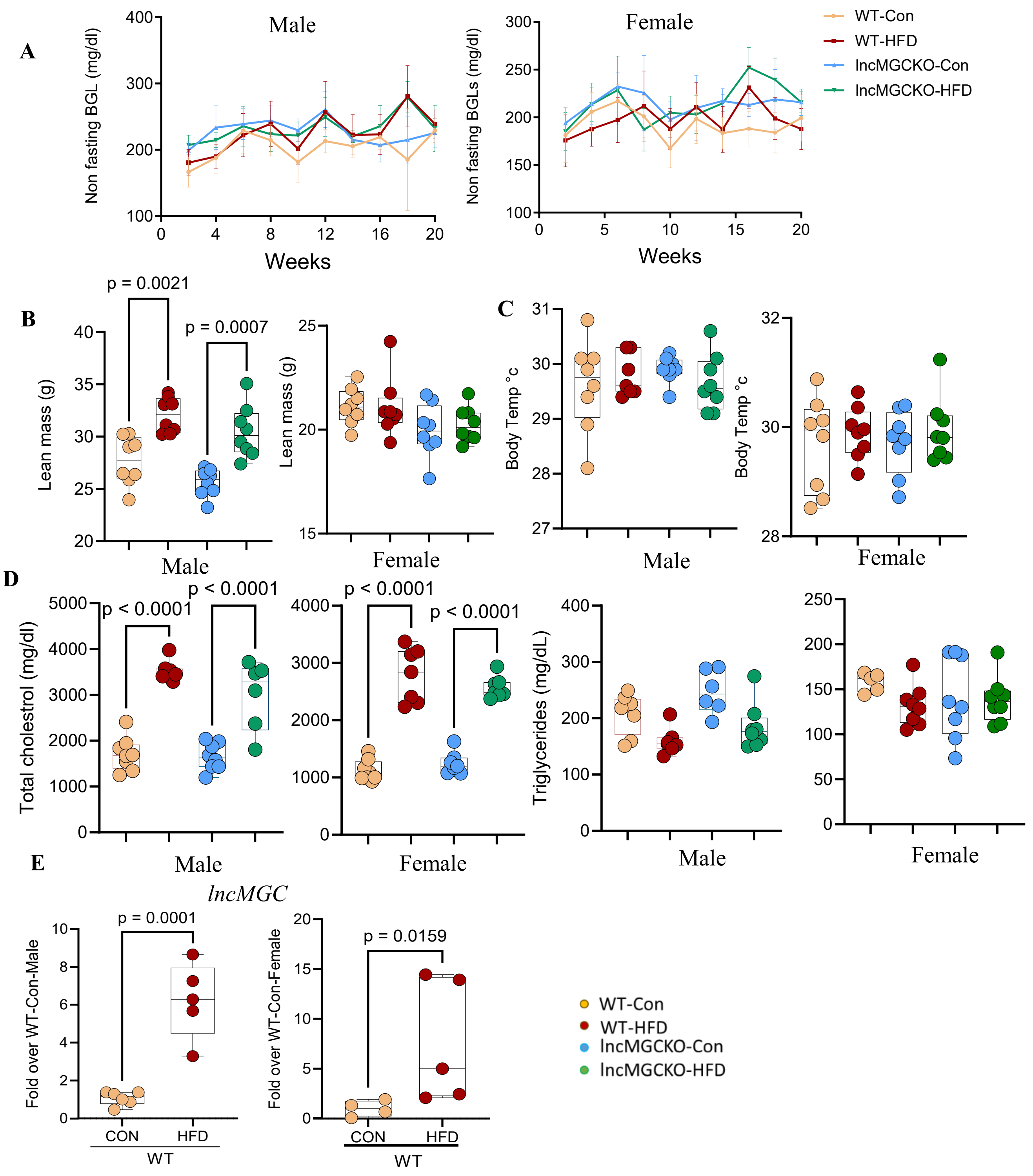

Figure S2. Monitoring blood glucose and other parameters. (A) Average non-fasting blood glucose levels (BGLs) of male and female mice over 20 weeks of diets. (B) Lean mass in male and female mice at 18 weeks of diets. (C) peripheral body temperature. (D) Plasma total cholesterol and triglyceride. n=7-8/group. (E) Gene expression of lncMGC in isolated stromal vascular fraction (SVF) from gWAT in WT and HFD male and female mice (n=4-6/group). Statistical analyses were performed by Two-way ANOVA with post-hoc Tukey test for multiple comparisons. XY graphs show the mean (SD). The bar and whisker plot displays the distribution of the data. The whiskers extend from the minimum to the maximum values. Individual data points are overlaid as dots. Statistically significant p-values are indicated in the bar graphs.

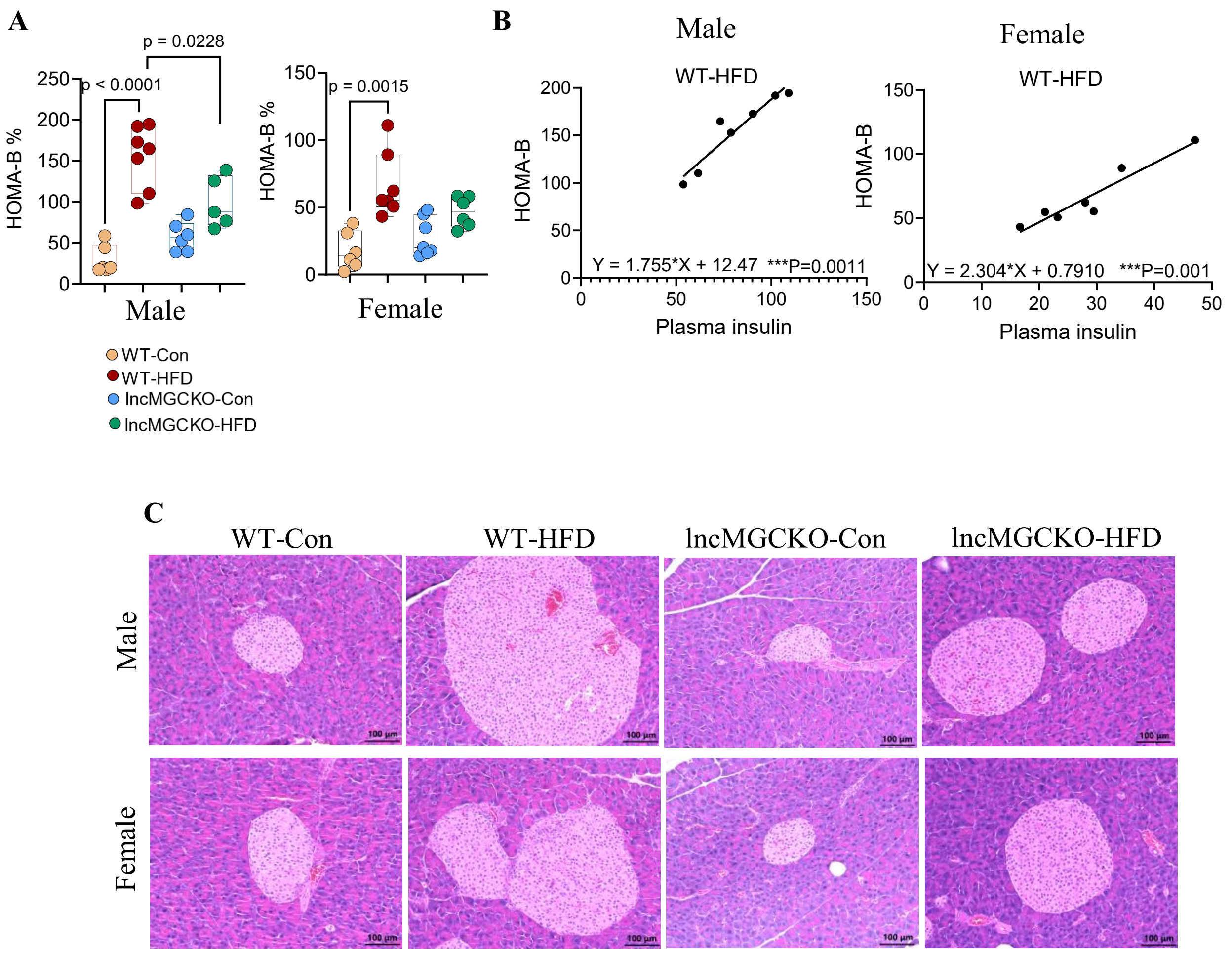

Figure S3. HFD-induced beta cell dysfunction and islet hypertrophy were improved in lncMGC KO mice. (A) Percentage of Homeostasis Model Assessment (HOMA) analysis for β-cell function (HOMA-B) in WT and lncMGC KO Con and HFD mice; (B) Correlation between beta cell function (HOMA B%) and plasma insulin levels in wild type (WT) HFD male and female mice. (C) Representative H &E staining images show islet hyperplasia and hypertrophy in WT HFD mice, which is improved in lncMGC KO HFD mice (male and female). Scale bars, 50 µm. n=5-8/group. Statistical analyses were performed by Two-way ANOVA with post-hoc Tukey test for multiple comparisons. The bar and whisker plot displays the distribution of the data. The whiskers extend from the minimum to the maximum values. Individual data points are overlaid as dots. Statistically significant p-values are indicated in the bar graphs.

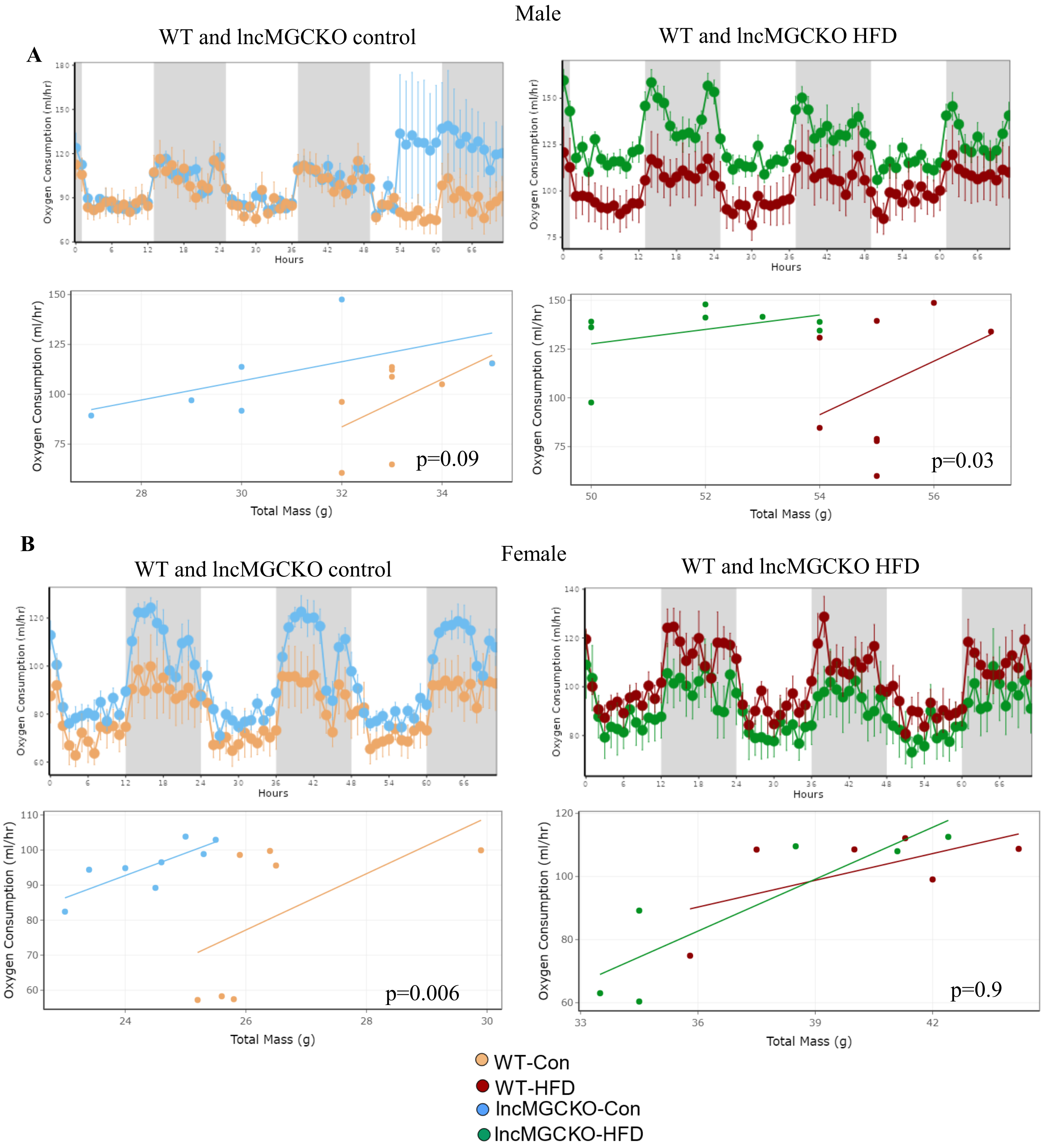

Figure S4. lncMGC KO mice exhibit increased oxygen consumption. Oxygen consumption (VO2) and regression analysis of VO2 and total body mass in WT and lncMGC KO Con, and WT and lncMGC KO HFD (A) male and (B) female mice. n=6-8. Data were analyzed for VO2 and total mass regression using ANCOVA with VO2 as a dependent variable, genotype as fixed variable, and body mass as a covariate. Night-time (gray-shades). P-values are indicated in the figures.

A

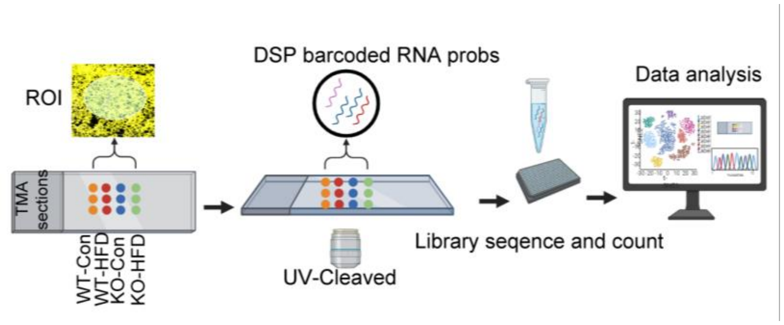

B

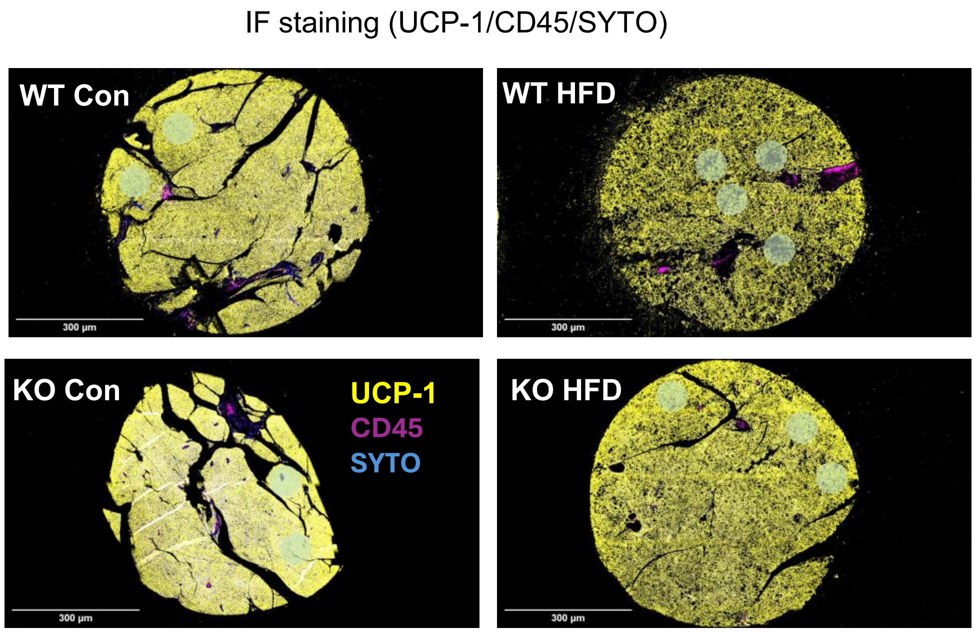

Figure S5. Nanostring Digital gene expression profiling of BAT tissues. Schematic illustration of the workflow of the Nanostring GeoMx Digital Spatial Profiler (DSP) Whole Transcriptome Atlas (WTA), which was used for the interrogation of multiple RNA analytes from a single paraffin-embedded brown adipose tissue (BAT) section from the mice groups. (B) Representative IF staining images of Tissue Microarray (TMA) sections from BAT isolated from WT and lncMGC KO Con and HFD female mice. 20 weeks HFD. The sections were stained with UCP-1(mitochondrial marker, yellow signals), CD45 (leukocytes marker, purple signals), and SYTO (nucleic acid stain, blue signal) for the selection of regions of interest (ROIs, bubble plots). Analytes in the TMA sections are conjugated with oligonucleotide (oligo) tags using a photocleavable linker. Spatially mapped UV illumination enables the release of oligo tags from the analyte into the plates. Sequencing was performed to obtain quantitative counts per ROI. Scale bar: 300 µm. N=3 mice/group. The number of selected ROIs is as follows: n=4/WT-Con, n=6\WT-HFD, n=5/lncMGC KO Con, and n=7/lncMGC KO HFD.

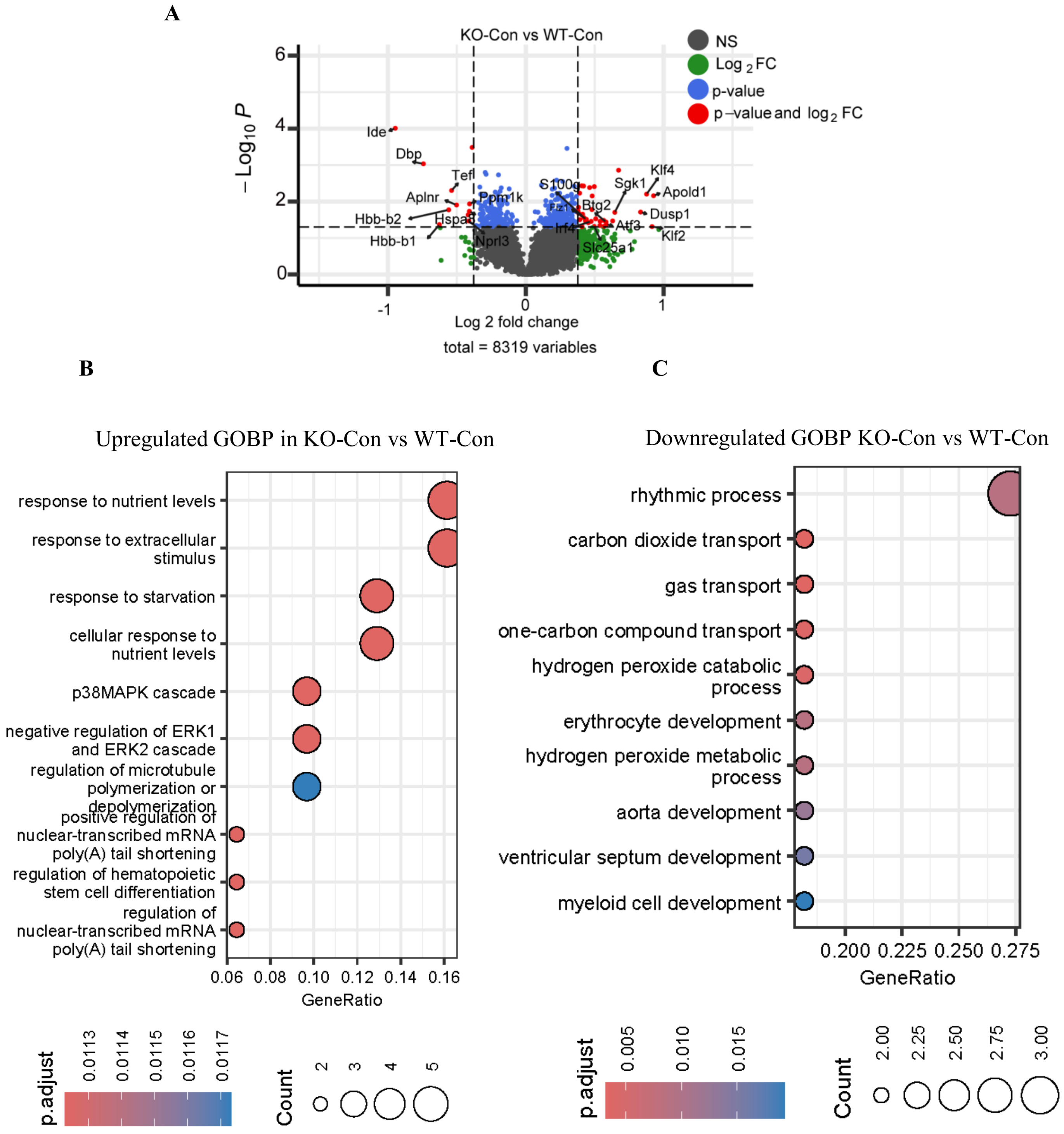

Figure S6. Gene ontology biological process in brown adipose tissue from control chow diet female mice. (A) Differential gene expression in lncMGC KO Con compared to WT Con female mice. (B)Top enriched up-regulated gene ontology biological process (GOBP) in lncMGC KO Con compared to WT Con and (C) Top enriched down-regulated GOBP in lncMGC KO Con compared to WT Con female mice. p-value<0.05.

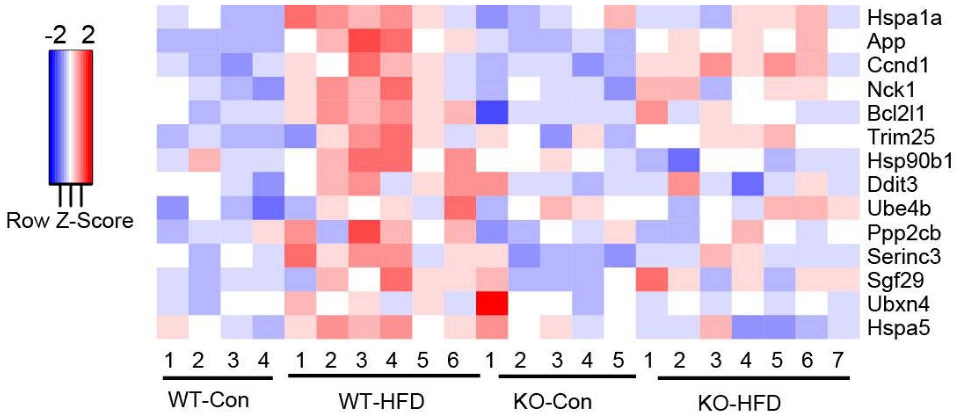

Figure S7. Factors related to endoplasmic reticulum stress are improved in brown adipose tissue of lncMGC KO HFD female mice. Heatmap showing key genes related to endoplasmic reticulum (ER) stress were significantly modulated between regions of interest (ROIs) in UCP-1 positive segments across all groups; normalized Q3 values were used to generate the heatmap. WT-Con=4 ROI, WT-HFD=6 ROI, lncMGC Con=5 ROI, lncMGCKO HFD=7 ROI. Data analysis was performed using R. Log2 fold change ±0.378 and p-value<0.05. The columns of the heatmap represent indicated genes, showing markedly altered expression levels as indicated by fold change.

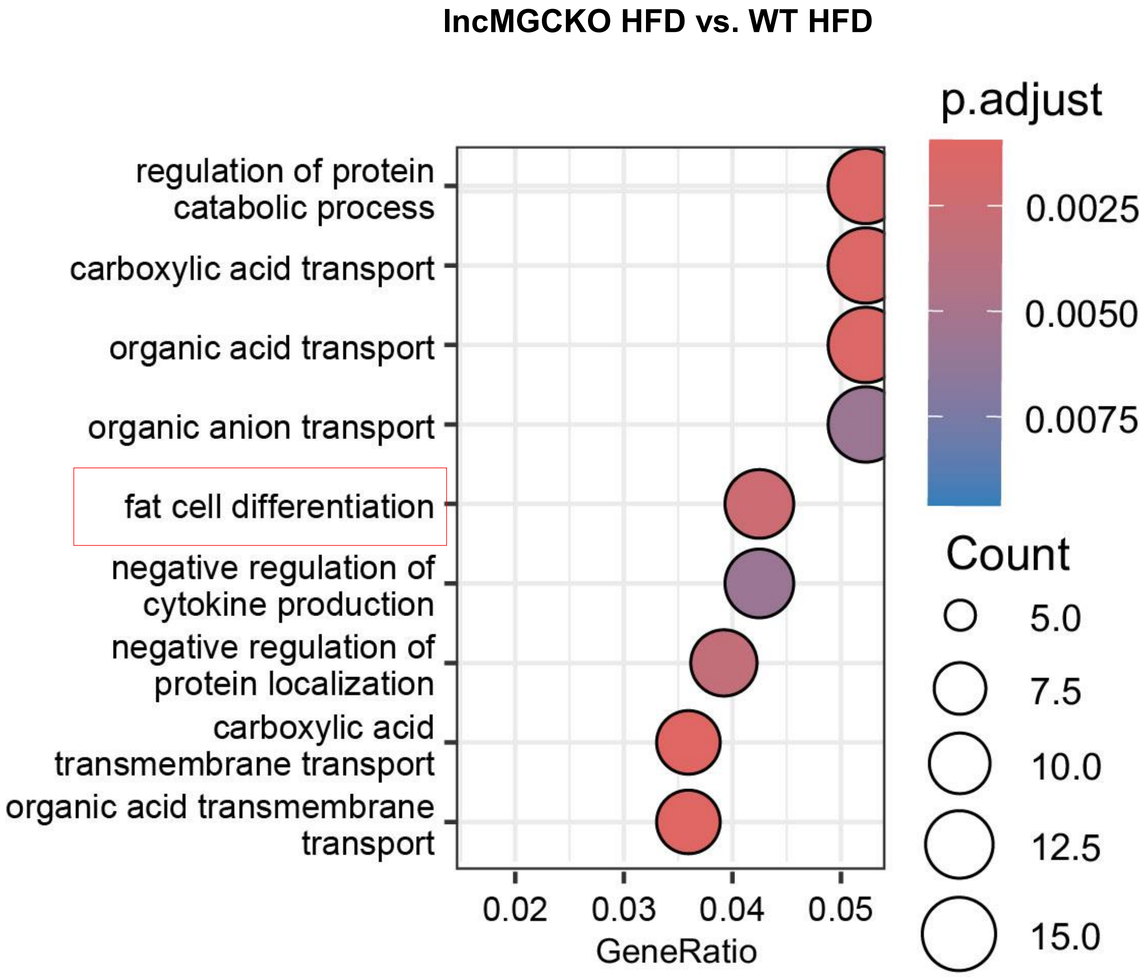

Figure S8. Gene ontology biological process in brown adipose tissue from WT and lncMGCKO HFD female mice. Top enriched up-regulated gene ontology biological process (GOBP) in lncMGC KO HFD compared to WT HFD. p-value<0.05.

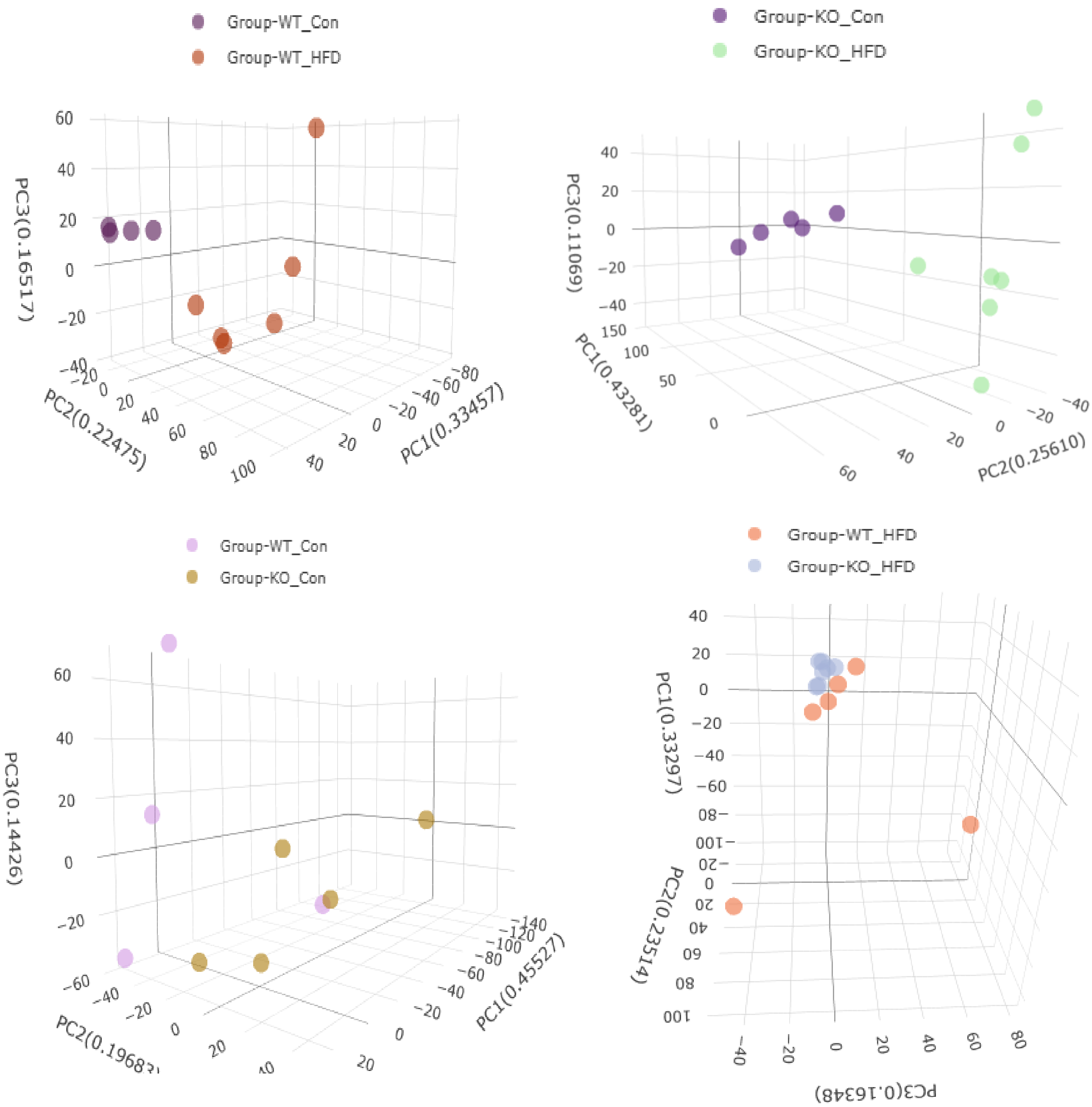

Figure S9. Principal component analysis (PCA) of RNA-seq data from brown adipose tissue. PCA was performed on the transcriptomic profiles of all biological replicates from each experimental group. The plot shows separation between groups based on gene expression variance along PCA 1,2, and 3, indicating distinct transcriptional signatures. Wild type (WT) control (Con), WT-high fat diet (HFD), lncMGCKO Con, lncMGCKO HFD

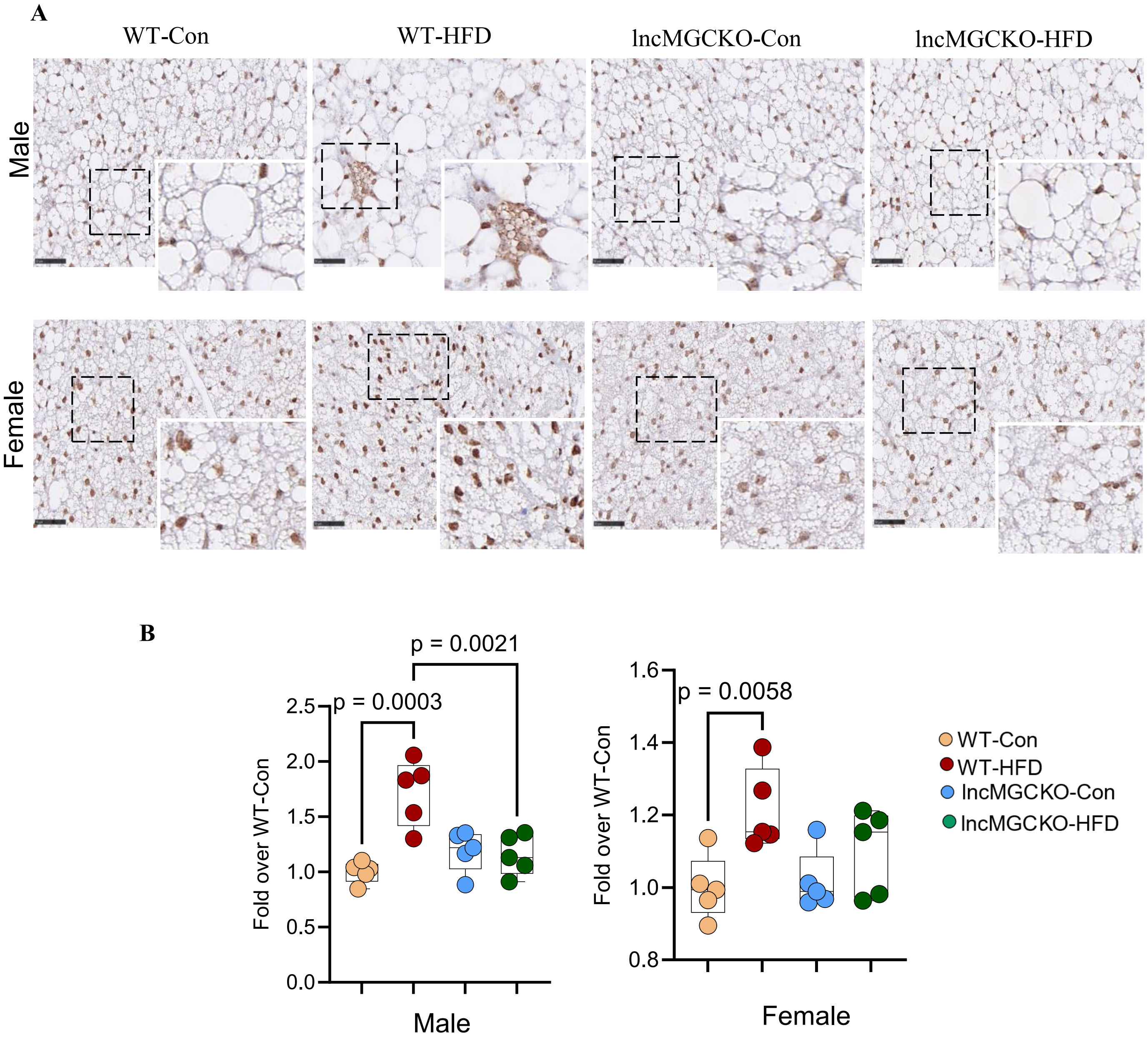

Figure S10. lncMGC KO improved expression of ER stress marker CHOP in brown adipose tissue of HFD mice. (A) Immunohistochemistry staining and (B) quantitative analysis of CHOP in BAT in male and female mice. 20 weeks of HFD. n=5/group. Statistical analyses were performed by Two-way ANOVA with post-hoc Tukey test for multiple comparisons. The bar and whisker plot displays the distribution of the data. The whiskers extend from the minimum to the maximum values. Individual data points are overlaid as dots. Statistically significant p-values are indicated in the bar graphs. The magnification indicated by the lines in the IHC images represents the selected area that has been enlarged and shown at a higher resolution.

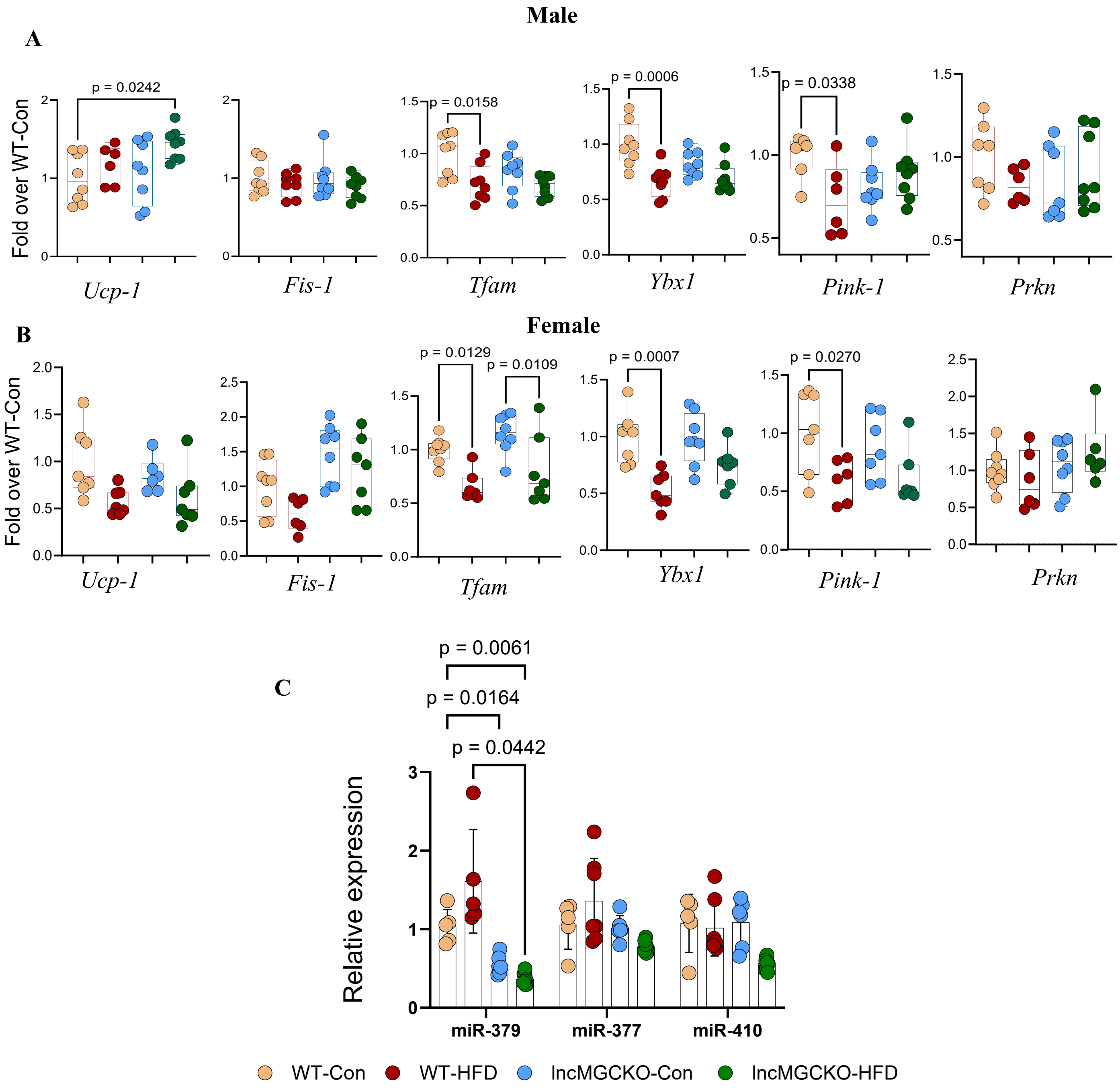

Figure S11. Gene expression of mitochondrial markers in brown adipose tissue. Gene expression of indicated mitochondrial markers in (A) male and (B) female mice. (n= 6-8 mice/group). (C) Expression of some of the cluster micro RNAs (miRs) in the brown adipose tissues in WT and lncMGCKO control and HFD male mice. 20 weeks HFD. Statistical analyses were performed by Two-way ANOVA with post-hoc Tukey test for multiple comparisons. The bar and whisker plot displays the distribution of the data. The whiskers extend from the minimum to the maximum values. Individual data points are overlaid as dots. Bar graphs show the mean (SD). Statistically significant p-values are indicated in the bar graphs.

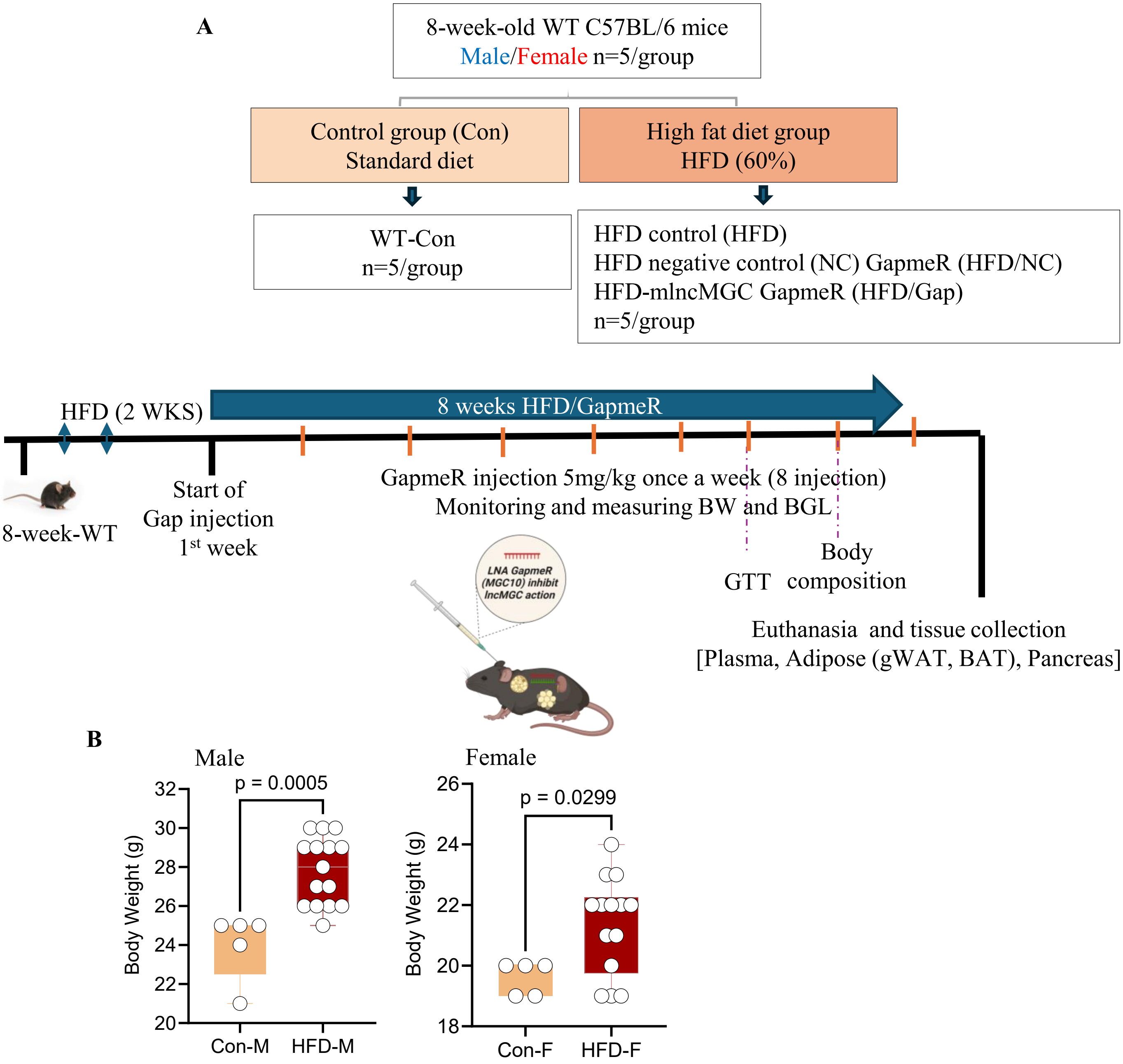

Figure S12. Experimental scheme for treatment with GapmeR targeting mouse lncMGC in high-fat diet (HFD) fed induced- obesity mice model. (A) 8-wk-old female and male wild type (WT) C57BL/6J mice were randomly divided into groups and fed with laboratory control chow diet (Con) or high-fat diet (HFD) (60% kcal from fat) for 2 weeks. Then, mice under HFD were randomly divided into 3 groups and injected with negative control (NC), GapmeR (HFD/NC), or GapmeR targeting mouse lncMGC (HFD/Gap) at 5 mg/kg body weight once a week for 8 weeks. HFD-fed mice without treatment were used as control (HFD). Body weights (BW) and blood glucose levels (BGL) were monitored weekly during 10 weeks of diets. Glucose tolerance tests (GTT) were performed, and body composition was measured at 10 weeks of study. After euthanasia, plasma, perigonadal white adipose tissue (gWAT), and interscapular brown adipose tissue (BAT) and pancreata were harvested. (B) Average body weights after the first 2 weeks of HFD. Statistical comparisons between two groups were performed using two-sided Student's t-tests. The whiskers extend from the minimum to the maximum values. Individual data points are overlaid as dots. P-values are indicated in the bar graphs.

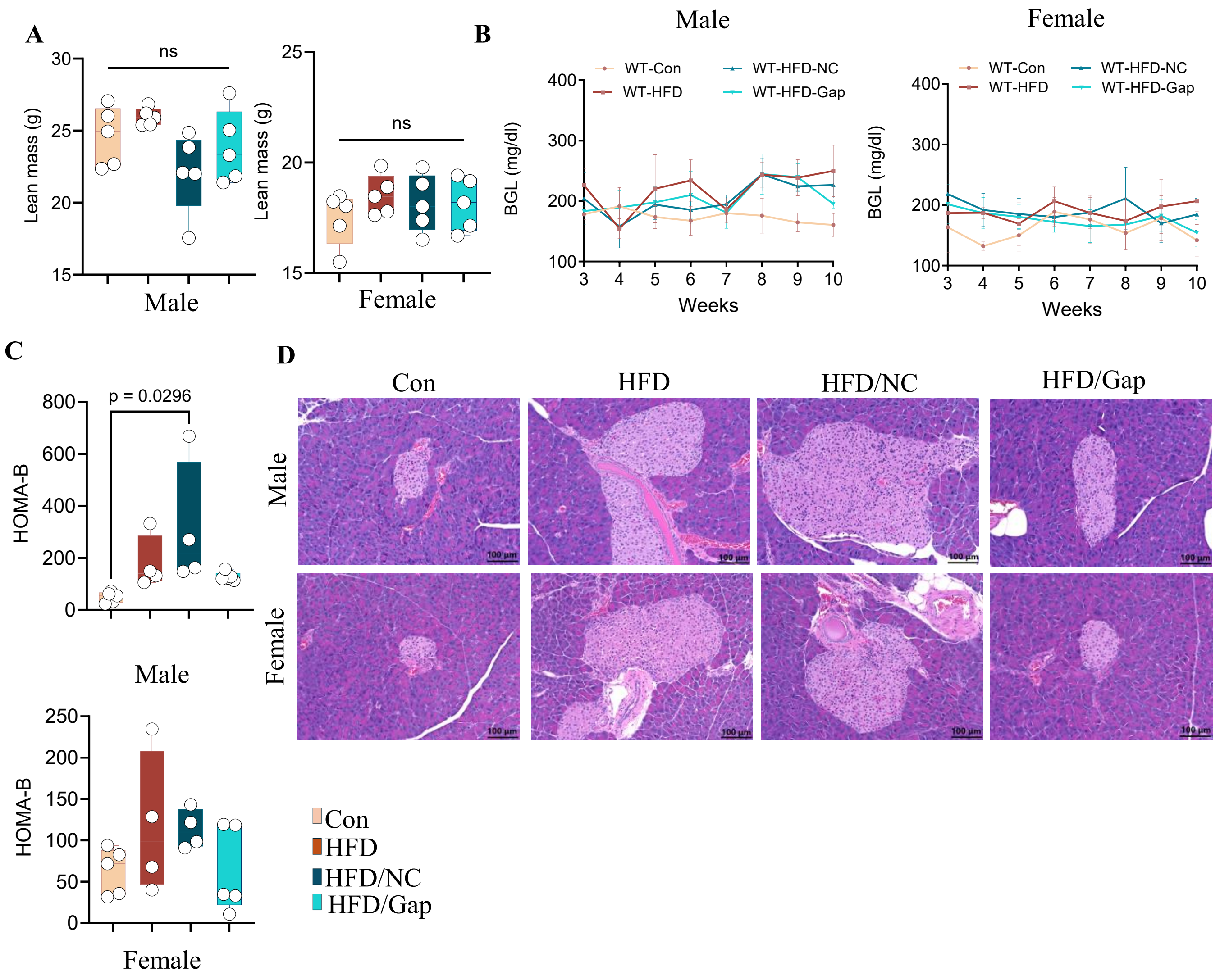

Figure S13. GapmeR targeting lncMGC improved beta cell function in HFD-fed mice. (A) Lean mass in male and female mice at 10 weeks of diets. (B) Average non-fasting blood glucose levels (BGL) of male and female mice over 10 weeks of diets. (C) Percentage of Homeostasis Model Assessment (HOMA) analysis for β-cell function (HOMA-B) in male and female mice. (D) H&E-stained sections showing islets hyperplasia and hypertrophy in HFD and HFD/NC mice and improvement in HFDGap mice. Control chow-diet (Con), high-fat diet (HFD), negative control GapmeR (HFD-NC) and GapmeR targeting lncMGC (HFD-Gap). n=4-5/group. Statistical analyses were performed by Two-way ANOVA with post-hoc Tukey test for multiple comparisons. XY graphs show the mean (SD). The bar and whisker plot displays the distribution of the data. The whiskers extend from the minimum to the maximum values. Individual data points are overlaid as dots. Statistically significant p-values are indicated in the bar graphs.

A

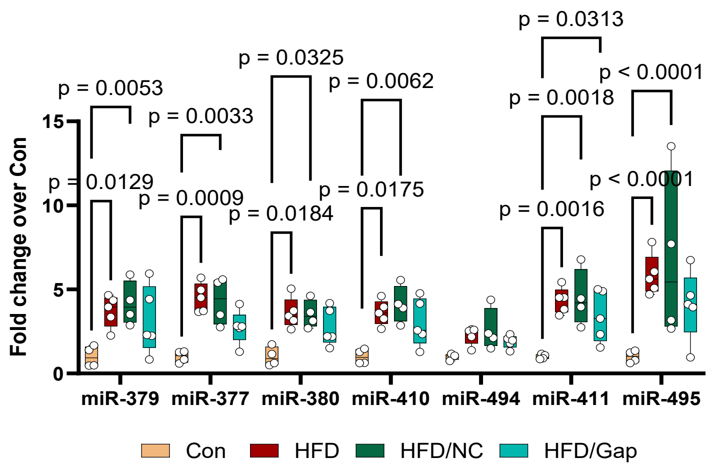

B

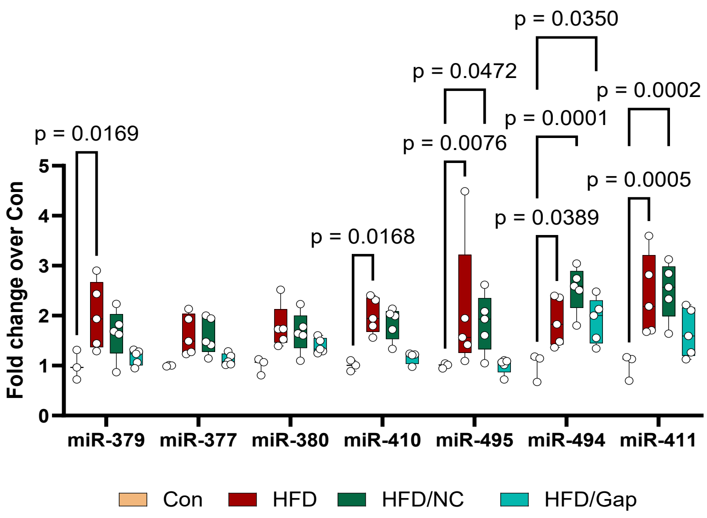

Figure S14. Expression of some of the cluster micro RNAs (miRs) in the perigonadal white adipose tissues from (A) male and (B) female mice treated with or without GapmeRs.10 weeks HFD. Control chow-diet (Con), high-fat diet (HFD), negative control (NC) GapmeR (HFD/NC) and GapmeR targeting lncMGC (HFD/Gap). Statistical analyses were performed by two-way ANOVA with a post-hoc Tukey test for multiple comparisons. n=4-5. The bar and whisker plot displays the distribution of the data. The whiskers extend from the minimum to the maximum values. Individual data points are overlaid as dots. Statistically significant p-values are indicated in the bar graphs.

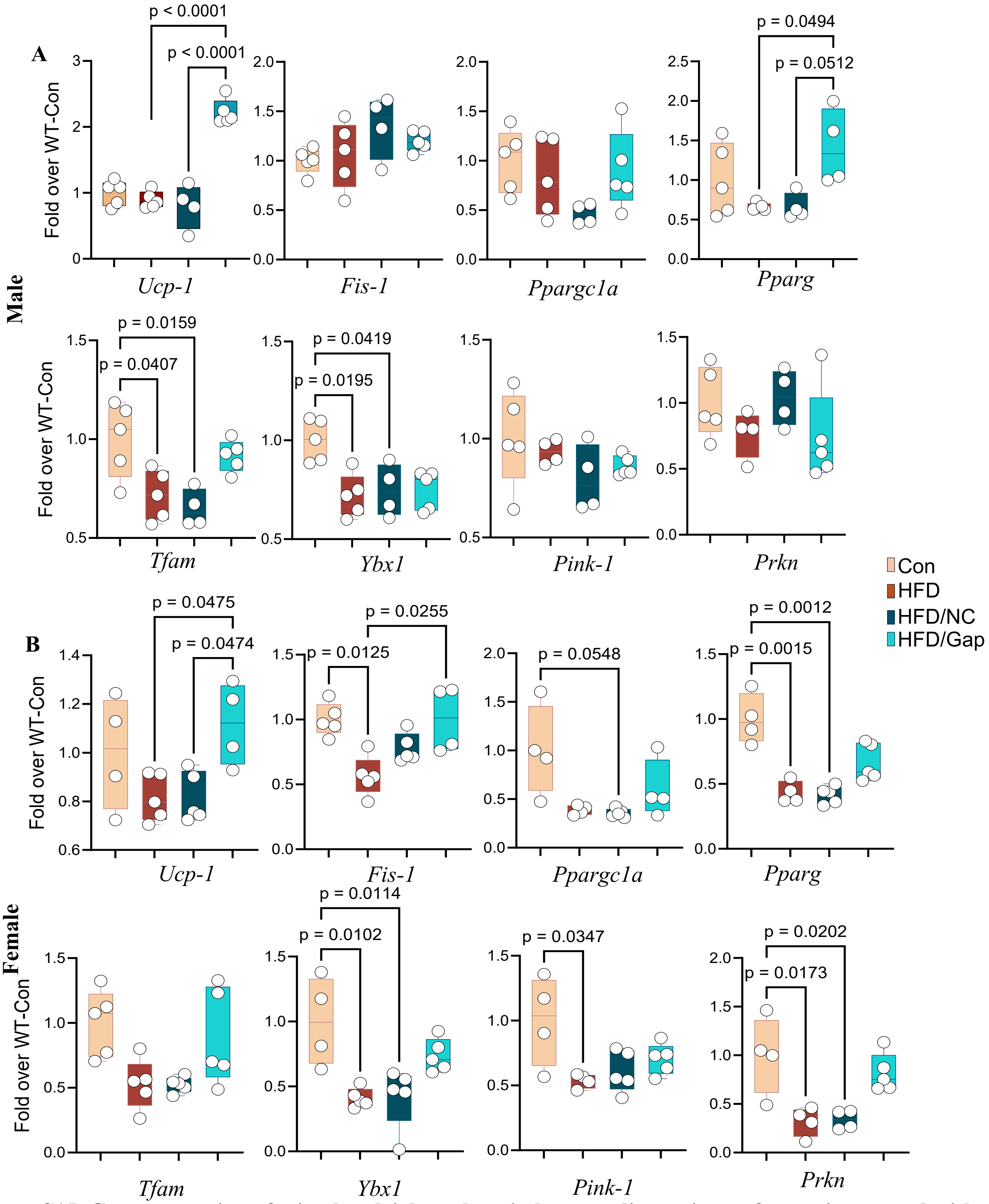

Figure S15. Gene expression of mitochondrial markers in brown adipose tissues from mice treated with or without GapmeRs. Gene expression of mitochondrial markers in (A) male and (B) female mice. 10 weeks HFD. Statistical analyses were performed by Two-way ANOVA with post-hoc Tukey test for multiple comparisons. Control chow-diet (Con), high-fat diet (HFD), negative control (NC) GapmeR (HFD/NC) and GapmeR targeting lncMGC (HFD/Gap). n=4-5. The bar and whisker plot displays the distribution of the data. The whiskers extend from the minimum to the maximum values. Individual data points are overlaid as dots. Statistically significant p-values are indicated in the bar graphs.

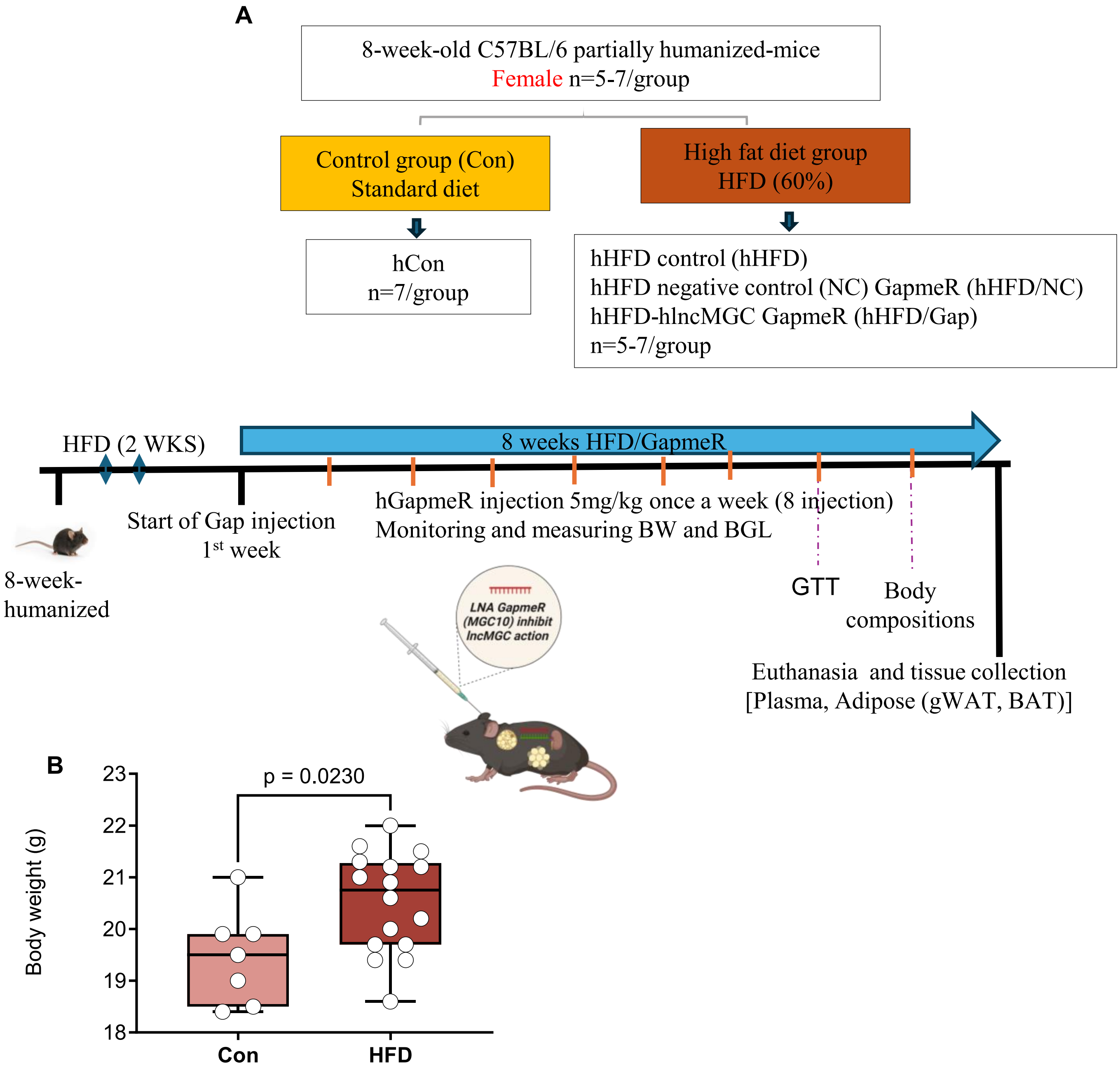

Figure S16. Experimental scheme for treatment with GapmeR targeting human lncMGC in high-fat diet (HFD) fed induced- obesity mice model with partially humanized lncMGC mice. (A) 8-wk-old female partially humanized lncMGC (hlncMGC) mice were randomly divided into groups and fed with laboratory control chow-diet (hCon) or high-fat diet (hHFD) (60% kcal from fat) for 2 weeks, then, hHFD mice were randomly divided into 3 groups and injected with negative control (NC) GapmeR (hHFD/NC) or GapmeR targeting lncMGC (hHFD/Gap) at 5 mg/kg body weight once a week for 8 weeks. hHFD-fed mice without treatment were used as control (hHFD). Body weight (BW) and blood glucose levels (BGL) were monitored weekly during 10 weeks of diets. Glucose tolerance tests (GTT) were performed, and body composition was measured at 10 weeks of study. After euthanasia, plasma, perigonadal white adipose tissue (gWAT), and interscapular brown adipose tissue (BAT) were harvested. (B) Average body weights after the first 2 weeks of HFD. Statistical comparisons between two groups were performed using two-sided Student's t-test. The whiskers extend from the minimum to the maximum values. Individual data points are overlaid as dots. P-values are indicated in the bar graphs.

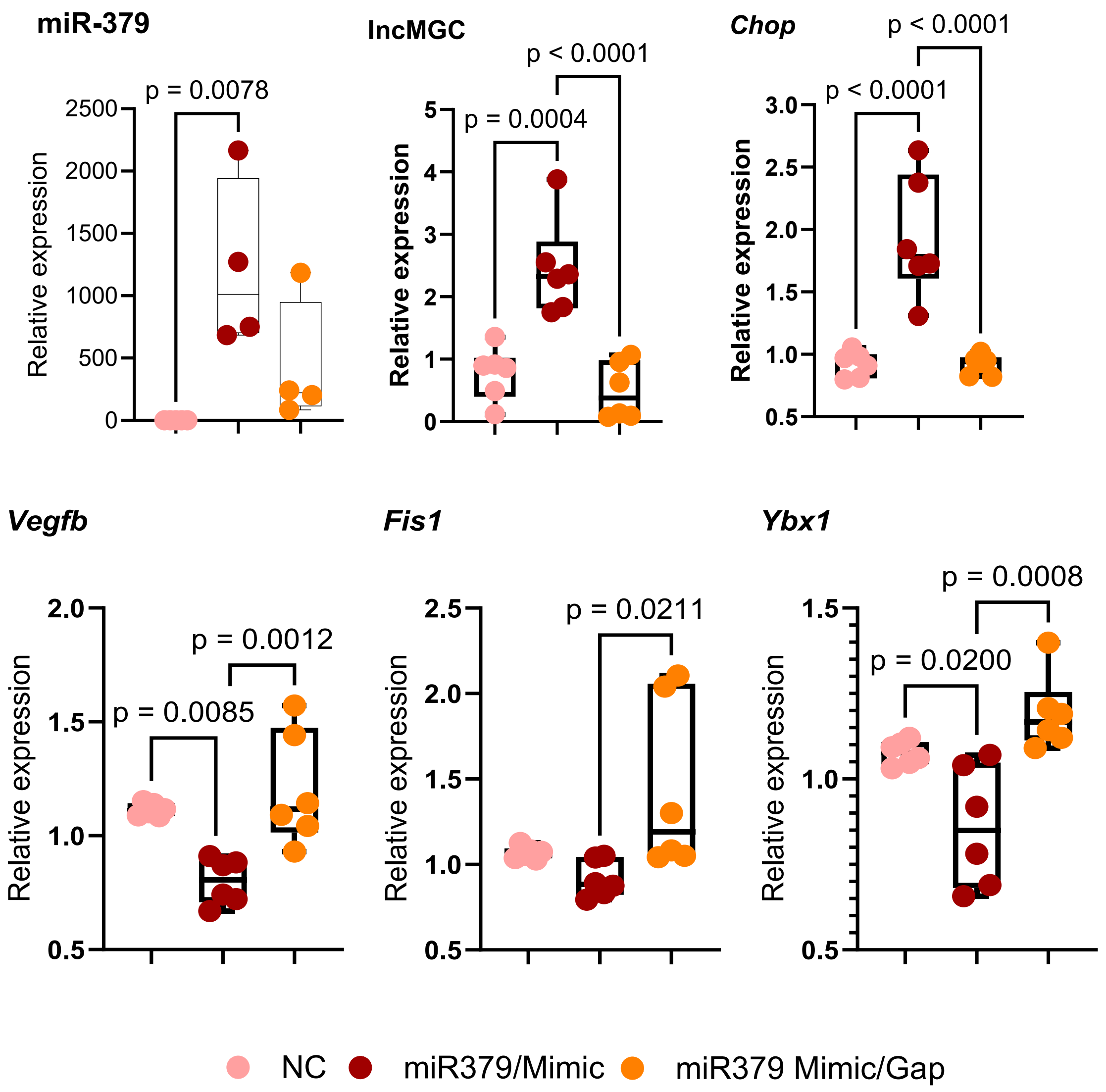

Figure S17. HIB1B brown adipocytes (~3.5×10^5 cells/transfection) were transfected with oligo mimics of miR-379 (50 nmol/L) or the corresponding negative control (NC) oligos (50 nmol/L) using Lipofectamine RNAiMAX. Cells were then treated with lncMGC-GapmeR (2 µmol/L) for 3 days via Gymnosis. Expression of miR-379, lncMGC , *Chop,* and the miR-379 target genes *Vegfb*, *Fis-1,* and *Ybx1*. Statistical analyses were performed by one-way ANOVA with a post-hoc Tukey test for multiple comparisons. n=6.The bar and whisker plot displays the distribution of the data. The whiskers extend from the minimum to the maximum values. Individual data points are overlaid as dots. Statistically significant p-values are indicated in the bar graphs.

Table S1. Clinical parameters of lean, overweight /obese human donors of adipose tissues.

Individual ID		HbA1c %	BMI
Lean	N1	4.4	23.63
	N2	5.1	23.8
	N3	5.3	22.79
	N4	5.2	22
	N5	5.1	20.7
overweight/obese	N1	4.6	36
	N2	5.6	32
	N3	5.3	33
	N4	5.8	29.9
	N5	5.7	26.8
	N6	4.9	25.2
	N7	5.9	31.3
	N8	5.7	32.3
	N9	5.4	33
	N10	4.5	36

The fully de-identified white adipose tissue (WAT) samples were obtained as discard tissues from the Southern California Islet Cell Resource Center (City of Hope) and classified into two groups—lean, and overweight/obese based on the donor's body mass index (BMI).

Table S2. The sequences of PCR primers used in this study.

Target	Forward primer	Reverse primer
mVegfb	GAACCTCATGTCTCACCTCAG	TCATAACAGAACCCAAATCCCG
mTfam	AGC GTG CTA AAA GCA CTG GG	ACT TCG GAA TAC AGA CAA GAC TGA
mFis1	ACCATCGCCTTCCCTTTTC	ATGACAGGGTTAAACGACAGG
mYbx1	GCA GAC CGT AAC CAT TAT AGA CG	TCT CCG CAT GTA GTA AGG TGG
mChop (Ddit)	GCACCTATATCTCATCCCCAG	TGCGTGTGACCTCTGTTG
mPpargc1a (Pgc1)	CACCAAACCCACAGAAAACAG	GGGTCAGAGGAAGAGATAAAGTTG
mPrkn	GCA CAC CCA ACC TCA GAC AA	TCA GTG GAG ATG AGG CCG A
mPink1	GTG GGA CTC AGA TGG CTG TC	ACT GGA GCT GTT GAA AGG CAG
mCebpb	AGCCCCTACCTGGAGCCGCTCGCG	GCGCAGGGCGAACGGGAAACCG
mPparg	TGTTATGGGTGAAACTCTGGG	AGAGCTGATTCCGAAGTTGG
mlncMGC	ATTTTTCTGAGTTAGTGTGGCCTTCATCTG	TCAGGAACCATGGAACGGTGTTGACCCCTAG
mCypa	ATGGTCAACCCCACCGTGT	TTCTTGCTGTCTTTGGAACTTTGTC
hlncMGC	GCCTGCTTCCAATGCCAAATC	CTTCAGGAACCACGGAATGGT
hCYPA	CCCACCGTGTTCTTCGACATT	GGACCCGTATGCTTTAGGATGA

Table S3: **Sample ID, ROI selected area, and nuclei count per ROI**.

Sample_ID	ROI	Segment	Area	Nuclei	(v1.0) Mouse NGS Whole Transcriptome Atlas RNA
WT Con	001	Full ROI	97592.29	290	MW0031123
WT Con	002	Full ROI	97592.29	392	MW0031123
WT Con	003	Full ROI	97592.29	221	MW0031123
WT Con	004	Full ROI	97592.29	217	MW0031123
WT HFD	005	Full ROI	97592.29	129	MW0031123
WT HFD	006	Full ROI	97592.29	168	MW0031123
WT HFD	007	Full ROI	97592.29	174	MW0031123
WT HFD	008	Full ROI	97592.29	158	MW0031123
WT HFD	009	Full ROI	97592.29	206	MW0031123
WT HFD	010	Full ROI	97592.29	170	MW0031123
lncMGC KO Con	011	Full ROI	97592.29	323	MW0031123
lncMGC KO Con	012	Full ROI	97592.29	360	MW0031123
lncMGC KO Con	013	Full ROI	97592.29	345	MW0031123
lncMGC KO Con	014	Full ROI	97592.29	330	MW0031123
lncMGC KO Con	015	Full ROI	97592.29	365	MW0031123
lncMGC KO HFD	016	Full ROI	97592.29	223	MW0031123
lncMGC KO HFD	017	Full ROI	97592.29	274	MW0031123
lncMGC KO HFD	018	Full ROI	97592.29	258	MW0031123
lncMGC KO HFD	019	Full ROI	97592.29	282	MW0031123
lncMGC KO HFD	020	Full ROI	97592.29	263	MW0031123
lncMGC KO HFD	021	Full ROI	97592.29	311	MW0031123
lncMGC KO HFD	022	Full ROI	97592.29	269	MW0031123

Sample ID, ROI selected area, and nuclei count per ROI. from brown adipose tissues in wild type control (WT Con), WT-high fat diet (HFD), lncMGCKO Con and lncMGCKO HFD female mice.
ROI: Region of interest

Table S4: **ER-stress related genes.**

Gene ID	Log2FC WT HFD vs WT Con	P value	Log2FC KO HFD vs KO Con	P value
Hspa1a	1.3299	0.0086	0.6337	0.6105
App	0.7965	0.0063	0.4292	0.0001
Ccnd1	0.5279	0.0139	0.7302	0.0003
Nck1	0.4272	0.0137	0.3314	0.0095
Bcl2l1	0.3983	0.0023	0.2974	0.0727
Trim25	0.2849	0.0493	0.1361	0.1540
Hsp90b1	0.2661	0.0296	-0.1852	0.0325
Ddit3	0.2602	0.0566	-0.0335	0.8923
Ube4b	0.2532	0.0728	-0.0013	0.9915
Ppp2cb	0.2467	0.0696	0.0786	0.4564
Serinc3	0.2446	0.0258	0.1418	0.1631
Sgf29	0.2434	0.0445	0.1445	0.2667
Ubxn4	0.2127	0.0805	-0.1772	0.4392
Hspa5	0.2038	0.0277	-0.1657	0.1423

Differential expression of ER stress–related genes in brown adipose tissue from WT and lncMGC-KO mice. Using RNA-seq analysis, genes associated with endoplasmic reticulum (ER) stress were upregulated in WT-HFD mice compared with controls. Expression of these ER stress–related genes was attenuated in lncMGC KO HFD mice relative to lncMGC KO controls. The table summarizes fold-change (FC) values for ER stress–related genes.
